# miR-10a-5p and miR-29b-3p as Extracellular Vesicle-Associated Prostate Cancer Detection Markers

**DOI:** 10.3390/cancers12010043

**Published:** 2019-12-21

**Authors:** Thomas Stefan Worst, Christopher Previti, Katja Nitschke, Nicolle Diessl, Julia Christina Gross, Lena Hoffmann, Lisa Frey, Vanessa Thomas, Christoph Kahlert, Karen Bieback, Adriana Torres Crigna, Fabia Fricke, Stefan Porubsky, Niklas Westhoff, Jost von Hardenberg, Philipp Nuhn, Philipp Erben, Maurice Stephan Michel, Michael Boutros

**Affiliations:** 1 Department of Urology and Urosurgery, Medical Faculty Mannheim, Heidelberg University, 68167 Mannheim, Germany; katja.nitschke@medma.uni-heidelberg.de (K.N.); lena.hoffmann@medma.uni-heidelberg.de (L.H.); lisa.frey@stud.uni-heidelberg.de (L.F.); v.c.thomas@web.de (V.T.); niklas.westhoff@medma.uni-heidelberg.de (N.W.); jost.vonhardenberg@medma.uni-heidelberg.de (J.v.H.); philipp.nuhn@medma.uni-heidelberg.de (P.N.); philipp.erben@medma.uni-heidelberg.de (P.E.); maurice-stephan.michel@medma.uni-heidelberg.de (M.S.M.); 2 Division of Signaling and Functional Genomics, German Cancer Research Center (DKFZ), 69120 Heidelberg, Germany; m.boutros@dkfz-heidelberg.de; 3 Omics IT and Data Management Core Facility (ODCF), German Cancer Research Center (DKFZ), 69120 Heidelberg, Germany; christopher.previti@dkfz-heidelberg.de; 4 Genomics and Proteomics Core Facility, German Cancer Research Center (DKFZ), 69120 Heidelberg, Germany; nicolle.diessl@dkfz-heidelberg.de; 5 Departments of Haematology and Oncology and Developmental Biochemistry, University Medicine Göttingen, 37075 Göttingen, Germany; julia.gross@med.uni-goettingen.de; 6 Department of Gastrointestinal, Thoracic and Vascular Surgery, University Hospital Carl Gustav Carus, Technische Universität Dresden, 01307 Dresden, Germany; christoph.kahlert@uniklinikum-dresden.de; 7 Institute of Transfusion Medicine and Immunology, FlowCore Mannheim, Medical Faculty Mannheim, Heidelberg University, 68167 Mannheim, Germany; karen.bieback@medma.uni-heidelberg.de; 8 Institute of Transfusion Medicine and Immunology, Medical Faculty Mannheim, Heidelberg University, 68167 Mannheim, Germany; adriana.torrescrigna@medma.uni-heidelberg.de; 9 Department of Applied Tumor Biology, Institute of Pathology, Heidelberg University Hospital, 69120 Heidelberg, Germany; f.fricke@dkfz-heidelberg.de; 10 Clinical Cooperation Unit Applied Tumor Biology, German Cancer Research Centre (DKFZ), 69120 Heidelberg, Germany; 11 Institute of Pathology, Medical Faculty Mannheim, Heidelberg Heidelberg, 68167 Mannheim, Germany; stefan.porubsky@umm.de

**Keywords:** prostate cancer, extracellular vesicles, next generation sequencing, qRT-PCR, non-coding RNA, miRNA, mir-10a-5p, miR-29b-3p, miR-99b-5p

## Abstract

Extracellular vesicles (EVs) are shed by many different cell types. Their nucleic acids content offers new opportunities for biomarker research in different solid tumors. The role of EV RNA in prostate cancer (PCa) is still largely unknown. EVs were isolated from different benign and malignant prostate cell lines and blood plasma from patients with PCa (*n* = 18) and controls with benign prostatic hyperplasia (BPH) (*n* = 7). Nanoparticle tracking analysis (NTA), Western blot, electron microscopy, and flow cytometry analysis were used for the characterization of EVs. Non-coding RNA expression profiling of PC3 metastatic PCa cells and their EVs was performed by next generation sequencing (NGS). miRNAs differentially expressed in PC3 EVs were validated with qRT-PCR in EVs derived from additional cell lines and patient plasma and from matched tissue samples. 92 miRNAs were enriched and 48 miRNAs were depleted in PC3 EVs compared to PC3 cells, which could be confirmed by qRT-PCR. miR-99b-5p was significantly higher expressed in malignant compared to benign EVs. Furthermore, expression profiling showed miR-10a-5p (*p* = 0.018) and miR-29b-3p (*p* = 0.002), but not miR-99b-5p, to be overexpressed in plasma-derived EVs from patients with PCa compared with controls. In the corresponding tissue samples, no significant differences in the miRNA expression could be observed. We thus propose that EV-associated miR-10a-5p and miR-29b-3p could serve as potential new PCa detection markers.

## 1. Introduction

Extracellular vesicles (EVs) are defined as particles delimited by a lipid bilayer that cannot replicate and can be subclassified according to their size, density, biochemical composition, and origin [1]. In addition to lipids, they contain proteins, RNA, and DNA [2]. They are involved in many different processes in both paracrine and long-range cell-to-cell communication [3]. Active surface proteins give them antigenic potency, capable of both inducing immune response and interacting with cellular surface structures [4,5,6]. When taken up, their biologically active contents are released, enabling various effects on transcription, translation, and post-translational modifications, leading to significant changes in the metabolism and homeostasis of the recipient cell [7,8,9]. Apart from their physiological functions, for instance, in immune-modulation, angiogenesis, and proliferation, they are crucial to many pathophysiological processes [10,11], like the modification of the tumor microenvironment in the paracrine setting [12,13] or pre-metastatic niche formation via the blood stream [14]. In many publications, small EVs in the size range between 30 and 100 nm are termed as exosomes, though a clear differentiation from other EVs regarding the composition and function is not possible [1].

Owing to their functions and their presence in body fluids, EVs have become an area of growing interest for functional studies and biomarker discovery. Proteomic screening methods, such as mass spectrometry, have recently been used to identify several exosomal proteins as potential biomarkers for different malignant and non-malignant diseases [15,16]. For instance, exosomal glypican 1 has emerged as a promising marker for pancreatic adenocarcinoma [17]. Also, in prostate cancer (PCa), several studies have performed proteomic profiles of EVs [18,19,20,21]. Our own group recently identified Claudin 3 as a potential EV-based biomarker in PCa [22]. Besides proteins, nucleic acids are the major class of molecules used in EV-based biomarker studies. Especially, next generation sequencing (NGS) techniques have helped to overcome limitations of initial transcriptomic studies of EVs with expression microarrays, such as the necessity of a high sample input amount and the inability to detect undescribed RNA molecules [23]. Despite the rapid advances in NGS techniques [24], however, RNA sequencing of EVs is not yet a standard laboratory practice, and there are only a limited number of studies using this approach in PCa. Nevertheless, sequencing studies of EVs have recently reported promising results; for example, in patients with PCa, Miranda et al. revealed a predominance of non-coding RNA (ncRNA) in urinary EVs [25] and Rodriguez et al. described miR-196a-5p and miR-501-3p as potential urine-based biomarkers [26]. Furthermore, Huang et al. identified miR-375 and miR-1290 as potential EV-based biomarkers in the circulation of patients with metastatic PCa [27] and Sánchez et al. found a different miRNA content in EVs of cancer stem cells compared with bulk cancer cells [28]. Typically, NGS approaches focus on mRNA analysis or on certain small RNA classes, like miRNAs, limiting the information about other ncRNAs. Furthermore, when using genomic mapping of sequencing reads, especially for ncRNAs, multiple mapping occurs frequently. This results in ambiguous information about the identity of specific reads and can perturb the assessment of the read distribution between different RNA classes. We implemented a customized multi-step analysis pipeline, mapping against specific ncRNA databases, into a streamlined protocol using a cell culture-based approach and state-of-the-art EV isolation to profile the ncRNA content of PC3 prostate cancer EVs in comparison with their parental cells. qRT-PCR served for technical validation of our findings. Subsequently, potential biomarker candidates were retested in EVs of the blood plasma of patients with PCa and benign prostatic hyperplasia (BPH), as well as in three corresponding tissue samples.

## 2. Results

### 2.1. Isolated Particles Show Typical EV Features

**Figure 1:**
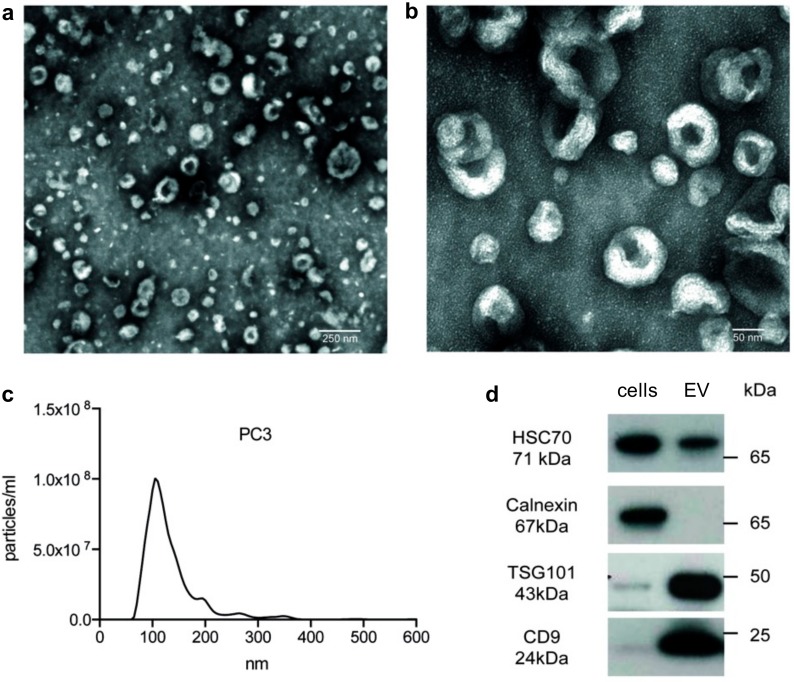
Quality control of isolated prostate cancer (PC)3 extracellular vesicles (EVs). (**a**,**b**) Transmission electron microscopy (TEM) confirmed the presence of EVs in the isolates from PC3 supernatant. (**c**) Using nanoparticle tracking analysis (NTA), a typical size distribution of isolated EVs could be seen. (**d**) Western blotting showed a typical marker constellation on PC3 EVs (HSC70^+^, CD9^+^, TSG101^+^, Calnexin^−^).

In order to confirm the presence of EVs in the isolates obtained from cell culture supernatant and to assess the purity of EVs, samples were subjected to a three-step quality control. Transmission electron microscope (TEM) of EVs from the metastatic PC3 cell line, intended to be used for subsequent NGS analysis, revealed small cup-shaped vesicles in a size ranging between 50 and 200 nm (Figure 1a,b), which is in accordance with other reports [1,29,30]. Using nanoparticle tracking analysis (NTA) of these samples, a narrow distribution curve in the range between 50 and 200 nm was seen (Figure 1c). Immunoblotting of PC3 cells and EVs confirmed the presence of the typical EV markers CD9 (cluster of differentiation protein 9, tetraspanin 29), TSG101 (tumor susceptibility gene 101 protein), and HSC70 (heat shock protein 70) and the absence of the cellular/ER marker Calnexin on cell culture EVs (Figure 1d). The detailed results of immunoblotting are shown in Figure S1.

### 2.2. ncRNAs Are Differentially Enriched in PC3 EVs

We performed small RNA sequencing in order to characterize the RNA content of PC3 EVs and their parental cells. RNA was isolated from cellular and EV samples and subjected to quality assessment using the Agilent 2100 Bioanalyzer RNA 6000 Pico and small RNA chips. Both sample types had their main peak in a fragment range of around 150 nt, but in EVs, a higher proportion of shorter RNA fragments could be seen (Figure S2).

Library preparation resulted in cDNA peaks in the range between 140 und 329 bp, being in accordance with the expected size distribution of raw libraries given by the manufacturer. Sequencing of pooled 10 nM libraries resulted in 1.59 to 7.36 million reads mappable to the ncRNA reference databases per sample.

Bioinformatic analysis confirmed a high correlation between sample replicates (Pearson correlation coefficient *r*: 0.96 for cells and 0.97 for EVs), whereas only moderate correlations were obtained between cellular and EV samples *(r* ranging between 0.67 and 0.69, Figure S3). Comparison of the cellular and EV datasets revealed a relevant proportion of differentially enriched ncRNAs, pointing to a partially selective loading of ncRNA molecules into PC3 EVs (Figure S4).

### 2.3. Distinct ncRNA Distribution in PC3 Cells and EVs

**Figure 2:**
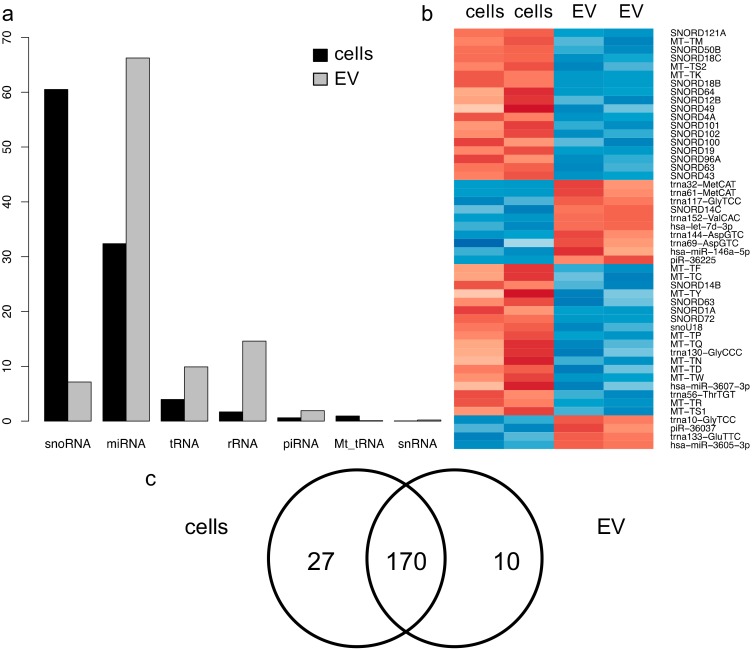
Differential enrichment of ncRNAs in PC3 cells and EVs. (**a**) Cellular samples were dominated by snoRNA, while mature miRNAs were the largest group of ncRNAs in PC3 EVs (*n* = 2 per cells and EVs, each). (**b**) The 50 most significantly differentially enriched small ncRNAs are shown in a heatmap (red = enriched in EVs, blue = depleted in EVs). (**c**) Overlap of miRNAs detected in PC3 cells and EVs with a threshold of an average of 100 mappable reads.

Next, we asked which ncRNA classes were abundant in PC3 cells and EVs. Figure 2a shows the distribution of different ncRNA classes in two biological replicates of PC3 cells and EVs. Predominant ncRNA classes in EVs were mature miRNAs (66.25%), rRNA (14.57%), and tRNA (9.88%). In PC3 cells, a relevant amount of miRNA (32.38%) was also seen, but the predominant group was snoRNA (60.50%), with only a limited amount of piRNA, rRNA, snRNA, and tRNA. Furthermore, a small amount of mitochondrial tRNA (Mt_tRNA) was seen in cells, but not in EVs. The ncRNA distribution in all single replicates is given in Table 1.

According to the different distribution of ncRNA classes, distinct profiles could also be seen on the single RNA level. Looking at the 50 most differentially enriched ncRNAs, mainly snoRNAs and Mt-tRNAs clustered to the two cellular samples (Figure 2b). Additionally, one miRNA, namely miR-3607-3p, was highly overrepresented in PC3 cells compared with EVs. The ncRNAs most selectively enriched in EVs were mainly tRNAs, as well as piRNA-36225, piRNA-36037, miR-let7d-3p, miR-146a-5p, and miR-3605-3p. A complete overview of all identified ncRNAs in cellular and EV samples, including normalized read counts and statistics, is given in Table S1.

### 2.4. PC3 Cells and EVs Have Distinct miRNA Profiles

Further analyses focused on miRNA, as they are biologically the most potent and relevant of the identified ncRNA classes owing to their well-documented pleiotropic regulatory effects and the fact that there are many verified assays available, making them most suitable for the technical validation of our findings.

Distinct miRNA patterns in PC3 cells and EVs were obtained. In the whole dataset, 238 different miRNAs were identified. A total of 170 miRNAs showed an abundance of at least 100 mappable reads in both PC3 cells and EVs, 27 were above this threshold only in PC3 cells, and 10 only in PC3 EVs. Thirty-one miRNAs with less than 100 reads were detected in both cells and EVs (Figure 2c). Ninety-two miRNAs showed a significant enrichment in EVs (*p* < 0.05). Of these, 53 were enriched at least four-fold. Forty-eight miRNAs were depleted in EVs (*p* < 0.05), with nine of them depleted at least four-fold. The 15 miRNAs most highly enriched in EVs are shown in Table 2. Table 3 summarizes the 15 most depleted miRNAs in EVs. A complete list of all identified miRNAs is given in Table S2. Interestingly, there were 10 members of the miR-10 family, which is known to mostly have tumor suppressive function in solid tumors [31,32,33], enriched in EVs (Table 4). No member of this family was depleted in EVs.

### 2.5. qRT-PCR Shows Similar miRNA Signatures in Other Prostate Cell Line EVs

**Figure 3:**
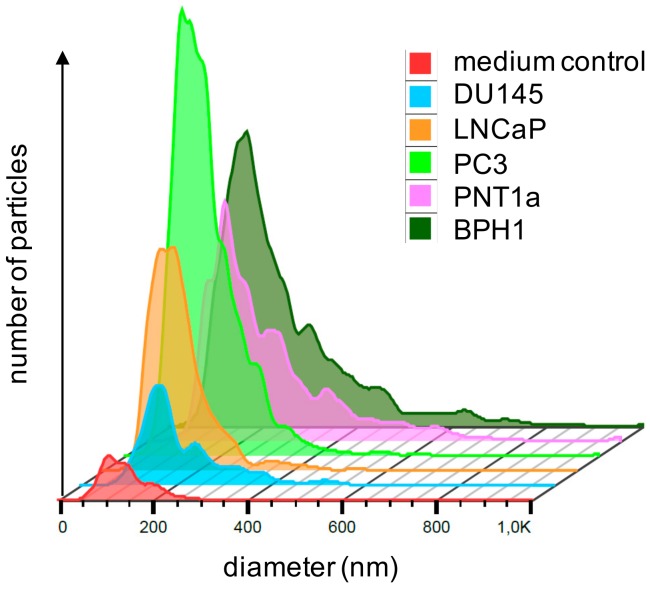
Exemplary single Nanoparticle tracking analysis (NTA) of EVs from all five cell lines and medium control showed a typical size distribution. Owing to varying sample dilutions, necessary to achieve optimal measuring conditions, particle counts are not directly comparable in this graph. Quantitative data are given in Table 5. BPH, benign prostatic hyperplasia.

**Figure 4:**
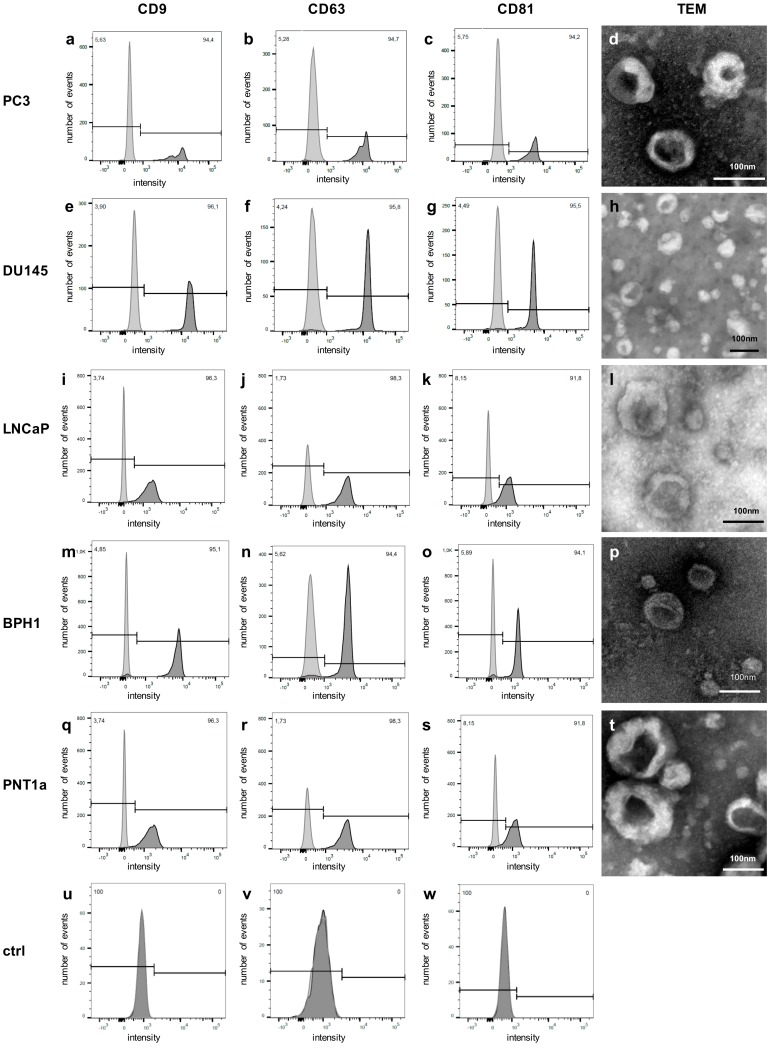
EVs from PC3 and all four validation cell lines showed positive signals for CD9, CD63, and CD81 and typical cup shaped vesicles in TEM (PC3: **a–d**; DU145: **e–h**; LNCaP: **i–l**; BPH1: **m–p**; PNT1a: **q–t**). **u–w**: Particles isolated from medium controls showed no detectable signals for CD9, CD63, and CD81. In TEM, no cup-shaped particles were detectable (flow cytometry analyses: light grey: unstained control; dark grey: stained).

For validation, EVs were again isolated from PC3 cells and from two other metastatic PCa cell lines (DU145, LNCaP), as well as from two benign prostate cell lines (BPH1, PNT1a). After harvesting EVs, the parental cells showed viability rates above 90%, except for benign PNT1a cells with 82.4%. The EVs of all cell lines showed a typical size distribution (Figure 3). In the control samples derived from EV production, the medium was incubated for 72 h in the absence of cells, and particles in the same size range could also be detected. Yet, the concentration of the isolated particles was 2–3 log10-doses lower compared with cell culture EVs (Table 5). The isolated EVs from all five cell lines were positive for the EV markers CD9, CD63, and CD81 in bead-based flow cytometry analyses and showed a cup shaped morphology in TEM (Figure 4a–t). In the medium controls, none of the EV markers could be detected (Figure 4u–w). In TEM, no particle structures could be observed.

In addition to flow cytometry analysis, Western blot analysis of cells and EVs confirmed expression of the two membrane tetraspanins CD63 and CD9 in the EV samples. Moreover, the three cytosolic proteins Alix, TSG101, and Syntenin were found to be enriched in the EVs compared with their parental cells. Cytoskeletal β-actin was exclusively detected in the cell lysates, but not in EV samples, excluding contamination of EV lysates with cells or cellular debris (Figures S5–S9). In the medium controls, none of the EV protein markers and cytoskeletal proteins were detected.

**Figure 5:**
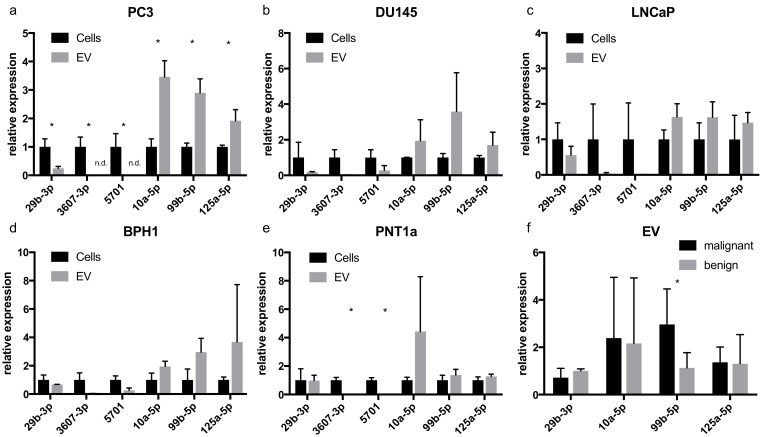
(**a**–**e**) miRNA expression in cells and EVs of prostate cell lines measured by qRT-PCR (*n* = 3 biological replicates per cell line). Tumor cell lines (PC3, DU145, and LNCaP) showed a similar expression of miR-10a-5p, miR-99b-5p, and miR-125a-5p. (**f**) When comparing expression in EVs of malignant and benign cell lines only for miR-99b-3p, a significant difference was seen. All cell lines showed a comparably low expression of miR-3607-3p and miR-5701, hence a comparison of these two miRNAs between malignant and benign EVs was not possible. The data are shown as relative expression according to the 2^−ΔΔCT^-method. Data are given as means with SD (* *p* < 0.05).

Three miRNAs enriched in PC3 EVs (miR-10a-5p, miR-99b-5p, and miR-125a-5p), three miRNAs depleted in PC3 EVs (miR-29b-3p, miR-3607-3p, and miR-5701), and two miRNAs showing no enrichment according to our PC3 sequencing data (miR-20a-5p and miR-28-5p) were retested using qRT-PCR (three independent biological replicates per cell line) in metastatic PC3 cells and the four additional cell lines (DU145, LNCaP, BPH1, PNT1a). In PC3 EVs, qRT-PCR confirmed an enrichment of miRNAs similar to the sequencing results. MiR-10a-5p (3.46-fold, *p* = 0.003), miR-99b-5p (2.90-fold, *p* = 0.003), and miR-125a-5p (1.92-fold, *p* = 0.016) were significantly enriched. MiR-3607-3p (*p* = 0.007) and miR-5701 (*p* = 0.021) were not detected in PC3 EVs and miR-29b-3p (0.25-fold, *p* = 0.010) had a significantly lower abundance compared with reference miRNAs than in cellular samples (Figure 5a). These results seem to confirm a selective enrichment of different miRNAs in PC3 cells and EVs. To test whether this effect is restricted to PC3 or can also be observed in other cell culture model systems, we additionally profiled metastatic DU145 and LNCaP cells and EVs (Figure 5b,c). In these cell lines, the expression of miR-10a-5p, miR-99b-5p, and miR-125a-5p tended to be higher in EVs, whereas the expression of miR-29b-3p, miR3607-3p, and miR-5701 was lower in EVs. None of these differences reached significance. A similar expression pattern was also seen in benign cell lines BPH1 and PNT1a (Figure 5d,e). Yet, except for miR-3607-3p and miR-5701, which were not detectable in PNT1a EVs (*p* = 0.001 and *p* < 0.001), no significant differences were seen. miR99b-5p showed a higher abundance in EVs from malignant compared with EVs from benign cell lines (2.96-fold, *p* = 0.027, Figure 5f). The same was seen when comparing miRNA expression in malignant and benign parental cells (Figure S10).

### 2.6. Validation Shows Overexpression of miR-10a-5p and miR-29b-3p in EVs Derived from PCa Patient Plasma

**Figure 6:**
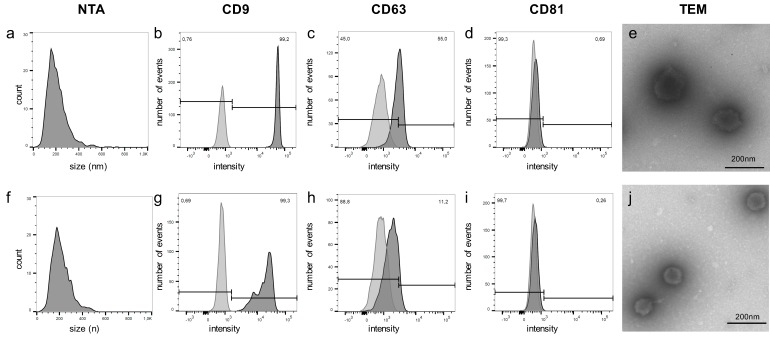
NTA, flow cytometry data, and TEM of EVs of a patient with PCa (**a**–**e**) and a BPH control patient (**f**–**j**).

The same miRNAs were retested using qRT-PCR in EVs from blood plasma of patients with PCa and BPH. The isolated particles showed typical EV features regarding size distribution, CD9 and CD63 expression, and morphology in TEM. CD81 was only scarcely present in some patients. Exemplary quality control results of plasma EVs of a patient with PCa and a patient with BPH are shown in Figure 6. Basic characteristics of the analyzed patients are given in Table 6.

**Figure 7:**
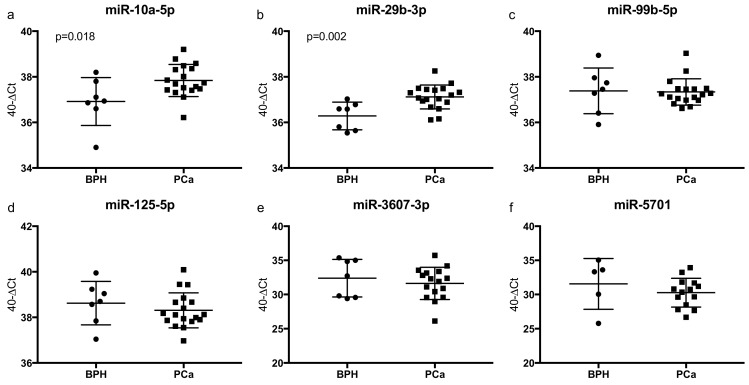
(**a**–**f**) qRT-PCR miRNA expression data of plasma EVs of patients with PCa (*n* = 18) and BPH (*n* = 7) using the 40-ΔCt-method, in which an increment of 1 in the y-axis equals a doubled expression. Higher values correspond to a higher expression and each dot reflects a single patient. The results are given as mean with SD. miR-5701 expression could only be determined in five BPH patients.

The results of the qRT-PCR expression analysis of the six target miRNAs are shown in Figure 7. The two miRNAs miR-10a-5p and miR-29b-3p were significantly higher expressed in EVs of patients with PCa compared with BPH. All other miRNAs did not show significant differences in expression. The expression of none of the miRNAs correlated significantly with the serum PSA levels. To further evaluate if differences in miRNA expression could also be observed on the tissue level, qRT-PCR was also performed on dissected formalin-fixed paraffin-embedded (FFPE) samples, if available (*n* = 14 patients with PCa and *n* = 6 BPH controls). None of the analyzed miRNAs were differentially expressed in the tissue samples (Figure S11).

## 3. Discussion

We used NGS profiling of small ncRNA to identify 92 miRNAs that were enriched and 48 miRNAs that were depleted in PC3 EVs. The largest group of miRNAs that appeared to be enriched in EVs was the miR-10 family. The miR-10 family consists of miRNAs originating from eight different miRNA stem loops. According to miRBase v21, the miR-10 family comprises miRNAs originating from the miR-10a, miR-10b, miR-99a, miR-99b, miR-100, miR-125a, miR-125b-1, and miR-125b-2 stem loops. Among these, miR-10a, miR-10b, and miR-100 are the most conserved and most intensely studied. Furthermore, they are known to be regulated by P65 and TWIST. Physiologically, miR-10 is known to be involved in angiogenesis [34], transcription actin cytoskeleton, and ephrin receptor signaling [35]. With regard to different epithelial cancer entities, miR-10 family members are mostly described as tumor suppressors and described as being underexpressed in advanced cancer stages. Decreased expression of miR-10b was described as a predictor of poor prognosis in renal cell carcinoma [36]. MiR-100 and miR-99a were found to be downregulated in head and neck squamous cell carcinoma [37]. Restoration of miR-100 levels suppressed tumor cell proliferation and migration and enhanced apoptosis. MiR-125b, miR-99a, miR-99b, and miR-100 were found to be downregulated in advanced prostate cancer cell lines, compared with parental cell lines. Transfection of these miRNAs into prostate cancer cells inhibited growth and decreased the expression of prostate specific antigen (PSA) via posttranscriptional regulation [32]. The authors suggested the chromatin-remodeling factors SMARCA5 and SMARCD1 and the growth regulatory kinase mTOR as potential targets for these miRNAs.

All six miRNAs, which were differentially enriched in the PC3 sequencing data, could be independently validated by qRT-PCR in PC3 cells and EVs, confirming the accuracy and validity of our sequencing approach. In the other analyzed cell lines, qRT-PCR expression results showed a stronger inter-replicate variation. In general, miR-3607-5p and miR-5701 were lowly expressed in EVs of all prostate cell lines, so their depletion might be a general phenomenon in EVs. miR29b-3p showed a significantly lower expression in PC3 EVs, whereas in the other cell lines, no significant differences were seen. Yet, the median expression was always lower or similar to the parental cells. MiR-10a-5p showed a tendency of a higher expression in all analyzed cell lines, yet significance was only reached in PC3. For miR99b-5p and miR-125a-5p, the general trend in expression was similar, again without significance.

Validation in patient plasma samples showed miR-10a-5p to be significantly overexpressed in EVs from patients with PCa. The other two miRNAs enriched in PC3 EVs did not show a differing expression in clinical samples, but interestingly, miR-29-3p was also overexpressed in EVs of patients with PCa. For both miR-10a-5p and miR-29-3p, there are only limited data in the context of EVs or PCa. miR-10a was recently described to be elevated in plasma EVs with papillary thyroid carcinoma (PTC), but not in PTC tissue samples [38]. Furthermore, miR-10a seems to be involved in glioma-induced modulation of the tumor microenvironment [39]. Another study found miR-10a to be enriched in microvesicles of mesenchymal stromal cells of patients with myelodysplastic syndrome [40]. Tumor suppressive effects were shown for miR-10a-5p in both breast cancer and hepatocellular carcinoma [41,42]. In breast cancer, these effects have been shown to be mediated by PI3K/Akt-signaling [41,43]. Comparable studies in PCa are missing. miR-29 was found to be associated with cardiac stem cells and a cardioprotective effect [44], and to reduce muscle atrophy and renal fibrosis in mice [45]. In addition, the miR-29 family was found to be associated with EVs in chronic lymphatic leukemia [46]. One study described miR-29 to suppress PCa cell migration and invasion in vitro [47]. Another study found miR-29 family expression to be mediated by androgen receptor signaling [48]. Both miRNAs have not yet been described as biomarkers in PCa. At both the serum and the tissue level, miR-29 family members have mostly been described as inversely correlated with aggressiveness in various cancers, yet no data for PCa are available [49]. No studies have specifically analyzed miR-10 family expression in PCa tissue, and in other tumor types, data are also scarce. In a recent study, miR-99a-5p expression correlated with overall survival of lung adenocarcinoma in a reanalysis of TCGA (The Cancer Genome Atlas project) data [50]. The biological reason why two miRNAs, mostly described as tumor-suppressive, are enriched in EVs shed to the blood stream remains speculative. Yet, recently, a selective release of tumor-suppressive miRNAs miR-10a-5p, miR-193a-3p, miR-200b-5p, and miR-222-3p via exosomes was described in a colorectal cancer mouse model, while miRNAs with ongogenic effects (e.g., miR-196a/b, miR-181d-5p, and miR-155-5p) were retained in the tumor cells, in total resulting in enhanced tumor cell growth and tumor progression [51]. Blocking of this process resulted in intracellular accumulation of tumor-suppressive miRNAs and reduced tumor growth. Patients with more advanced colorectal cancer showed higher levels of circulating tumor-suppressive exosomal miR-193a-3p.

From the technical point of view, we utilized and validated a streamlined approach to reliably sequence and analyze the ncRNA content of PCa EVs in comparison with their parental cells. The isolation of cell culture EVs was done with a multi-step ultracentrifugation protocol including purification on a sucrose cushion to increase sample purity for the biomarker discovery part of the project [52]. In contrast to this, the isolation of patient EVs was done with a fast and ready-to-use filter system; as for the analysis of clinical samples, fast and robust techniques are needed. With both techniques, the isolated particles showed a typical size distribution and protein marker expression. However, we cannot exclude any co-isolation with common contaminants that might be present in the preparations. 

To accurately profile the EV content, potent techniques are needed. NGS techniques have replaced expression microarrays as workhorses for many applications of RNA-profiling. NGS has greater sensitivity and accuracy than microarrays and, as it does not rely on probes, produces data on single nucleotide resolution. Furthermore, detection is not limited to a defined probe set. NGS reference databases are more frequently updated than new microarray probe sets, allowing researchers to keep pace with the rapidly growing scientific knowledge.

Using our modified protocol, it was possible to generate libraries sufficient for sequencing from only 20 ng of RNA input without preamplification. In contrast, most standard RNA-sequencing protocols for ncRNA typically require an input between 100 and 1000 ng of RNA per sample or a further preamplification step [53]. A customized pipeline consisting of open access tools was used for read curation, filtering, and trimming. A compilation of several large public reference databases, customized for ncRNA identification, was used for mapping of identified sequences. As individual databases can be easily removed from or included in this compilation, this approach provides greater flexibility compared with the mapping against a whole reference genome. Mapping was done using a well-accepted Bowtie algorithm. DESeq2, which was used for differential expression analysis, is one of the most widely used state-of-the-art, variance-based algorithms for differential expression analysis in NGS data [54].

On this basis, our analyses revealed a distinct RNA composition of PC3 cells and EVs. Especially, miRNA, rRNA, and tRNA were enriched in EVs, whereas other studies showed differing results regarding the RNA composition of EVs. Especially, the amount of rRNA varies substantially. In some reports, rRNA is by far the biggest RNA class [25,55], while in other studies, rRNAs constitute only several percent of the RNA content [56]. This might either be because of biological reasons like different starting materials (cell culture supernatant, biofluids) or tissue specificity, or because of technical aspects of cell culture, EV isolation, or NGS. The low amount of mapped rRNA sequences in our study is most likely a result of our RNA extraction protocol, omitting fragments larger than 200 nt. Furthermore, our stringent mapping protocol excluded all reads not covering at least 50% of the sequence to which they mapped. For this reason, the results focus on short ncRNA classes. Among these, snoRNA were found to be predominant in cells. Because smaller EVs in particular, like exosomes, originate from early endosomes and are subsequently loaded with components mainly from the cellular cytoplasm, an underrepresentation of nuclear or mitochondrial RNAs agrees with their biogenesis and can be seen as an indicator of sample quality.

In summary, we used a streamlined protocol for NGS analyses of the ncRNA content of low input EV samples. By profiling PC3 prostate cancer EVs in comparison with their parental cells, we were able to gain insight into both the differential distribution of ncRNA classes and specific miRNAs. Using qRT-PCR, we could technically validate our sequencing results and simultaneously identify members of the miR-10 family to be enriched in PC3 EVs. The results from other benign and malignant prostate cell lines were partially overlapping with the results from PC3. Retesting in patient plasma EVs revealed miR-10a-5p and miR-29b-3p to be significantly overexpressed in patients with PCa, and thus might serve as potential biomarkers, whose clinical value should now be examined in larger cohorts.

## 4. Materials and Methods

### 4.1. Cell Culture and EV Isolation

The metastatic PCa cell lines PC3, LNCaP, and DU145, as well as benign prostatic PNT1a and BPH1 cells (all from LGC Standards, Wesel, Germany), were expanded under standard cell culture conditions in RPMI 1640 (Life Technologies, Carlsbad, CA, USA) supplemented with 10% bovine FCS (fetal calf serum, Biochrom, Berlin, Germany). For BPH1 cells, 20 ng/mL of dihydrotestosterone was also supplemented. Cell line authentication was performed by Multiplexion (Heidelberg, Germany). Then, 60 × 10^6^ (PC3 and LNCaP) or 120 × 10^6^ (PNT1a) were seeded into CELLine AD 1000 cell culture bioreactors (Integra Bioscience, Zizers, Switzerland). After three passages, standard medium was replaced by EV production medium. To generate EV production medium, FCS was diluted to 40% in RPMI 1640 and ultracentrifuged at 4 °C overnight at 110,000 g. The supernatant was then filtered through a 220 nm filter, further diluted to 5% FCS with RPMI 1640, and 1% of glutamate (Life Technologies) was added. After two further splitting passages, supernatant from the cell-containing compartments of bioreactors or standard cell culture flasks was extracted and subjected to EV isolation. At the same time point, cells were extracted, washed twice with sterile PBS (Life Technologies), and frozen at −80 °C. DU145 and BPH1 cells did not sufficiently grow in cell culture bioreactors and were thus expanded in standard cell culture flasks. At a density of 40–50%, standard cell culture medium was replaced by EV production medium, which was then harvested for EV isolation after 72 h. After harvesting EVs, the producing cells were checked for viability using the LUNA automated cell counter (logos biosystems, Villeneuve d’Ascq, France).

Cell culture supernatant was subjected to a well-accepted sequential centrifugation and ultracentrifugation protocol, including purification on a cushion of 20% sucrose in deuteriumoxide (Sigma Aldrich, St. Louis, MO, USA), representing the current gold standard for EV isolation [52,57]. Owing to the larger sample, supernatant from standard cell culture flasks was prepared in several vials in parallel and pellets were pooled after the first ultracentrifugation concentration step. The dissolved EV pellet was either used immediately for downstream analysis or stored at –80 °C.

To ensure quality control and to prevent potential contamination with bovine components, control samples consisting of a similar amount (120 mL) of the same EV-depleted FCS-containing EV-production medium were subjected to the same isolation process after incubation for 72 h in empty cell culture flasks.

### 4.2. Patient Samples

Blood plasma samples of patients with PCa (*n* = 18, age: median: 65 years, range: 48–72, PSA: median: 7.2 ng/mL, range: 3.37–94.7 ng/mL) and control patients with BPH (*n* = 7, age: median: 66 years, range: 59–80, PSA: median: 3.4 ng/mL, range: 0.7–10.0 ng/mL) were collected in the Department of Urology and Urosurgery of the University Medical Center Mannheim, Germany, in standard 9 mL K+/EDTA-tubes (Sarstedt). Blood samples were processed within 6 h by centrifugation at 2000× *g* for 10 min at 4 °C. The plasma was subsequently aliquoted and stored at −80 °C. The study is in accordance with the institutional review board (ethics approval 2015-549N-MA).

For EV isolation, 1 mL plasma was thawed at room temperature. Afterwards, samples were centrifuged at 20,000× *g* for 30 min at 4 °C. EVs were then isolated from the supernatant with the ExoRNeasy midi kit (Qiagen, Hilden, Germany) according to the instructions of the manufacturer. EVs were not directly lysed for RNA isolation, but were eluted from the isolation columns using Qiagen buffer XE to allow EV quality control prior to RNA isolation. Routine formalin-fixed paraffin-embedded (FFPE) tissue samples of the same patients, obtained during radical prostatectomy due to PCa or TUR-P (transurethral resection of the prostate) due to BPH, were used for tissue-based analyses, if available (*n* = 14 patients with PCa and *n* = 6 controls).

### 4.3. Sample Quality Control

To assess sample quality, 2 μL of PC3 cell culture EVs was diluted in sterile filtered PBS (dilutions were typically in the range between 1:2000 and 1:10,000) and visualized using the LM10 NTA device (Malvern Instruments, Malvern, UK). PC3 sample for the NGS analysis was measured six times for 45 s (Screen Gain 1.0, camera level 15). NTA measurements of cell line EVs for validation experiments, media control samples, and patient EVs were done with a ZetaView instrument (Particle Metrix, Meerbusch, Germany) at dilutions of 1:10,000 and 1:100,000 for cell culture samples and 1:2000 for patient samples (sensitivity 80, shutter 100). The data were analyzed with the FlowJo 10 Software (FlowJo LLC, Ashland, OR, USA).

A total of 5 μL of EVs from the used cell lines, media control samples, and patient EVs were placed on 100 mesh formvar-coated copper electron microscopy grids for 5 min. The grids were then washed three times with distilled water. Negative stain was performed with 3% uranylacetate for 3 min. Grids were subsequently air-dried and visualized with an EM900 transmission electron microscope (Zeiss, Oberkochen, Germany) at 30 k, 50 k, and 85 k magnification.

The protein content of EVs, cell lysates, and media control samples was determined using the BCA assay (Thermo Fisher Scientific. Waltham, MA, USA) and the Bradford assay (Bio-Rad, Munich, Germany) according to the manufacturer’s protocol.

For immunoblotting, sample preparation was performed with both reducing and non-reducing Laemmli buffer. Then, 2 μg (PC3) or 10 μg (BPH1, PNT1a, DU145, LNCaP) of proteins from EV and cell lysates were loaded on 4–12% SDS gels for electrophoresis, followed by transfer to the PVDF membrane (PC3) or nitrocellulose membrane (BPH1, DU145, LNCaP). Primary antibodies used were polyclonal rabbit anti-Calnexin (1:1000), mouse monoclonal anti-HSC70 (1:500 clone W27) (both from Santa Cruz, Heidelberg, Germany), mouse monoclonal anti-CD9 (1:500 clone MEM-61, Immunotools, Friesoythe, Germany) and rabbit polyclonal anti-TSG101 (1:500 Sigma Aldrich, St. Louis, MO, USA), mouse anti-CD63 (1:250, clone MX-49.129.5, Santa Cruz, Heidelberg, Germany), mouse anti-CD9 (1:200, clone C4, Santa Cruz, Heidelberg, Germany), mouse anti-β-Actin (1:2000, clone C4, MP Biomedicals, Santa Ana, CA, USA), rabbit anti-Alix (1:1000, clone EPR15314-33, Abcam, Cambridge, UK), mouse anti-TSG101 (1:500, clone 4A10, Abcam, Cambridge, UK), and rabbit anti-Syntenin (1:5000, clone EPR8102, Abcam, Cambridge, UK).

HRP-conjugated goat anti-mouse or anti-rabbit (both from Jackson ImmunoResearch, Westgrove, PA, USA) or sheep anti-mouse (GE Healthcare, Munich, Germany) and goat anti-rabbit (Promega, Mannheim, Germany) were used as secondary antibodies.

For the flow cytometry analysis, 0.5 × 10^9^ particles according to the ZetaView results from cell line and patient EVs and media control samples were coupled to magnetic beads (EV-Human CD9 Flow Detection Reagent, Invitrogen, Germany) and incubated overnight at 4 °C. A blocking step was performed with FcR antibody (BD biosciences, Heidelberg, Germany) for 15 min at room temperature. After washing the beads with 0.1% BSA/PBS, the primary antibodies against CD9 (clone M-L13, PE-conjugated), CD63 (clone H5C6, PE-Cy7-conjugated), and CD81 (clone JS-81, FITC-conjugated; all three from BD biosciences) were added. The mixture was incubated for 30 min at room temperature and washed three times. Furthermore, a negative control was used, which was not stained with the CD9, CD63, or CD81 antibodies. The flow cytometry analysis was performed with BD FACSCanto II (BD biosciences) and analyzed with FlowJo 10 Software.

### 4.4. RNA Extraction from EVs and NGS Library Preparation

RNA from cells, EVs, and media controls was extracted using the miRNeasy micro Kit (Qiagen, Hilden, Germany) following the instructions of the manufacturer for the separate extraction of RNA molecules up to 200 nt. RNA was eluted in 14 μL of RNAse-free H_2_O (Life Technologies). RNA concentration was measured using RNA 6000 Pico and small RNA chip on an Agilent 2100 Bioanalyzer (Agilent Technologies, Santa Clara, CA, USA).

For NGS library preparation with NEBNext^®^ Multiplex Small RNA Library Prep Set for Illumina^®^ (New England Biolabs, Ipswich, MA, USA), 20 ng of RNA of each sample, according to the small RNA chip results, was used. For the library preparation of two biological replicates of PC3 cells and EVs, each was performed as recommended by the manufacturer with minor modifications. Sequencing adapters and reverse transcription primers were diluted 1:2, owing to the low sample amount, and the PCR-amplification was extended to 15 cycles. To prevent loss of material, and because of the size-restrictive RNA isolation, a size selection of the raw library was omitted. The final libraries were validated on an Agilent 2100 Bioanalyzer using a high sensitivity DNA chip. To prevent a distortion of library concentrations by primer and adapter dimers, the actual library concentration was determined using the calculated bioanalyzer concentration of the average region size, omitting primer and adapter dimers (size range between 130 and 300 bp).

### 4.5. Sequencing and Data Analysis

Libraries were normalized to 10 nM, using Bioanalyzer concentrations and average region size; pooled in equimolar amounts; and clustered on the cBot (Illumina, San Diego, CA, USA) with a concentration of 10 pM spiked with 1% PhiX control v3 according the manufacturer’s instructions. Then, 51 bp single read sequencing on an Illumina HiSeq 2000 v3 platform (Illumina) was performed according to the manufacturer’s protocol.

The resulting sequencing reads were subjected to a customized script to evaluate, filter, and trim reads. During this curation step, Cutadapt was used [58] to clip the adaptor sequence from the 3’ end of the reads, FastX [59] was used for filtering of artifacts and for deleting reads with bad quality, and HomerTools [60] was used to trim the poly-A tail from the 3’ end of the reads.

Next, the cleaned fastq files were mapped against a custom-built database of unique human ncRNA sequences compiled from the databases piRNA-Cluster [61], Ensembl [62], RFAM [63], and mirBase [64]. Details on database versions and annotated RNA classes are shown in Table S3.

The mapping was done using Bowtie, allowing for 0 mismatches. Further restrictions were used as well; that is, the read sequences were required to cover at least half of a small ncRNA sequence in the database in order to be counted as a match. At the same time, the read sequence could only be a maximum of 1.5-times the size of an annotated ncRNA sequence.

Custom R/Bioconductor-scripts [65] were used for the analysis of the ncRNA expression and the plotting of results. Inter-replicate correlation was performed using Pearson correlation, *determining r* as a measure of correlation. The Bioconductor Package DESeq2 was used for determining differential expression of single RNAs [54]. All *p*-values were adjusted using the Benjamini–Hochberg method, also known as the false discovery rate (FDR).

### 4.6. qRT-PCR Analyses of EVs from Cell Lines and Blood Plasma and of Tissue Samples

For validation of miRNA expression, validated qRT-PCR assays were used. According to the sequencing results, miRNAs enriched in PC3 cells (miR-29b-3p, miR-3607-3p, miR-5701) or EVs (miR-10a-5p, miR-99b-5p, miR-125a-5p), or those with a stable expression (miR-20a-5p, miR-28-5p), were selected. The same miRNAs were also tested in EVs derived from patient plasma.

RNA extracted from three independent biological replicates of EVs and cells of PC3, LNCaP, DU145, BPH1, and PNT1a cell lines, as well as of patient EVs, was transcribed into cDNA using target specific stem-loop primers of commercially available TaqMan Assays (Thermo Fisher Scientific). For each cell culture derived sample, 500 pg of RNA was used as input for sequence-specific cDNA synthesis. For patient EV samples, 50 pg of RNA were used owing to the lower sample amount.

Hematoxylin and eosin stained slides of FFPE tissue samples were reviewed by an experienced uropathologist (S.P.). Tumor-bearing regions were marked and dissected from subsequent unstained 10 μm tissue slides. The dissected material was subjected to RNA extraction using the bead-based XTRACT Kit (STRATIFYER, Cologne, Germany) according to the instruction of the manufacturer. From patients with BPH, whole 10 μm tissue slides were used. Target-specific cDNA transcription was also performed with the same protocol as for EVs with 10 ng of RNA as input for each reaction. 

Again, using established TaqMan assays (Thermo Fisher Scientific), qRT-PCR analyses were conducted to validate the expression of miRNAs. In cell line samples, the expression of each target RNA was determined in triplicates in each biological replicate. Expression of differentially enriched miRNAs relative to miR-20a-5p and miR-28-5p was calculated using the 2^−ΔΔCT^-method for cell line samples. In each plasma and tissue sample, target RNA expression was measured in duplicates. The relative expression was calculated using the 40^−ΔCt^ method [66,67,68]. Statistical analyses were performed with Prism 7 (Graphpad Software Inc., LA Jolla, CA, USA) using the two-sided paired t-test or the non-parametric Mann–Whitney test where appropriate. *p*-values < 0.05 were deemed statistically significant.

## 5. Conclusions

After identification by low input NGS analysis and retesting with specific qRT-PCR assays, EV-associated miR-10a-5p and miR-29b-3p are proposed as PCa detection markers. Further investigation in larger cohorts and prospective studies are needed to validate these findings.

## Figures and Tables

**Table 1:** Processed reads mappable to different ncRNA classes in each replicate of prostate cancer (PC)3 cells and extracellular vesicles (EVs).

Sample	snoRNA (%)	miRNA (%)	tRNA (%)	rRNA (%)	piRNA (%)	Mt_tRNA (%)	snRNA (%)
cell 1	3,540,368 (56.40)	2,374,035 (37.82)	124,497 (1.98)	148,002 (2.36)	40,914 (0.65)	48,378 (0.77)	1511 (0.02)
cell 2	4,708,633 (64.01)	2,040,209 (27.73)	411,819 (5.60)	78,915 (1.07)	40,408 (0.55)	73,719 (1.00)	2722 (0.04)
**average**	**4,124,500** **(60.50)**	**2,207,122** **(32.38)**	**268,158** **(3.93)**	**113,458.5** **(1.66)**	**40,661** **(0.60)**	**61,048.5** **(0.90)**	**2116.5** **(0.03)**
EV 1	138,716 (6.26)	1,514,573 (68.39)	173,397 (7.83)	350,202 (15.81)	31,573 (1.43)	1649 (0.07)	4459 (0.20)
EV 2	132,583 (8.35)	1,004,736 (63.27)	202,431 (12.75)	203,911 (12.84)	40,494 (2.55)	1279 (0.08)	2467 (0.16)
**average**	**135,649.5** **(7.13)**	**1,259,654** **(66.25)**	**187,914** **(9.88)**	**277,056.5** **(14.57)**	**36,033.5** **(1.9)**	**1464** **(0.08)**	**3463** **(0.18)**

**Table 2:** Top 15 miRNAs enriched in EVs. FDR, false discovery rate.

miRNA	log^2^ Fold Change	*p*-Value	FDR-Adjusted *p*-Value
hsa-miR-3605-3p	6.06	1.53 × 10^−32^	3.95 × 10^−31^
hsa-let-7d-3p	5.09	4.32 × 10^−39^	1.34 × 10^−37^
hsa-miR-146a-5p	4.20	4.22 × 10^−18^	3.06 × 10^−17^
hsa-miR-937-3p	3.72	3.94 × 10^−16^	2.38 × 10^−15^
hsa-let-7b-3p	3.67	2.65 × 10^−19^	2.16 × 10^−18^
hsa-miR-99b-5p	3.67	5.12 × 10^−27^	7.93 × 10^−26^
hsa-miR-92b-3p	3.66	1.32 × 10^−32^	3.61 × 10^−31^
hsa-miR-125a-5p	3.59	2.35 × 10^−19^	1.95 × 10^−18^
hsa-miR-873-3p	3.50	1.14 × 10^−17^	7.91 × 10^−17^
hsa-miR-320b	3.49	2.35 × 10^−24^	3.12 × 10^−23^
hsa-miR-625-3p	3.48	1.83 × 10^−22^	2.13 × 10^−21^
hsa-miR-320c	3.29	3.23 × 10^−19^	2.54 × 10^−18^
hsa-miR-1247-5p	3.24	1.54 × 10^−16^	9.97 × 10^−16^
hsa-miR-2110	3.15	6.22 × 10^−11^	2.39 × 10^−10^
hsa-miR-105-5p	3.09	8.05 × 10^−16^	4.80 × 10^−15^

**Table 3:** Top 15 miRNAs depleted in EVs.

miRNA	log^2^ Fold Change	*p*-Value	FDR-Adjusted *p*-Value
hsa-miR-3607-3p	−5.07	5.59 × 10^−11^	2.19 × 10^−10^
hsa-miR-5701	−4.16	1.13 × 10^−08^	3.59 × 10^−8^
hsa-miR-193a-3p	−3.60	8.39 × 10^−07^	2.23 × 10^−6^
hsa-miR-1246	−2.54	2.26 × 10^−16^	1.42 × 10^−15^
hsa-miR-582-5p	−2.50	3.81 × 10^−06^	9.22 × 10^−6^
hsa-miR-19b-3p	−2.49	7.81 × 10^−11^	2.93 × 10^−10^
hsa-miR-561-5p	−2.36	1.74 × 10^−06^	4.37 × 10^−6^
hsa-miR-26a-2-3p	−2.31	9.51 × 10^−07^	2.48 × 10^−6^
hsa-miR-19a-3p	−2.20	2.00 × 10^−07^	5.65 × 10^−7^
hsa-miR-29b-3p	−1.97	7.07 × 10^−10^	2.42 × 10^−9^
hsa-miR-101-3p	−1.97	1.51 × 10^−08^	4.75 × 10^−8^
hsa-miR-29c-3p	−1.92	3.80 × 10^−06^	9.22 × 10^−6^
hsa-miR-32-5p	−1.73	8.96 × 10^−06^	2.09 × 10^−5^
hsa-miR-17-3p	−1.69	8.17 × 10^−05^	1.73 × 10^−4^
hsa-miR-590-3p	−1.65	2.16 × 10^−05^	4.25 × 10^−4^

**Table 4:** Ten members of miR-10 family were enriched in EVs.

miRNA	log^2^ Fold Change	*p*-Value	FDR-Adjusted *p*-Value
hsa-miR-99b-5p	3.67	5.12 × 10^−27^	7.93 × 10^−26^
hsa-miR-125a-5p	3.59	2.35 × 10^−19^	1.95 × 10^−18^
hsa-miR-125b-5p	2.48	2.75 × 10^−09^	9.01 × 10^−09^
hsa-miR-125a-3p	2.32	3.59 × 10^−07^	9.89 × 10^−07^
hsa-miR-99a-5p	2.03	4.08 × 10^−09^	1.32 × 10^−08^
hsa-miR-10a-5p	1.92	1.37 × 10^−10^	5.06 × 10^−10^
hsa-miR-99b-3p	1.87	7.41 × 10-^06^	1.76 × 10^−05^
hsa-miR-10a-3p	1.70	1.25 × 10^−04^	2.57 × 10^−04^
hsa-miR-125b-1-3p	1.28	1.53 × 10^−04^	3.09 × 10^−04^
hsa-miR-10b-5p	1.03	3.10 × 10^−02^	4.71 × 10^−02^

**Table 5:** Though particles from control samples showed a similar size distribution in nanoparticle tracking analysis (NTA), the particle concentration was lower by at least 3 log doses compared with cell culture EVs. BPH, benign prostatic hyperplasia.

Cell Line	Mean Particle Concentration ± SD	Mean Diameter of Particles ± SD
PC3	1.85 × 10^12^/mL ± 1.20 × 10^12^/mL	179.5 nm ± 29.9 nm
DU145	4.15 × 10^11^/mL ± 4.20 × 10^11^/mL	185.6 nm ± 7.7 nm
LNCaP	2.54 × 10^11^/mL ± 1.14 × 10^11^/mL	150.7 nm ± 21.0 nm
BPH1	1.67 × 10^11^/mL ± 1.08 × 10^11^/mL	210.9 nm ± 10.4 nm
PNT1a	1.47 × 10^11^/mL ± 1.23 × 10^11^/mL	180.6 nm ± 31.8 nm
medium control	1.04 × 10^9^/mL ± 3.11 × 10^7^/mL	156.2 nm ± 26.4 nm

**Table 6:** Characteristics of the analyzed patients (* Gleason score according to biopsy results).

Parameter	PCa Patients (*n* = 18)	BPH Controls (*n* = 7)
Median age (years)	65 (48–72)	66 (59–80)
pT stage		
2c	14	
3a	2	
3b	1	
no surgery	1	
Gleason Score		
3 + 3	2	
3 + 4	7	
4 + 3	6	
4 + 4	1 *	
4 + 5	1	
5 + 4	1	
D’Amico risk group		
low	5	
intermediate	7	
high	6	
gland volume (mL)	33 (15–59)	44 (20–120)
Median serum PSA level (ng/mL)	7.2 (3.3–94.7)	3.4 (0.7–10.0)

PSA: prostate specific antigen.

## Supplementary Materials

The following are available online at https://www.mdpi.com/2072-6694/12/1/43/s1, Figure S1: Complete WB results referring to Figure 1d, Figure S2: Bioanalyzer profiles of isolated RNA, Figure S3: Correlation analysis of PC3 sequencing samples, Figure S4: MA plot showing over- and underexpressed RNAs between PC3 cells and EVs, Figures S5–S9: WB analysis of cellular and EV protein extracts and medium controls, Figure S10: Comparison of miRNA expression between malignant and benign parental cells, Figure S11: miRNA expression in FFPE samples of PCa patients compared with BPH controls, Table S1: All identified RNAs in PC3 cells and EVs, Table S2: Overview of all identified miRNAs, Table S3: Database versions and annotated ncRNA classes.
