# Supplementary material for: miR-10a-5p and miR-29b-3p as Extracellular Vesicle-Associated Prostate Cancer Detection Markers
Source: Cancers (Basel). 2019 Dec 21;12(1):43. doi: 10.3390/cancers12010043 (PMC7017198; doi:10.3390/cancers12010043)
Supplement: Supplementary file 1 [file cancers-12-00043-s001.zip › cancers-672708-suppl-XML/cancers-672708-Figures S1-11 and Table S3.docx]

Supplementary Materials: miR-10a-5p and miR-29b-3p as Extracellular Vesicle-Associated Prostate Cancer Detection Markers

Thomas S. Worst, Christopher Previti, Katja Nitschke, Nicolle Diessl, Julia C. Gross, Lena Hoffmann, Lisa Frey, Vanessa Thomas, Christoph Kahlert, Karen Bieback, Adriana Torres Crigna, Fabia Fricke, Stefan Porubsky, Niklas Westhoff, Jost von Hardenberg, Philipp Nuhn, Philipp Erben, Maurice S. Michel and Michael Boutros

**Figure S1.** Complete WB results referring to Figure 1d. Membranes were cut before antibody staining to allow multiple staining of a single gel without stripping off antibodies. Therefore, no complete membranes can be shown for this experiment.


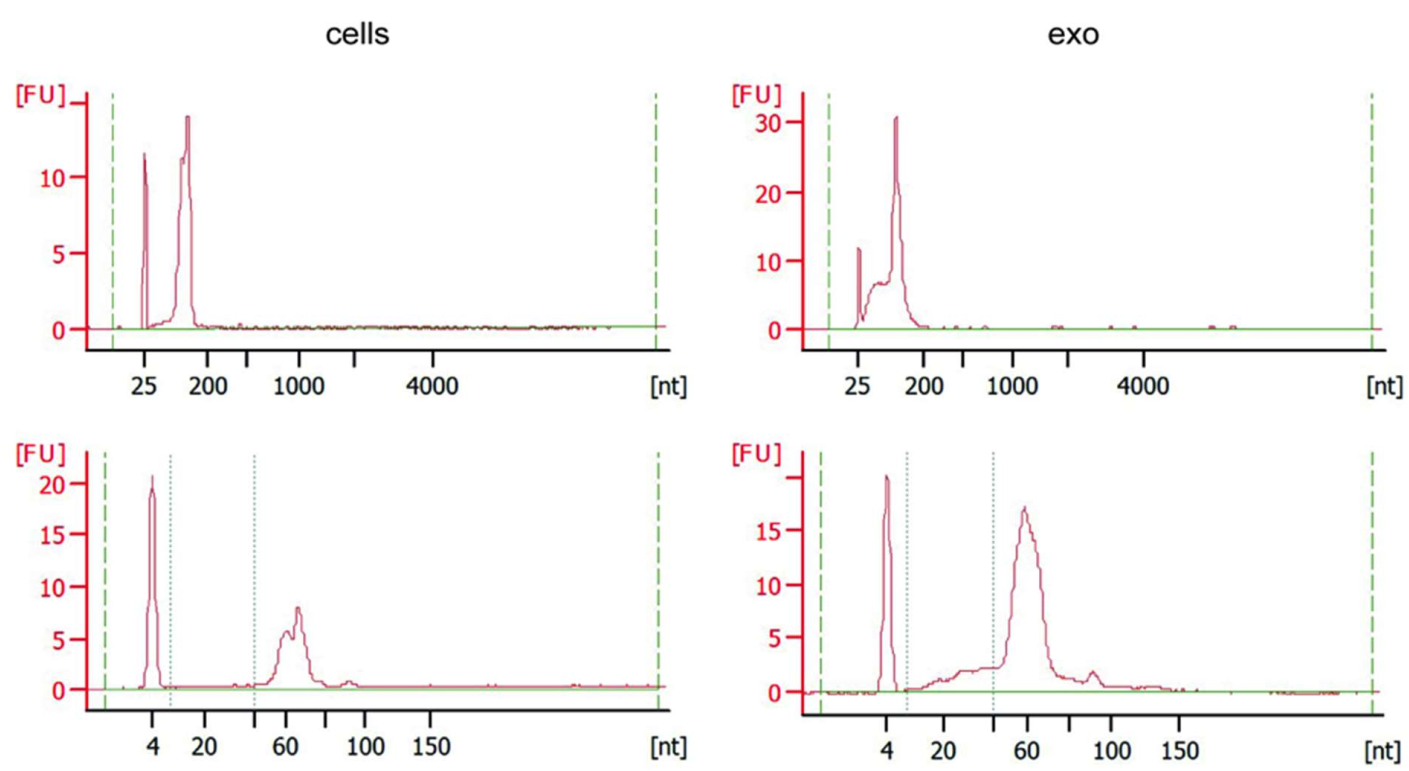


**Figure S2.** Bioanalyzer profiles of isolated RNA: Both on the RNA Pico (upper) and on the small RNA chip (lower), PC3 EVs (right column) showed a higher proportion of shorter RNA-fragments compared to PC3 cells (left column).

**Figure S3.** Correlation analysis of PC3 sequencing samples.

**Figure S4.** MA plot showing over- and underexpressed RNAs between PC3 cell and EVs.

**Figure S5.** WB analysis for β-Actin of cellular and EV protein extracts and medium controls.

**Figure S6.** WB analysis for TSG101 of cellular and EV protein extracts and medium controls.

**Figure S7.** WB analysis for Syntenin of cellular and EV protein extracts and medium controls.

**Figure S8.** WB analysis for ALIX of cellular and EV protein extracts and medium controls.

**Figure S9.** WB analysis for CD9 and CD63 of cellular and EV protein extracts and medium controls.

**Figure S10.** When comparing miRNA expression between malignant and benign parental cells only for miR-99b-5p an overexpression in malignant cells was found. Absolute expression showed a strong variation between the different cell lines. (* *p* < 0.05).

**Figure S11.** No significant differences in expression of target miRNAs in tissue samples of patients with BPH and PCa were observed.

**Table S3.** Databases used as reference for read mapping.

| **Database** | **ncRNA Classes** | **Version** |
| --- | --- | --- |
| piRNA-Cluster | piRNA | 2012 |
| Ensembl | snoRNA, rRNA, tRNA, snRNA, miRNA,  lincRNA, miscRNA | 79 |
| RFAM | snoRNA, rRNA, tRNA, snRNA, miRNA | 11 |
| mirBase | miRNA | 21 |

| 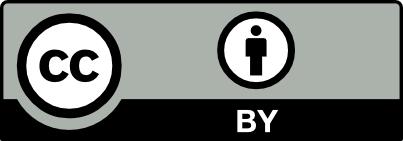 | © 2020 by the authors. Licensee MDPI, Basel, Switzerland. This article is an open access article distributed under the terms and conditions of the Creative Commons Attribution (CC BY) license (http://creativecommons.org/licenses/by/4.0/). |
| --- | --- |
