# Supplementary material for: miR-10a-5p and miR-29b-3p as Extracellular Vesicle-Associated Prostate Cancer Detection Markers
Source: Cancers (Basel). 2019 Dec 21;12(1):43. doi: 10.3390/cancers12010043 (PMC7017198; doi:10.3390/cancers12010043)
Supplement: Supplementary file 1 [file cancers-12-00043-s001.zip › cancers-672708-suppl-XML/cancers-672708-Table S1.docx]

| **ncRNA_ID** | **Source_DB** | **Source_ID** | **Organism** | **ncRNA_type** | **ncRNA_Symbol** | **Associated.Gene.Name** | **ncRNA_Sequence** | **PC3_Cells_1** | **PC3_Cells_2** | **PC3_EV_1** | **PC3_EV_2** | **baseMean** | **log2FoldChange** | **lfcSE** | **stat** | **p-value** | **FDR adj p-value** |
| --- | --- | --- | --- | --- | --- | --- | --- | --- | --- | --- | --- | --- | --- | --- | --- | --- | --- |
| HUS00066951 | GtRNA\|GtRNA | chr6\|chr6 | Homo_sapiens | tRNA | trna152-ValCAC | trna152-ValCAC | GCTTCTGTAGTGTAGTGGTTATCACGTTCGCCTCACACGCGAAAGGTCCCCGGTTCGAAACCGGGCAGAAGCA | 271 | 226 | 2751 | 2273 | 2200,499507 | 4,785410835 | 0,276601216 | 17,30075849 | 4,64236E-67 | 2,1587E-64 |
| HUS00137382 | Ensembl | ENST00000365659 | Homo_sapiens | snoRNA | ENSG00000202529 | SNORD18B | TCAAAATGATGAGATTCCACTTAATTGGTCCGTGTTTCTGAAACACATGATATTTGTGGAAATTCTGACTTG | 19237 | 13077 | 121 | 102 | 4877,370779 | -5,556084045 | 0,335969694 | -16,53745603 | 1,97175E-61 | 4,58432E-59 |
| HUS00366660 | Ensembl | ENST00000459174 | Homo_sapiens | snoRNA | ENSG00000238578 | SNORD4A | GGTGCAGATGATGACACTGTAAAGCGACCAAAGTCTGAACAAAGTGATTGGTACCTCGTTGTCTGATGCACC | 66125 | 39807 | 193 | 203 | 15808,09136 | -6,352577933 | 0,395485463 | -16,06273433 | 4,65567E-58 | 7,21629E-56 |
| HUS00170101 | Ensembl | ENST00000362704 | Homo_sapiens | snoRNA | ENSG00000199574 | SNORD18C | TTGTTATGATGAGATTCCACTTAAGGTCCGTGTTTCTGAAACAAATGATTTTGTGGAAGTTCTGATTTA | 10510 | 10096 | 104 | 110 | 3165,633187 | -4,982247895 | 0,317174257 | -15,70823542 | 1,32825E-55 | 1,54409E-53 |
| HUS00298663 | Rfam\|Ensembl | RF00005\|ENST00000387421 | Homo_sapiens | tRNA | ENSG00000210156 | MT-TK | CACTGTAAAGCTAACTTAGCATTAACCTTTTAAGTTAAAGATTAAGAGAACCAACACCTCTTTACAGTGA | 16349 | 11581 | 175 | 143 | 4272,236875 | -4,86214806 | 0,315401301 | -15,41575144 | 1,2827E-53 | 1,19291E-51 |
| HUS00104764 | Rfam\|Ensembl | RF01191\|ENST00000458838 | Homo_sapiens | snoRNA | SNORD121A | SNORD121A | CTGGAAAAGACAATGATGTTTTATTTCCAAGCACATATCTGAGTTGTATGTGTGGACAGCACTGAGACTGAGTCTTTCCAC | 6479 | 6702 | 106 | 71 | 2043,338253 | -4,659348093 | 0,304938293 | -15,27964246 | 1,04513E-52 | 8,09972E-51 |
| HUS00164148 | GtRNA\|GtRNA | chr6\|chr6 | Homo_sapiens | tRNA | trna144-AspGTC | trna144-AspGTC | TCCTCGTTAGTATAGTGGTGAGTGTCCCCGTCTGTCACGCGGGAGACCGGGGTTCGATTCCCCGACGGGGAG | 86 | 107 | 6523 | 2529 | 3692,011584 | 6,747524925 | 0,447442297 | 15,0802125 | 2,18564E-51 | 1,45189E-49 |
| HUS00262718 | Ensembl | ENST00000410413 | Homo_sapiens | snoRNA | ENSG00000222345 | SNORD19 | AAGAAATGAAGAAACTAAAATTGGTCTTAGTATTGAAGTGAAGACACTGAGATCCAACTCTGATCTTGCCCTAAAC | 69061 | 99822 | 854 | 650 | 26054,57974 | -5,220118653 | 0,347402902 | -15,02612278 | 4,95152E-51 | 2,87807E-49 |
| HUS00091828 | Ensembl | ENST00000583861 | Homo_sapiens | snoRNA | ENSG00000263764 | SNORD43 | CACAGATGATGAACTTATTGACGGGCGGACAGAAACTGTGTGCTGATTGTCACGTTCTGATTTG | 184785 | 235142 | 2608 | 2466 | 65199,69813 | -4,776863294 | 0,327236379 | -14,59759243 | 2,90939E-48 | 1,50319E-46 |
| HUS00049131 | Ensembl | ENST00000390994 | Homo_sapiens | snoRNA | ENSG00000212296 | SNORD72 | AGCTTATCAGTGATGTTGTAAAAATAAATGTCTGAACATATGAATGCAGTATTGATTTCAGCATTTAACTGAGATAAGCG | 2855 | 2391 | 25 | 20 | 799,0947371 | -5,243133377 | 0,36017173 | -14,55731513 | 5,24787E-48 | 2,44026E-46 |
| HUS00185161 | Ensembl | ENST00000364995 | Homo_sapiens | snoRNA | ENSG00000275072 | SNORD50B | AATCAATGATGAAACCTATCCCGAAGCTGATAACCTGAAGAAAAATAAGTACGGATTCGGCTTCTGAGAT | 10406 | 10276 | 125 | 57 | 3161,204409 | -5,247025812 | 0,369560616 | -14,19801131 | 9,42492E-46 | 3,98417E-44 |
| HUS00327851 | GtRNA\|GtRNA\|GtRNA\|GtRNA | chr1\|chr1\|chr1\|chr1 | Homo_sapiens | tRNA | trna132-GlyCCC | trna132-GlyCCC | GCATTGGTGGTTCAGTGGTAGAATTCTCGCCTCCCACGCGGGAGACCCGGGTTCAATTCCCGGCCAATGCA | 227 | 243 | 1641 | 1381 | 1350,936796 | 4,129213825 | 0,291754145 | 14,1530597 | 1,78805E-45 | 6,9287E-44 |
| HUS00317161 | Ensembl | ENST00000387461 | Homo_sapiens | Mt_tRNA | ENSG00000210196 | MT-TP | CAGAGAATAGTTTAAATTAGAATCTTAGCTTTGGGTGCTAATGGTGGAGTTAAAGACTTTTTCTCTGA | 3844 | 6233 | 21 | 12 | 1534,233317 | -6,53122508 | 0,468311556 | -13,94632482 | 3,3124E-44 | 1,18482E-42 |
| HUS00227638 | PiRNA | DQ598159.1 | Homo_sapiens | piRNA | piR-36225 | piR-36225 | GGGGATGTAGCTCAGTGGTAGAGCGCATGCT | 1497 | 1327 | 15866 | 19628 | 15828,77982 | 5,072301981 | 0,377270729 | 13,44472708 | 3,30623E-41 | 1,09814E-39 |
| HUS00024472 | MiRBase | MIMAT0004484 | Homo_sapiens | mature_miRNA | hsa-let-7d-3p | hsa-let-7d-3p | CTATACGACCTGCTGCCTTTCT | 257 | 129 | 2756 | 2270 | 2183,794182 | 5,093968698 | 0,38946735 | 13,07932152 | 4,32281E-39 | 1,34007E-37 |
| HUS00176426 | GtRNA\|GtRNA | chr6\|chr6 | Homo_sapiens | tRNA | trna61-MetCAT | trna61-MetCAT | AGCAGAGTGGCGCAGCGGAAGCGTGCTGGGCCCATAACCCAGAGGTCGATGGATCTAAACCATCCTCTGCTA | 101 | 89 | 1581 | 727 | 971,1024171 | 4,928343695 | 0,393217119 | 12,53339049 | 4,90213E-36 | 1,42468E-34 |
| HUS00272786 | MiRBase | MIMAT0003218 | Homo_sapiens | mature_miRNA | hsa-miR-92b-3p | hsa-miR-92b-3p | TATTGCACTCGTCCCGGCCTCC | 287 | 342 | 1628 | 1299 | 1330,526916 | 3,655421725 | 0,307413761 | 11,89088513 | 1,32E-32 | 3,61059E-31 |
| HUS00035811 | MiRBase | MIMAT0017982 | Homo_sapiens | mature_miRNA | hsa-miR-3605-3p | hsa-miR-3605-3p | CCTCCGTGTTACCTGTCCTCTAG | 4 | 7 | 197 | 126 | 136,2181404 | 6,058592463 | 0,510044908 | 11,87854711 | 1,53004E-32 | 3,95259E-31 |
| HUS00015158 | Ensembl | ENST00000408612 | Homo_sapiens | snoRNA | ENSG00000221539 | SNORD99 | ACTGGTCCAGGATGAAACCTAATTTGAGTGGACATCCATGGATGAGAAATGCGGATATGGGACTGAGACCAGCT | 12908 | 14100 | 434 | 375 | 4383,670518 | -3,505805339 | 0,295263995 | -11,87346035 | 1,626E-32 | 3,97942E-31 |
| HUS00149999 | GtRNA\|GtRNA\|GtRNA\|GtRNA\|GtRNA\|GtRNA\|GtRNA\|GtRNA\|GtRNA\|GtRNA\|GtRNA\|GtRNA\|GtRNA\|GtRNA\|GtRNA\|GtRNA | chr1\|chr17\|chr6\|chr6\|chr6\|chr6\|chr6\|chr6\|chr1\|chr17\|chr6\|chr6\|chr6\|chr6\|chr6\|chr6 | Homo_sapiens | tRNA | trna32-MetCAT | trna32-MetCAT | AGCAGAGTGGCGCAGCGGAAGCGTGCTGGGCCCATAACCCAGAGGTCGATGGATCGAAACCATCCTCTGCTA | 120 | 105 | 2053 | 841 | 1208,092885 | 4,980564038 | 0,419936135 | 11,86028928 | 1,90316E-32 | 4,42486E-31 |
| HUS00192307 | GtRNA\|GtRNA | chr17\|chr17 | Homo_sapiens | tRNA | trna10-GlyTCC | trna10-GlyTCC | GCGTTGGTGGTATAGTGGTAAGCATAGCTGCCTTCCAAGCAGTTGACCCGGGTTCGATTCCCGGCCAACGCA | 21 | 30 | 321 | 195 | 221,9539929 | 4,680909766 | 0,399133654 | 11,72767497 | 9,19497E-32 | 2,03603E-30 |
| HUS00074073 | Ensembl | ENST00000384262 | Homo_sapiens | snoRNA | ENSG00000206989 | SNORD63 | GTGCAATGATGTATTTTATTCAACACATCATTCTGAAAGAACGTGTGGAAAACTAATGACTGAGCACA | 192218 | 201929 | 2829 | 3679 | 61744,42947 | -4,279548133 | 0,367492796 | -11,64525722 | 2,42589E-31 | 5,12744E-30 |
| HUS00264913 | Ensembl | ENST00000384027 | Homo_sapiens | snoRNA | ENSG00000206754 | SNORD101 | GTTTGAATGATGACTTTAATTGTCGGATACCCCTTCACTCCTTTTATGAGTGAAACATAAGAGTCTGACAAAC | 100715 | 190675 | 1675 | 793 | 45120,2664 | -5,253775589 | 0,460150209 | -11,41752299 | 3,41838E-30 | 6,91108E-29 |
| HUS00215392 | Ensembl | ENST00000629629 | Homo_sapiens | snoRNA | ENSG00000280554 | snoU18 | AAAATGATGAGATTCCACTTAATTGGTCCGTGTTTCTGAAACACATGATATTTGTGGAAATTCTGACTTGG | 2950 | 3216 | 34 | 49 | 958,7265828 | -4,547643941 | 0,406406595 | -11,18988716 | 4,57015E-29 | 8,85466E-28 |
| HUS00252691 | GtRNA\|GtRNA\|GtRNA\|GtRNA | chr11\|chrX\|chr11\|chrX | Homo_sapiens | tRNA | trna17-ValTAC | trna17-ValTAC | GGTTCCATAGTGTAGTGGTTATCACGTCTGCTTTACACGCAGAAGGTCCTGGGTTCGAGCCCCAGTGGAACCA | 100 | 80 | 563 | 333 | 398,2171688 | 3,724987531 | 0,337110107 | 11,04976521 | 2,19789E-28 | 4,08807E-27 |
| HUS00232062 | Ensembl | ENST00000364533 | Homo_sapiens | snoRNA | ENSG00000201403 | SNORD14B | TCACTGTGATGATGGTTTTCCAACATTCGCAGTTTCCACCAGAAAGGTTTTCCTTAGTGTTGGGTAAACCTTCCTTGGATGTCTGAGTGA | 3338 | 1992 | 24 | 6 | 798,5625228 | -5,731607885 | 0,520588593 | -11,00986069 | 3,42533E-28 | 6,12607E-27 |
| HUS00075706 | Ensembl | ENST00000459163 | Homo_sapiens | snoRNA | ENSG00000238942 | SNORD2 | AAGTGAAATGATGGCAATCATCTTTCGGGACTGACCTGAAATGAAGAGAATACTCATTGCTGATCACTT | 403280 | 548110 | 8420 | 7853 | 149957,6951 | -4,255976451 | 0,386844797 | -11,00176733 | 3,74715E-28 | 6,45342E-27 |
| HUS00074596 | Ensembl | ENST00000606577 | Homo_sapiens | snoRNA | ENSG00000272296 | SNORD96A | GGTCCTGGTGATGACAGATGGCATTGTCAGCCAATCCCCAAGTGGGAGTGAGGACATGTCCTGCAATTCTGAAGGGATT | 92464 | 47166 | 730 | 873 | 21235,37122 | -4,733280738 | 0,432654997 | -10,94008106 | 7,41336E-28 | 1,23115E-26 |
| HUS00313625 | Ensembl | ENST00000384769 | Homo_sapiens | snoRNA | ENSG00000207500 | SNORD102 | AGCTTAATGATGACTGTTTTTTTTGATTGCTTGAAGCAATGTGAAAAACACATTTCACCGGCTCTGAAAGCT | 66077 | 94909 | 1423 | 1574 | 25521,28284 | -4,127634097 | 0,379756548 | -10,86915845 | 1,61685E-27 | 2,59254E-26 |
| HUS00283325 | MiRBase | MIMAT0000689 | Homo_sapiens | mature_miRNA | hsa-miR-99b-5p | hsa-miR-99b-5p | CACCCGTAGAACCGACCTTGCG | 12541 | 10826 | 48275 | 59016 | 50019,25791 | 3,670431453 | 0,341005489 | 10,76355533 | 5,11576E-27 | 7,92943E-26 |
| HUS00085870 | Ensembl | ENST00000364968 | Homo_sapiens | snoRNA | ENSG00000278261 | SNORD1A | ACAAGCCTATGATGGTTAGTTATCCCTGTCTGAAAATCTGGACTGAGGGAAATAATCTATTCTGAGGCTTAA | 4048 | 1917 | 38 | 27 | 903,4437499 | -4,828660504 | 0,455048344 | -10,61131321 | 2,64012E-26 | 3,96018E-25 |
| HUS00161321 | GtRNA\|GtRNA\|GtRNA\|GtRNA\|GtRNA\|GtRNA\|GtRNA\|GtRNA\|GtRNA\|GtRNA\|GtRNA\|GtRNA | chr1\|chr1\|chr1\|chr1\|chr1\|chr1\|chr1\|chr1\|chr1\|chr1\|chr1\|chr1 | Homo_sapiens | tRNA | trna117-GlyTCC | trna117-GlyTCC | GCGTTGGTGGTATAGTGGTGAGCATAGCTGCCTTCCAAGCAGTTGACCCGGGTTCGATTCCCGGCCAACGCA | 88 | 156 | 1308 | 833 | 928,5513451 | 4,459965388 | 0,421633688 | 10,57782032 | 3,77641E-26 | 5,4876E-25 |
| HUS00078518 | Ensembl | ENST00000387449 | Homo_sapiens | Mt_tRNA | ENSG00000210184 | MT-TS2 | GAGAAAGCTCACAAGAACTGCTAACTCATGCCCCCATGTCTAACAACATGGCTTTCTCA | 7282 | 9310 | 112 | 157 | 2608,481325 | -4,281834775 | 0,412944633 | -10,36902876 | 3,42993E-25 | 4,83308E-24 |
| HUS00010962 | Ensembl | ENST00000384693 | Homo_sapiens | snoRNA | ENSG00000277846 | SNORD30 | GTTTGTGATGACTTACATGGAATCTCGTTCGGCTGATGACTTGCTGTTGAGACTCTGAAATCTGATTTTC | 401581 | 542645 | 9789 | 9518 | 150178,4475 | -3,998864049 | 0,390744926 | -10,23395003 | 1,39702E-24 | 1,91063E-23 |
| HUS00006456 | MiRBase | MIMAT0005792 | Homo_sapiens | mature_miRNA | hsa-miR-320b | hsa-miR-320b | AAAAGCTGGGTTGAGAGGGCAA | 555 | 672 | 3352 | 1897 | 2354,404883 | 3,491406425 | 0,342846399 | 10,18358787 | 2,34743E-24 | 3,11873E-23 |
| HUS00298571 | Ensembl | ENST00000387377 | Homo_sapiens | Mt_tRNA | ENSG00000210112 | MT-TM | AGTAAGGTCAGCTAAATAAGCTATCGGGCCCATACCCCGAAAATGTTGGTTATACCCTTCCCGTACTA | 5664 | 7188 | 137 | 51 | 2004,961016 | -4,505641362 | 0,444507833 | -10,13624738 | 3,81473E-24 | 4,92736E-23 |
| HUS00059668 | Ensembl | ENST00000386062 | Homo_sapiens | snoRNA | ENSG00000208797 | SNORD73A | AATAAGTGATGAAAAAAGTTTCGGTCCCAGATGATGGCCAGTGATAACAACATTTTTCTGATGTT | 1067 | 1144 | 36 | 20 | 353,7458351 | -3,737465875 | 0,369311582 | -10,12008844 | 4,50009E-24 | 5,65552E-23 |
| HUS00175675 | GtRNA\|GtRNA | chr19\|chr19 | Homo_sapiens | tRNA | trna2-GlyTCC | trna2-GlyTCC | GCGTTGGTGGTATAGTGGTTAGCATAGCTGCCTTCCAAGCAGTTGACCCGGGTTCGATTCCCGGCCAACGCA | 52 | 86 | 558 | 335 | 391,4022185 | 4,03854702 | 0,411001771 | 9,826106122 | 8,69146E-23 | 1,06356E-21 |
| HUS00156066 | Ensembl\|Ensembl | ENST00000363961\|ENST00000630949 | Homo_sapiens | snoRNA | ENSG00000200831 | SNORD36B | TTGCAGTGATGTAAAATTTCTTGGCCTGAAATTACTGTGAAGAGTAAAACCGAGCTTTTTAACACTGAGTCAGC | 2479 | 2450 | 127 | 101 | 831,9695442 | -2,891971391 | 0,295149597 | -9,798324047 | 1,14469E-22 | 1,36482E-21 |
| HUS00147596 | MiRBase | MIMAT0004808 | Homo_sapiens | mature_miRNA | hsa-miR-625-3p | hsa-miR-625-3p | GACTATAGAACTTTCCCCCTCA | 66 | 89 | 336 | 306 | 296,7198237 | 3,479171704 | 0,356811456 | 9,750728701 | 1,83149E-22 | 2,12911E-21 |
| HUS00223268 | GtRNA\|GtRNA\|GtRNA\|GtRNA | chr1\|chr1\|chr1\|chr1 | Homo_sapiens | tRNA | trna133-GluTTC | trna133-GluTTC | TCCCTGGTGGTCTAGTGGCTAGGATTCGGCGCTTTCACCGCCGCGGCCCGGGTTCGATTCCCGGTCAGGGAA | 11 | 20 | 143 | 93 | 103,1029525 | 4,259794123 | 0,438594403 | 9,712376832 | 2,67036E-22 | 3,02858E-21 |
| HUS00109662 | Ensembl | ENST00000408573 | Homo_sapiens | snoRNA | ENSG00000221500 | SNORD100 | GCTGTACATGATGACAACTGGCTCCCTCTACTGAACTGCCATGAGGAAACTGCCATGTCACCCTTCTGATTACAGC | 179060 | 81164 | 1843 | 598 | 39169,38521 | -5,040880486 | 0,520672934 | -9,681472098 | 3,61473E-22 | 4,00202E-21 |
| HUS00067660 | Ensembl | ENST00000364009 | Homo_sapiens | snoRNA | ENSG00000200879 | SNORD14E | TCACAATGATGAATGGTCCAAAACATTCGCGGTTTCCACCAGAATTCAAGGTGTTGGCAACTACCTTCCTTGGATGTCTGAGTGA | 1057 | 1283 | 38 | 20 | 374,7591724 | -3,764370491 | 0,392680406 | -9,586346642 | 9,12593E-22 | 9,86874E-21 |
| HUS00345871 | Ensembl | ENST00000383894 | Homo_sapiens | snoRNA | ENSG00000206621 | SNORD116-14 | GGATCGATGATGACTTCCATATATACATTCCTTGGAAAGCTGAACAAAATGAGTGAAAACTCTATACCGTCATTCTCGTCGAACTGAGGTCC | 409 | 460 | 2703 | 1297 | 1768,066523 | 3,569554782 | 0,374558714 | 9,53002734 | 1,57242E-21 | 1,66176E-20 |
| HUS00094889 | Ensembl\|Ensembl | ENST00000384729\|ENST00000383929 | Homo_sapiens | snoRNA | ENSG00000207460 | SNORD116-19 | GGATCGATGATGACTTCCATATATACATTCCTTGGAAAGCTGAACAAAATGAGTGAAAACTCTATACCGTCATCCTCGTCGAACTGAGGTCC | 436 | 486 | 2744 | 1332 | 1808,208707 | 3,515848625 | 0,37021861 | 9,496682571 | 2,16683E-21 | 2,19302E-20 |
| HUS00344916 | Ensembl | ENST00000384445 | Homo_sapiens | snoRNA | ENSG00000207174 | SNORD116-15 | GGATCGATGATGACTTCCATATATACATTCCTTGGAAAGCTGAACAAAATGAGTGAAAACTCTATACCGTCATCCTCGTCAAACTGAGGTCC | 424 | 474 | 2720 | 1312 | 1785,990844 | 3,536148382 | 0,372361087 | 9,496557247 | 2,16944E-21 | 2,19302E-20 |
| HUS00143452 | Ensembl | ENST00000387459 | Homo_sapiens | Mt_tRNA | ENSG00000210194 | MT-TE | GTTCTTGTAGTTGAAATACAACGATGGTTTTTCATATCATTGGTCGTGGTTGTAGTCCGTGCGAGAATA | 10327 | 14457 | 397 | 383 | 4061,442339 | -3,40728845 | 0,363333673 | -9,377849358 | 6,73321E-21 | 6,66158E-20 |
| HUS00310690 | PiRNA | DQ597971.1 | Homo_sapiens | piRNA | piR-36037 | piR-36037 | GGCCGGTTAGCTCAGTTGGTTAGAGC | 29 | 14 | 263 | 121 | 163,1871061 | 4,451247673 | 0,475465347 | 9,361876109 | 7,83332E-21 | 7,58853E-20 |
| HUS00154737 | Ensembl | ENST00000384529 | Homo_sapiens | snoRNA | ENSG00000278715 | SNORD116-20 | GGATCGATGATGACTTCCATATATACATTCCTTGGAAAGCTGAACAAAATGAGTGAAAACTCTATACTGTCATCCTCGTCGAACTGAGGTCC | 422 | 470 | 2689 | 1269 | 1752,733764 | 3,515266675 | 0,377939056 | 9,301146883 | 1,38939E-20 | 1,3185E-19 |
| HUS00280903 | Ensembl | ENST00000387314 | Homo_sapiens | Mt_tRNA | ENSG00000210049 | MT-TF | GTTTATGTAGCTTACCTCCTCAAAGCAATACACTGAAAATGTTTAGACGGGCTCACATCACCCCATAAACA | 1227 | 2431 | 28 | 14 | 571,4888796 | -4,767302976 | 0,514714829 | -9,262027642 | 2,00586E-20 | 1,86545E-19 |
| HUS00333923 | Ensembl | ENST00000387441 | Homo_sapiens | Mt_tRNA | ENSG00000210176 | MT-TH | GTAAATATAGTTTAACCAAAACATCAGATTGTGAATCTGACAACAGAGGCTTACGACCCCTTATTTACC | 14996 | 21456 | 669 | 509 | 5981,807749 | -3,389026263 | 0,367785568 | -9,214679854 | 3,12209E-20 | 2,84661E-19 |
| HUS00068581 | Ensembl | ENST00000386683 | Homo_sapiens | snoRNA | ENSG00000276610 | SNORD64 | GGATTTGTGATGAGCTGTGTTTACTGAGCATGATGAAGTAAAGCTCAACGTGATTACTCTGAAGTCC | 8980 | 19596 | 229 | 197 | 4518,690826 | -4,407142847 | 0,478426479 | -9,211745249 | 3,20865E-20 | 2,86927E-19 |
| HUS00196652 | MiRBase | MIMAT0005923 | Homo_sapiens | mature_miRNA | hsa-miR-1269a | hsa-miR-1269a | CTGGACTGAGCCGTGCTACTGG | 255 | 182 | 787 | 504 | 602,5986627 | 3,000007028 | 0,327100569 | 9,171512723 | 4,66427E-20 | 4,09224E-19 |
| HUS00172894 | MiRBase | MIMAT0004774 | Homo_sapiens | mature_miRNA | hsa-miR-501-3p | hsa-miR-501-3p | AATGCACCCGGGCAAGGATTCT | 279 | 189 | 812 | 561 | 643,9633832 | 2,997974611 | 0,327514648 | 9,153711539 | 5,50103E-20 | 4,737E-19 |
| HUS00027641 | Ensembl | ENST00000365382 | Homo_sapiens | snoRNA | ENSG00000202252 | SNORD14C | TCGCTGTGATGAGTGATTGTTAAACATTCGTAGTTTCCACCAAAAGCTTGGCTAATGATGGCAACACCTTCCTTGGATGTCTGAGCGA | 236 | 94 | 1230 | 1054 | 1017,324326 | 4,171356977 | 0,458206677 | 9,103658202 | 8,73393E-20 | 7,38414E-19 |
| HUS00099216 | MiRBase | MIMAT0000443 | Homo_sapiens | mature_miRNA | hsa-miR-125a-5p | hsa-miR-125a-5p | TCCCTGAGACCCTTTAACCTGTGA | 7115 | 3950 | 33369 | 17797 | 22712,71417 | 3,594022051 | 0,399531541 | 8,995590301 | 2,34966E-19 | 1,95106E-18 |
| HUS00213419 | MiRBase | MIMAT0004482 | Homo_sapiens | mature_miRNA | hsa-let-7b-3p | hsa-let-7b-3p | CTATACAACCTACTGCCTTCCC | 34 | 17 | 143 | 103 | 110,7356844 | 3,670445973 | 0,408634934 | 8,98221289 | 2,65375E-19 | 2,1649E-18 |
| HUS00364807 | MiRBase | MIMAT0000751 | Homo_sapiens | mature_miRNA | hsa-miR-330-3p | hsa-miR-330-3p | GCAAAGCACACGGCCTGCAGAGA | 252 | 232 | 724 | 459 | 564,6734719 | 2,730077592 | 0,304171736 | 8,975447983 | 2,82201E-19 | 2,26247E-18 |
| HUS00045094 | MiRBase | MIMAT0005793 | Homo_sapiens | mature_miRNA | hsa-miR-320c | hsa-miR-320c | AAAAGCTGGGTTGAGAGGGT | 277 | 340 | 1512 | 804 | 1046,640726 | 3,291294423 | 0,367304408 | 8,960672263 | 3,22705E-19 | 2,54335E-18 |
| HUS00115005 | Ensembl | ENST00000384533 | Homo_sapiens | snoRNA | ENSG00000207263 | SNORD116-16 | GGATCGATGATGACTTTCATACATGCATTCCTTGGAAAGCTGAACAAAATGAGTGAAAACTCTATACCGTCATCCTCGTCGAACTGAGGTCC | 158 | 150 | 476 | 301 | 369,386833 | 2,772616699 | 0,309869982 | 8,9476776 | 3,6304E-19 | 2,81356E-18 |
| HUS00274487 | Ensembl | ENST00000459083 | Homo_sapiens | snoRNA | ENSG00000238597 | SNORD4B | GGTGCAAATGATGCATATGTTAGCGACCAAAGCCTGATCTTTGCTGATTAGTCATAATTAACTGACTGCACC | 662 | 890 | 23 | 11 | 247,1673191 | -3,912587273 | 0,4421634 | -8,848736166 | 8,8516E-19 | 6,74753E-18 |
| HUS00106991 | Ensembl | ENST00000387372 | Homo_sapiens | Mt_tRNA | ENSG00000210107 | MT-TQ | TAGGATGGGGTGTGATAGGTGGCACGGAGAATTTTGGATTCTCAGGGATGGGTTCGATTCTCATAGTCCTAG | 877 | 2370 | 5 | 11 | 502,013933 | -5,666822261 | 0,648456664 | -8,738937508 | 2,35313E-18 | 1,76485E-17 |
| HUS00120793 | MiRBase | MIMAT0004984 | Homo_sapiens | mature_miRNA | hsa-miR-941 | hsa-miR-941 | CACCCGGCTGTGTGCACATGTGC | 157 | 177 | 432 | 391 | 400,3737855 | 2,762397727 | 0,316560416 | 8,726289151 | 2,63165E-18 | 1,94241E-17 |
| HUS00120931 | MiRBase | MIMAT0000449 | Homo_sapiens | mature_miRNA | hsa-miR-146a-5p | hsa-miR-146a-5p | TGAGAACTGAATTCCATGGGTT | 761 | 455 | 6873 | 2352 | 3888,297927 | 4,195691956 | 0,483777924 | 8,672764394 | 4,21754E-18 | 3,06431E-17 |
| HUS00043489 | MiRBase | MIMAT0005825 | Homo_sapiens | mature_miRNA | hsa-miR-1180-3p | hsa-miR-1180-3p | TTTCCGGCTCGCGTGGGTGTGT | 341 | 237 | 773 | 623 | 675,5792112 | 2,738495934 | 0,315844096 | 8,670404059 | 4,3059E-18 | 3,08037E-17 |
| HUS00047103 | MiRBase | MIMAT0004614 | Homo_sapiens | mature_miRNA | hsa-miR-193a-5p | hsa-miR-193a-5p | TGGGTCTTTGCGGGCGAGATGA | 322 | 241 | 923 | 554 | 696,412929 | 2,824173682 | 0,329511145 | 8,570798656 | 1,0277E-17 | 7,2406E-17 |
| HUS00104947 | MiRBase | MIMAT0022717 | Homo_sapiens | mature_miRNA | hsa-miR-873-3p | hsa-miR-873-3p | GGAGACTGATGAGTTCCCGGGA | 14 | 17 | 74 | 59 | 60,81962028 | 3,498154453 | 0,408713433 | 8,558941712 | 1,13908E-17 | 7,90558E-17 |
| HUS00237647 | Ensembl | ENST00000384507 | Homo_sapiens | snoRNA | ENSG00000277785 | SNORD116-21 | GGATCGATGATGACTTCCACATATACATTCCTTGGAAAGCTGAACAAAATGAGTGAAAACTCTATACCGTCATCCTCGTCGAACTGAGGTCC | 51 | 57 | 305 | 145 | 200,3394953 | 3,41896171 | 0,402264431 | 8,499289149 | 1,90755E-17 | 1,30443E-16 |
| HUS00248547 | Ensembl | ENST00000386967 | Homo_sapiens | snoRNA | ENSG00000209702 | SNORD41 | TGGGAAGTGATGACACCTGTGACTGTTGATGTGGAACTGATTTATCGCGTATTCGTACTGGCTGATCCTG | 195 | 129 | 990 | 439 | 630,4762104 | 3,497798636 | 0,418518148 | 8,357579352 | 6,40182E-17 | 4,31427E-16 |
| HUS00309606 | Ensembl | ENST00000411005 | Homo_sapiens | snoRNA | ENSG00000222937 | SNORD63 | GTGCCGTGATGTATTTGTCAACACATCACTCTGAAGAAAAGTATGTGGTGACTTTCTGTGACTGAGCATG | 1356 | 1901 | 18 | 36 | 514,0393383 | -4,173482761 | 0,503631925 | -8,286771657 | 1,16364E-16 | 7,72988E-16 |
| HUS00096359 | GtRNA\|GtRNA | chr1\|chr1 | Homo_sapiens | tRNA | trna98-ValCAC | trna98-ValCAC | GTTTCCGTAGTGTAGTGGTTATCACGTTCGCCTCACACGCGAAAGGTCCCCGGTTCGAAACTGGGCGGAAACA | 258 | 320 | 901 | 581 | 704,3950941 | 2,7805183 | 0,336133785 | 8,272058405 | 1,31666E-16 | 8,62318E-16 |
| HUS00195952 | MiRBase | MIMAT0005899 | Homo_sapiens | mature_miRNA | hsa-miR-1247-5p | hsa-miR-1247-5p | ACCCGTCCCGTTCGTCCCCGGA | 27 | 27 | 90 | 98 | 89,03313079 | 3,238489602 | 0,392399567 | 8,253040709 | 1,54414E-16 | 9,9726E-16 |
| HUS00135415 | GtRNA\|GtRNA\|GtRNA\|GtRNA\|GtRNA\|GtRNA\|GtRNA\|GtRNA\|GtRNA\|GtRNA\|GtRNA\|GtRNA | chr1\|chr5\|chr5\|chr5\|chr5\|chr6\|chr1\|chr5\|chr5\|chr5\|chr5\|chr6 | Homo_sapiens | tRNA | trna85-ValCAC | trna85-ValCAC | GTTTCCGTAGTGTAGTGGTTATCACGTTCGCCTCACACGCGAAAGGTCCCCGGTTCGAAACCGGGCGGAAACA | 258 | 323 | 917 | 587 | 713,7501515 | 2,792162204 | 0,339096981 | 8,234111057 | 1,80896E-16 | 1,15228E-15 |
| HUS00014348 | MiRBase | MIMAT0005898 | Homo_sapiens | mature_miRNA | hsa-miR-1246 | hsa-miR-1246 | AATGGATTTTTGGAGCAGG | 2380 | 2637 | 175 | 124 | 876,1081783 | -2,540340845 | 0,309521484 | -8,20731671 | 2,26186E-16 | 1,42131E-15 |
| HUS00118170 | Ensembl | ENST00000390861 | Homo_sapiens | snoRNA | ENSG00000274802 | SNORD91A | TAGAGAAGTCAATGATGGTTTTATTCATATCGTCTGAACCTGTCTGAAGCATCTCAGTGATGCAATCTCTGTGTGGTTCTGAGACTTCTCCAAGT | 8999 | 4593 | 280 | 191 | 2196,530978 | -3,254956102 | 0,396748774 | -8,2040735 | 2,32376E-16 | 1,44073E-15 |
| HUS00009855 | Ensembl | ENST00000410433 | Homo_sapiens | snoRNA | ENSG00000222365 | SNORD12B | GCTGGCATATATGATGACTTAGCTTTTTTCCCCGACAGATCGACTATGTTGATCTAACTTTTCTAAGCCAGTTTCTGTCTGATATGCCAGC | 27070 | 53713 | 901 | 370 | 12756,29419 | -4,345063217 | 0,533547727 | -8,143719859 | 3,83316E-16 | 2,34529E-15 |
| HUS00224414 | MiRBase | MIMAT0004980 | Homo_sapiens | mature_miRNA | hsa-miR-937-3p | hsa-miR-937-3p | ATCCGCGCTCTGACTCTCTGCC | 32 | 20 | 107 | 149 | 119,6146522 | 3,720187181 | 0,457009462 | 8,140284804 | 3,94349E-16 | 2,38146E-15 |
| HUS00088227 | MiRBase | MIMAT0000102 | Homo_sapiens | mature_miRNA | hsa-miR-105-5p | hsa-miR-105-5p | TCAAATGCTCAGACTCCTGTGGT | 21 | 25 | 80 | 66 | 68,68892823 | 3,08871124 | 0,383530257 | 8,053370463 | 8,05446E-16 | 4,8017E-15 |
| HUS00187563 | MiRBase | MIMAT0003322 | Homo_sapiens | mature_miRNA | hsa-miR-652-3p | hsa-miR-652-3p | AATGGCGCCACTAGGGTTGTG | 307 | 284 | 658 | 437 | 545,2955404 | 2,342740233 | 0,294171209 | 7,963866499 | 1,66745E-15 | 9,81475E-15 |
| HUS00025934 | Ensembl | ENST00000577887 | Homo_sapiens | snoRNA | ENSG00000265706 | SNORD53_SNORD92 | TTGCTGTGATGACTATCATTGGGTTTCGCATGTTGCTGAGTTCCAGTGATGCCTCTTTTCTCTTGGCTGTCTGAGCAA | 48792 | 62416 | 1263 | 1851 | 18060,63812 | -3,494847283 | 0,439309335 | -7,95532215 | 1,78666E-15 | 1,0385E-14 |
| HUS00029886 | Ensembl | ENST00000363091 | Homo_sapiens | snoRNA | ENSG00000199961 | SNORD1B | CTGAGTCCATGATGATTTCAAGTTATCCCTGTCTGAAGGCAAAGAAAGGCCTTTCTGTGTGGAATTTGAATATCTGAAACTCAG | 1809 | 1536 | 116 | 102 | 590,1985978 | -2,390838139 | 0,301440962 | -7,931364489 | 2,16751E-15 | 1,24431E-14 |
| HUS00127541 | Ensembl | ENST00000408061 | Homo_sapiens | snoRNA | ENSG00000220988 | SNORD88C | GGGCTCCCATGATGTCCAGCACTGGGCTCTGATCACCCCTGAGGACACAGTGCACCCCAGGACCTTTGACACCTGGGGGTCTGAGGGGCCC | 53797 | 71479 | 1647 | 2290 | 20543,85762 | -3,346943846 | 0,424451545 | -7,885337875 | 3,13686E-15 | 1,77883E-14 |
| HUS00111477 | Ensembl | ENST00000579879 | Homo_sapiens | snoRNA | ENSG00000264549 | SNORD95 | GGGGCGGTGATGACCCCAACATGCCATCTGAGTGTCGGTGCTGAAATCCAGAGGCTGTTTCTGAGCTG | 37223 | 23037 | 1534 | 917 | 9917,832504 | -3,050623272 | 0,389034069 | -7,841532437 | 4,45081E-15 | 2,49353E-14 |
| HUS00061181 | Ensembl | ENST00000365444 | Homo_sapiens | snoRNA | ENSG00000202314 | SNORD6 | CGATGTTATGATGATGGGCGAAATGTTCAACTGCTCTGAAGGGGCTGAATGAAAATGGCCTTTCTGAACATCC | 98506 | 85627 | 3094 | 4400 | 30684,49059 | -2,988225226 | 0,381398765 | -7,834910605 | 4,69176E-15 | 2,59723E-14 |
| HUS00292976 | Ensembl | ENST00000387400 | Homo_sapiens | Mt_tRNA | ENSG00000210135 | MT-TN | TAGATTGAAGCCAGTTGATTAGGGTGCTTAGCTGTTAACTAAGTGTTTGTGGGTTTAAGTCCCATTGGTCTAG | 504 | 1679 | 11 | 5 | 340,3714383 | -5,201862113 | 0,664324832 | -7,830299069 | 4,86711E-15 | 2,66259E-14 |
| HUS00334425 | MiRBase | MIMAT0000279 | Homo_sapiens | mature_miRNA | hsa-miR-222-3p | hsa-miR-222-3p | AGCTACATCTGGCTACTGGGT | 12949 | 9884 | 28290 | 17104 | 22233,5332 | 2,435981272 | 0,311271565 | 7,825903644 | 5,04023E-15 | 2,72524E-14 |
| HUS00149351 | MiRBase | MIMAT0000092 | Homo_sapiens | mature_miRNA | hsa-miR-92a-3p | hsa-miR-92a-3p | TATTGCACTTGTCCCGGCCTGT | 32602 | 37682 | 71161 | 72622 | 72203,82407 | 2,506556684 | 0,322933828 | 7,761827556 | 8,37142E-15 | 4,47438E-14 |
| HUS00225456 | Ensembl | ENST00000387405 | Homo_sapiens | Mt_tRNA | ENSG00000210140 | MT-TC | AGCTCCGAGGTGATTTTCATATTGAATTGCAAATTCGAAGAAGCAGCTTCAAACCTGCCGGGGCTT | 984 | 1976 | 34 | 10 | 466,1684626 | -4,39593535 | 0,567084274 | -7,75182023 | 9,05846E-15 | 4,78657E-14 |
| HUS00310391 | MiRBase | MIMAT0000510 | Homo_sapiens | mature_miRNA | hsa-miR-320a | hsa-miR-320a | AAAAGCTGGGTTGAGAGGGCGA | 5180 | 5938 | 17762 | 10169 | 13222,32598 | 2,734426521 | 0,36188493 | 7,55606629 | 4,15442E-14 | 2,17057E-13 |
| HUS00311403 | MiRBase | MIMAT0004680 | Homo_sapiens | mature_miRNA | hsa-miR-130b-5p | hsa-miR-130b-5p | ACTCTTTCCCTGTTGCACTAC | 95 | 60 | 206 | 243 | 217,2508544 | 2,999307038 | 0,397902673 | 7,537790622 | 4,78001E-14 | 2,46967E-13 |
| HUS00365058 | Ensembl | ENST00000625943 | Homo_sapiens | snoRNA | ENSG00000281859 | SNORD38B | TCTCAGTGATGAAAACTTTGTCCAGTTCTGCTACTGACAGTAAGTGAAGATAAAGTGTGTCTGAGGAGA | 949 | 869 | 37 | 48 | 307,8897514 | -2,817110193 | 0,374188422 | -7,528587284 | 5,12922E-14 | 2,62098E-13 |
| HUS00062368 | Ensembl | ENST00000363405 | Homo_sapiens | rRNA | ENSG00000200275 | RNA5SP199 | GTCTACGGCCATACCACCCTGAACGCGCCTGATCTCGTCTGATCTCGGAAGCTAAGCAGGGTCGGGCCTGCTTAGTACTTGGATGGGAGACA | 257 | 235 | 538 | 673 | 599,37113 | 2,777274737 | 0,370472972 | 7,496565068 | 6,55119E-14 | 3,3112E-13 |
| HUS00207896 | Ensembl | ENST00000384690 | Homo_sapiens | snoRNA | ENSG00000207421 | SNORD38B | TCTCAGTGATGAAAACTTTGTCCAGTTCTGCTACTGACAGTAAGTGAAGATAAAGTGTGTCTGAGGA | 941 | 852 | 37 | 48 | 304,0966254 | -2,797233499 | 0,374235178 | -7,47453383 | 7,7478E-14 | 3,8739E-13 |
| HUS00060652 | MiRBase | MIMAT0004748 | Homo_sapiens | mature_miRNA | hsa-miR-423-5p | hsa-miR-423-5p | TGAGGGGCAGAGAGCGAGACTTT | 1281 | 1518 | 4065 | 2197 | 3001,665826 | 2,569663247 | 0,347490783 | 7,394910524 | 1,41504E-13 | 6,99991E-13 |
| HUS00189229 | Ensembl | ENST00000384430 | Homo_sapiens | snoRNA | ENSG00000275127 | SNORD116-22 | GGATCGATGATGACTTCCATATGTACATTCCTTGGAAAGCTGAACAAAATGAGTGAAAACTCTATACCGTCATCCTCGTCGAACTGAGGTCC | 117 | 100 | 466 | 202 | 304,2551397 | 2,992053457 | 0,404907671 | 7,389470917 | 1,47414E-13 | 7,21554E-13 |
| HUS00127643 | MiRBase | MIMAT0004502 | Homo_sapiens | mature_miRNA | hsa-miR-28-3p | hsa-miR-28-3p | CACTAGATTGTGAGCTCCTGGA | 1184 | 1319 | 2147 | 1770 | 2032,390805 | 2,116850869 | 0,286545413 | 7,387488246 | 1,49628E-13 | 7,24762E-13 |
| HUS00198314 | GtRNA\|GtRNA | chr1\|chr1 | Homo_sapiens | tRNA | trna130-GlyCCC | trna130-GlyCCC | GCGTTGGTGGTTTAGTGGTAGAATTCTCGCCTCCCATGCGGGAGACCCGGGTTCAATTCCCGGCCACTGCA | 627 | 1464 | 7 | 17 | 328,6449358 | -4,567954718 | 0,619688416 | -7,371373415 | 1,68879E-13 | 8,09575E-13 |
| HUS00000142 | Rfam\|Ensembl | RF00005\|ENST00000387419 | Homo_sapiens | tRNA | ENSG00000210154 | MT-TD | AAGGTATTAGAAAAACCATTTCATAACTTTGTCAAAGTTAAATTATAGGCTAAATCCTATATATCTTA | 219 | 260 | 0 | 2 | 72,75436937 | -5,555541001 | 0,764851631 | -7,263553845 | 3,7705E-13 | 1,78906E-12 |
| HUS00053878 | Ensembl | ENST00000387382 | Homo_sapiens | Mt_tRNA | ENSG00000210117 | MT-TW | AGAAATTTAGGTTAAATACAGACCAAGAGCCTTCAAAGCCCTCAGTAAGTTGCAATACTTAATTTCTG | 225 | 463 | 0 | 0 | 104,3215515 | -6,944131097 | 0,966537874 | -7,184541119 | 6,74333E-13 | 3,16732E-12 |
| HUS00030405 | Ensembl | ENST00000391196 | Homo_sapiens | snoRNA | ENSG00000212498 | SNORD86 | GATCACGGTGATGGCTGACCAGGGCTCCCTGACCTATACAGGCCTCTGCTATGGGGGTGATGGCCAGTCCTGGTGTCTGAGTGATT | 258 | 355 | 11 | 7 | 99,66955562 | -3,463476708 | 0,482399885 | -7,179679799 | 6,98749E-13 | 3,21701E-12 |
| HUS00280833 | MiRBase | MIMAT0004485 | Homo_sapiens | mature_miRNA | hsa-let-7e-3p | hsa-let-7e-3p | CTATACGGCCTCCTAGCTTTCC | 42 | 31 | 87 | 72 | 78,13430492 | 2,576195961 | 0,358814983 | 7,179733518 | 6,98474E-13 | 3,21701E-12 |
| HUS00258731 | PiRNA | DQ598649.1 | Homo_sapiens | piRNA | piR-36715 | piR-36715 | GTTCAGTGATGAGGCCTGGAATGTGCGCTGG | 404 | 561 | 22 | 18 | 162,0518216 | -3,009035999 | 0,421294318 | -7,142360745 | 9,17414E-13 | 4,18233E-12 |
| HUS00021408 | Ensembl\|Ensembl\|Ensembl\|Ensembl | ENST00000583032\|ENST00000582400\|ENST00000577237\|ENST00000578807 | Homo_sapiens | snoRNA | ENSG00000265732 | SNORD48 | AGTGATGATGACCCCAGGTAACTCTGAGTGTGTCGCTGATGCCATCACCGCAGCGCTCTGACC | 35573 | 21284 | 1072 | 1236 | 9389,836918 | -2,982864723 | 0,418141021 | -7,133633327 | 9,77535E-13 | 4,41314E-12 |
| HUS00183978 | PiRNA | DQ598650.1 | Homo_sapiens | piRNA | piR-36716 | piR-36716 | GTTCAGTGATGAGGCCTGGAATGTGCGCTGGG | 405 | 563 | 23 | 19 | 163,3632726 | -2,945765972 | 0,418752162 | -7,034628684 | 1,99792E-12 | 8,84795E-12 |
| HUS00155913 | MiRBase | MIMAT0000450 | Homo_sapiens | mature_miRNA | hsa-miR-149-5p | hsa-miR-149-5p | TCTGGCTCCGTGTCTTCACTCCC | 315 | 279 | 549 | 363 | 469,1402831 | 2,076324395 | 0,295117891 | 7,035576143 | 1,98439E-12 | 8,84795E-12 |
| HUS00283395 | Ensembl | ENST00000516336 | Homo_sapiens | snRNA | ENSG00000252145 | RNU6-1225P | GTGTTTTGGCAGTACATATACTAAAATTGGAACGATACAGAGAAGATTAGCATGGCCCCTGCCCA | 100 | 138 | 308 | 433 | 359,9645438 | 3,076436286 | 0,437474843 | 7,032258738 | 2,03217E-12 | 8,91469E-12 |
| HUS00312514 | GtRNA\|GtRNA\|GtRNA\|GtRNA\|GtRNA\|GtRNA\|GtRNA\|GtRNA\|GtRNA\|GtRNA\|GtRNA\|GtRNA\|GtRNA\|GtRNA\|GtRNA\|GtRNA\|GtRNA\|GtRNA | chr1\|chr1\|chr1\|chr1\|chr15\|chr15\|chr15\|chr6\|chr9\|chr1\|chr1\|chr1\|chr1\|chr15\|chr15\|chr15\|chr6\|chr9 | Homo_sapiens | tRNA | trna111-HisGTG | trna111-HisGTG | GCCGTGATCGTATAGTGGTTAGTACTCTGCGTTGTGGCCGCAGCAACCTCGGTTCGAATCCGAGTCACGGCA | 669 | 860 | 1776 | 2569 | 2133,661169 | 2,960060588 | 0,423892805 | 6,98304042 | 2,88859E-12 | 1,25532E-11 |
| HUS00299514 | Ensembl | ENST00000408314 | Homo_sapiens | snoRNA | ENSG00000221241 | SNORD88A | GGGCCTCCATGATGTCCAGCACTGGGCTCCGACTGCCACTGAGGACACGGTGCCCCCCGGGACCTTTGACACCCGGGGGTCTGAGGGGCCC | 1899 | 1743 | 181 | 120 | 668,3579857 | -2,077593759 | 0,297713992 | -6,978488793 | 2,98372E-12 | 1,28466E-11 |
| HUS00258330 | Ensembl | ENST00000384711 | Homo_sapiens | snoRNA | ENSG00000207442 | SNORD116-6 | GGATCGATGATGAGTCCTCCAAAAAAAACATTCCTTGGAAAAGCTGAACAAAATGAGTGAAAACTCATACCGTCATTCTCATCGGAACTGAGGTCC | 137 | 100 | 276 | 169 | 220,0184402 | 2,350931768 | 0,338561822 | 6,943877354 | 3,81482E-12 | 1,62742E-11 |
| HUS00205868 | Ensembl | ENST00000387439 | Homo_sapiens | Mt_tRNA | ENSG00000210174 | MT-TR | TGGTATATAGTTTAAACAAAACGAATGATTTCGACTCATTAAATTATGATAATCATATTTACCAA | 228 | 152 | 2 | 1 | 57,45194859 | -4,903972403 | 0,714219409 | -6,86619874 | 6,59352E-12 | 2,78726E-11 |
| HUS00070814 | PiRNA | DQ598648.1 | Homo_sapiens | piRNA | piR-36714 | piR-36714 | GTTCAGTGATGAGGCCTGGAATGTGCGCTG | 364 | 509 | 22 | 17 | 147,7533266 | -2,90602937 | 0,424199178 | -6,850624712 | 7,35282E-12 | 3,08023E-11 |
| HUS00018149 | PiRNA | DQ598647.1 | Homo_sapiens | piRNA | piR-36713 | piR-36713 | GTTCAGTGATGAGGCCTGGAATGTGCGCT | 330 | 461 | 19 | 15 | 133,3282953 | -2,954944938 | 0,43252829 | -6,831795753 | 8,38582E-12 | 3,48161E-11 |
| HUS00242017 | MiRBase | MIMAT0004672 | Homo_sapiens | mature_miRNA | hsa-miR-106b-3p | hsa-miR-106b-3p | CCGCACTGTGGGTACTTGCTGC | 1210 | 1344 | 1939 | 1777 | 1965,456015 | 2,020281658 | 0,298272725 | 6,773269868 | 1,25904E-11 | 5,181E-11 |
| HUS00024483 | MiRBase | MIMAT0000226 | Homo_sapiens | mature_miRNA | hsa-miR-196a-5p | hsa-miR-196a-5p | TAGGTAGTTTCATGTTGTTGGG | 89 | 97 | 185 | 126 | 157,8836561 | 2,188414352 | 0,325525932 | 6,722703597 | 1,78383E-11 | 7,27616E-11 |
| HUS00334439 | Ensembl | ENST00000384229 | Homo_sapiens | snoRNA | ENSG00000277370 | SNORD49A | TGCTCTGATGAAATCACTAATAGGAAGTGCCGTCAGAAGCGATAACTGACGAAGACTACTCCTGTCTGATT | 947654 | 1801113 | 10659 | 17395 | 428519,9988 | -4,630293918 | 0,6963843 | -6,649049844 | 2,94991E-11 | 1,19279E-10 |
| HUS00142384 | GtRNA\|GtRNA | chr1\|chr1 | Homo_sapiens | tRNA | trna56-ThrTGT | trna56-ThrTGT | GGCTCCATAGCTCAGTGGTTAGAGCACTGGTCTTGTAAACCAGGGGTCGCGAGTTCGATCCTCGCTGGGGCCT | 222 | 131 | 3 | 1 | 53,69437191 | -4,506039132 | 0,682867516 | -6,598701839 | 4,14774E-11 | 1,66267E-10 |
| HUS00023718 | Ensembl | ENST00000459386 | Homo_sapiens | snoRNA | ENSG00000238886 | SNORD121A | GTCCAGAAAACAATGATGTGGTAATTTCCAAGCACATATCTGATGATTCCATGTGGAATTTAACTACCTGAGTTTCCTGGAC | 522 | 304 | 22 | 16 | 137,8062982 | -2,838589333 | 0,431297511 | -6,581511047 | 4,65691E-11 | 1,85082E-10 |
| HUS00189379 | Ensembl | ENST00000387409 | Homo_sapiens | Mt_tRNA | ENSG00000210144 | MT-TY | GGTAAAATGGCTGAGTGAAGCATTGGACTGTAAATCTAAAGACAGGGGTTAGGCCTCTTTTTACCA | 959 | 4133 | 11 | 31 | 800,6951217 | -4,834648244 | 0,735722813 | -6,571290382 | 4,98811E-11 | 1,96565E-10 |
| HUS00076751 | MiRBase | MIMAT0017985 | Homo_sapiens | mature_miRNA | hsa-miR-3607-3p | hsa-miR-3607-3p | ACTGTAAACGCTTTCTGATG | 173 | 603 | 1 | 4 | 121,0578983 | -5,068759025 | 0,77336079 | -6,554197069 | 5,59421E-11 | 2,18597E-10 |
| HUS00250686 | MiRBase | MIMAT0000617 | Homo_sapiens | mature_miRNA | hsa-miR-200c-3p | hsa-miR-200c-3p | TAATACTGCCGGGTAATGATGGA | 95 | 61 | 180 | 115 | 145,9441692 | 2,36131886 | 0,361029062 | 6,540522944 | 6,13041E-11 | 2,37553E-10 |
| HUS00196788 | MiRBase | MIMAT0010133 | Homo_sapiens | mature_miRNA | hsa-miR-2110 | hsa-miR-2110 | TTGGGGAAACGGCCGCTGAGTG | 27 | 15 | 56 | 82 | 66,70871848 | 3,147118331 | 0,481329719 | 6,538383585 | 6,21873E-11 | 2,38984E-10 |
| HUS00140058 | Ensembl | ENST00000384549 | Homo_sapiens | snoRNA | ENSG00000207279 | SNORD116-24 | GGATCGATGATGACTTTTATACATGCATTCCTTGGAAAGCTGAACAAAATGAGTGAAAACTCTATACCGTCATCTTCGTTGAACTGAGGTCC | 251 | 284 | 589 | 339 | 464,2081329 | 2,219929545 | 0,339695476 | 6,535057722 | 6,3585E-11 | 2,42353E-10 |
| HUS00326777 | Ensembl | ENST00000365161 | Homo_sapiens | snoRNA | ENSG00000202031 | SNORD38A | TCTCGTGATGAAAACTCTGTCCAGTTCTGCTACTGAAGGGAGAGAGATGAGAGCCTTTTAGGCTGAGGAA | 6860 | 6889 | 563 | 234 | 2376,625548 | -2,592341182 | 0,398545626 | -6,504502899 | 7,79507E-11 | 2,92907E-10 |
| HUS00301195 | MiRBase | MIMAT0000074 | Homo_sapiens | mature_miRNA | hsa-miR-19b-3p | hsa-miR-19b-3p | TGTGCAAATCCATGCAAAACTGA | 7418 | 6230 | 588 | 258 | 2374,278016 | -2,493618046 | 0,383385897 | -6,504198688 | 7,81086E-11 | 2,92907E-10 |
| HUS00224812 | Rfam\|Ensembl | RF00005\|ENST00000387429 | Homo_sapiens | tRNA | ENSG00000210164 | MT-TG | ACTCTTTTAGTATAAATAGTACCGTTAACTTCCAATTAACTAGTTTTGACAACATTCAAAAAAGAGTA | 183 | 193 | 10 | 2 | 60,91373287 | -3,335679477 | 0,519153504 | -6,425227705 | 1,31672E-10 | 4,89821E-10 |
| HUS00149858 | MiRBase | MIMAT0000253 | Homo_sapiens | mature_miRNA | hsa-miR-10a-5p | hsa-miR-10a-5p | TACCCTGTAGATCCGAATTTGTG | 15577 | 18945 | 26079 | 21157 | 25148,20217 | 1,919101041 | 0,298972285 | 6,418993115 | 1,37179E-10 | 5,06254E-10 |
| HUS00011935 | Ensembl | ENST00000383961 | Homo_sapiens | snoRNA | ENSG00000206688 | SNORD116-18 | GGATCGATGATGACTTCCTTATATACATTCCTTGGAAAGCTGAACAAAATGAGTGAAAACTCTATACCGTCATCCTCGTCGAACTGAGGTCC | 31 | 26 | 57 | 67 | 62,13682201 | 2,580687246 | 0,403513309 | 6,395544308 | 1,59976E-10 | 5,85741E-10 |
| HUS00138565 | Rfam\|Ensembl | RF00005\|ENST00000387365 | Homo_sapiens | tRNA | ENSG00000210100 | MT-TI | AGAAATATGTCTGATAAAAGAGTTACTTTGATAGAGTAAATAATAGGAGCTTAAACCCCCTTATTTCTA | 141 | 175 | 4 | 4 | 50,83452383 | -3,589598069 | 0,563630238 | -6,368710948 | 1,90623E-10 | 6,92499E-10 |
| HUS00285163 | MiRBase | MIMAT0004569 | Homo_sapiens | mature_miRNA | hsa-miR-222-5p | hsa-miR-222-5p | CTCAGTAGCCAGTGTAGATCCT | 51 | 52 | 94 | 116 | 106,5336644 | 2,496346441 | 0,392065101 | 6,367173292 | 1,92544E-10 | 6,94053E-10 |
| HUS00256382 | MiRBase | MIMAT0000703 | Homo_sapiens | mature_miRNA | hsa-miR-361-5p | hsa-miR-361-5p | TTATCAGAATCTCCAGGGGTAC | 1451 | 1212 | 3567 | 1636 | 2521,0782 | 2,368191594 | 0,373001992 | 6,349005217 | 2,16712E-10 | 7,75161E-10 |
| HUS00181846 | MiRBase | MIMAT0000440 | Homo_sapiens | mature_miRNA | hsa-miR-191-5p | hsa-miR-191-5p | CAACGGAATCCCAAAAGCAGCTG | 36030 | 26499 | 66658 | 35236 | 51232,70989 | 2,137233004 | 0,337155563 | 6,339011546 | 2,31244E-10 | 8,20828E-10 |
| HUS00197467 | MiRBase | MIMAT0030019 | Homo_sapiens | mature_miRNA | hsa-miR-7704 | hsa-miR-7704 | CGGGGTCGGCGGCGACGTG | 41 | 27 | 71 | 96 | 82,94698413 | 2,756347904 | 0,436864823 | 6,309383952 | 2,80148E-10 | 9,86886E-10 |
| HUS00045571 | MiRBase | MIMAT0004810 | Homo_sapiens | mature_miRNA | hsa-miR-629-5p | hsa-miR-629-5p | TGGGTTTACGTTGGGAGAACT | 663 | 525 | 911 | 622 | 817,6345522 | 1,835012689 | 0,295490236 | 6,210062012 | 5,29637E-10 | 1,85174E-09 |
| HUS00133308 | MiRBase | MIMAT0000257 | Homo_sapiens | mature_miRNA | hsa-miR-181b-5p | hsa-miR-181b-5p | AACATTCATTGCTGTCGGTGGGT | 616 | 554 | 1250 | 654 | 957,8813035 | 2,127154856 | 0,343370101 | 6,194933254 | 5,83097E-10 | 2,02343E-09 |
| HUS00338313 | MiRBase | MIMAT0000100 | Homo_sapiens | mature_miRNA | hsa-miR-29b-3p | hsa-miR-29b-3p | TAGCACCATTTGAAATCAGTGTT | 3085 | 2138 | 250 | 206 | 966,3810773 | -1,969397793 | 0,319472316 | -6,16453349 | 7,06912E-10 | 2,41702E-09 |
| HUS00274539 | MiRBase | MIMAT0000752 | Homo_sapiens | mature_miRNA | hsa-miR-328-3p | hsa-miR-328-3p | CTGGCCCTCTCTGCCCTTCCGT | 219 | 87 | 432 | 382 | 390,8285679 | 2,842021633 | 0,461017225 | 6,164675595 | 7,06278E-10 | 2,41702E-09 |
| HUS00055226 | Ensembl | ENST00000365172 | Homo_sapiens | snoRNA | ENSG00000277108 | SNORD49 | TGTCCTGATGATACTTGTAATAGGAAGTGCCGTCAGAAGCGATAACTGACGACGTCTAATGTCTATCTGACC | 13199 | 44885 | 405 | 642 | 9346,756211 | -3,950979858 | 0,649274902 | -6,085218831 | 1,16332E-09 | 3,94851E-09 |
| HUS00303483 | Ensembl | ENST00000384048 | Homo_sapiens | snoRNA | ENSG00000206775 | SNORD37 | ATTCGTGATGACTGATCATTTCTTCACTTTGACCAGATGTCTACTGAAGAAAGCCTGCGTCTGAGG | 17107 | 4336 | 282 | 275 | 3353,51188 | -3,497030184 | 0,57539425 | -6,077624487 | 1,21976E-09 | 4,11006E-09 |
| HUS00110928 | Ensembl | ENST00000384147 | Homo_sapiens | snoRNA | ENSG00000276788 | SNORD26 | CTACGGGGATGATTTTACGAACTGAACTCTCTCTTTCTGATGGATTAGTGGAGAAAACAGAAAATTCTGAGTAGC | 6357 | 3991 | 9907 | 12554 | 11293,85732 | 2,5918065 | 0,430429272 | 6,021445721 | 1,72866E-09 | 5,78293E-09 |
| HUS00081981 | Ensembl\|Ensembl | ENST00000383884\|ENST00000627983 | Homo_sapiens | snoRNA | ENSG00000206611 | SNORD24 | TGCAGATGATGTAAAAGAATATTTGCTATCTGAGAGATGGTGATGACATTTTAAACCACCAAGATCGCTGATGCA | 5107 | 1997 | 195 | 112 | 1166,692593 | -2,910073932 | 0,485481619 | -5,994200018 | 2,0449E-09 | 6,79197E-09 |
| HUS00173399 | Ensembl | ENST00000363389 | Homo_sapiens | snoRNA | ENSG00000200259 | SNORD35A | GGCAGATGATGTCCTTATCTCACGATGGTCTGCGGATGTCCCTGTGGGAATGGCGACAATGCCAATGGCTTAGCTGATGCCAGGAG | 154 | 167 | 245 | 174 | 223,6949143 | 1,843387858 | 0,309695707 | 5,952255121 | 2,64473E-09 | 8,72197E-09 |
| HUS00011838 | MiRBase | MIMAT0000423 | Homo_sapiens | mature_miRNA | hsa-miR-125b-5p | hsa-miR-125b-5p | TCCCTGAGACCCTAACTTGTGA | 21637 | 13857 | 53872 | 22005 | 36038,43652 | 2,478765058 | 0,416899342 | 5,945715929 | 2,75251E-09 | 9,01351E-09 |
| HUS00316219 | MiRBase | MIMAT0000078 | Homo_sapiens | mature_miRNA | hsa-miR-23a-3p | hsa-miR-23a-3p | ATCACATTGCCAGGGATTTCC | 22361 | 21706 | 49246 | 22653 | 35945,9631 | 2,1136564 | 0,357284809 | 5,915886558 | 3,30093E-09 | 1,07338E-08 |
| HUS00117841 | MiRBase | MIMAT0000097 | Homo_sapiens | mature_miRNA | hsa-miR-99a-5p | hsa-miR-99a-5p | AACCCGTAGATCCGATCTTGTG | 351 | 324 | 455 | 521 | 522,2706071 | 2,025735944 | 0,344466297 | 5,880795773 | 4,08299E-09 | 1,31846E-08 |
| HUS00141734 | GtRNA\|GtRNA\|GtRNA\|GtRNA\|GtRNA\|GtRNA\|GtRNA\|GtRNA\|GtRNA\|GtRNA | chr1\|chr1\|chr1\|chr1\|chr21\|chr1\|chr1\|chr1\|chr1\|chr21 | Homo_sapiens | tRNA | trna35-GlyGCC | trna35-GlyGCC | GCATGGGTGGTTCAGTGGTAGAATTCTCGCCTGCCACGCGGGAGGCCCGGGTTCGATTCCCGGCCCATGCA | 2450 | 1985 | 2568 | 2144 | 2654,842771 | 1,579227508 | 0,270871426 | 5,830173865 | 5,53697E-09 | 1,77565E-08 |
| HUS00001691 | MiRBase | MIMAT0000256 | Homo_sapiens | mature_miRNA | hsa-miR-181a-5p | hsa-miR-181a-5p | AACATTCAACGCTGTCGGTGAGT | 1735 | 1126 | 2815 | 1563 | 2231,240212 | 2,051267369 | 0,357764549 | 5,733568004 | 9,83396E-09 | 3,13205E-08 |
| HUS00042803 | MiRBase | MIMAT0022494 | Homo_sapiens | mature_miRNA | hsa-miR-5701 | hsa-miR-5701 | TTATTGTCACGTTCTGATT | 133 | 82 | 2 | 1 | 32,98645628 | -4,155715388 | 0,727891287 | -5,709252823 | 1,13473E-08 | 3,58946E-08 |
| HUS00104973 | MiRBase | MIMAT0000099 | Homo_sapiens | mature_miRNA | hsa-miR-101-3p | hsa-miR-101-3p | TACAGTACTGTGATAACTGAA | 18177 | 15282 | 1352 | 1520 | 6215,24655 | -1,965514957 | 0,347258229 | -5,660096126 | 1,51288E-08 | 4,75331E-08 |
| HUS00225878 | MiRBase | MIMAT0000418 | Homo_sapiens | mature_miRNA | hsa-miR-23b-3p | hsa-miR-23b-3p | ATCACATTGCCAGGGATTACC | 3286 | 2951 | 9261 | 3617 | 6142,27681 | 2,410384983 | 0,432984319 | 5,566910577 | 2,59295E-08 | 8,0921E-08 |
| HUS00082074 | Rfam | RF00221 | Homo_sapiens | snoRNA | SNORD43 | SNORD43 | CACAGATGATGAACTTATTGACGGGCGGACAGGAAACTGTG | 1215 | 2372 | 51 | 89 | 605,3535517 | -2,980808738 | 0,538091233 | -5,539597297 | 3,03168E-08 | 9,39821E-08 |
| HUS00366472 | GtRNA\|GtRNA\|GtRNA\|GtRNA\|GtRNA\|GtRNA\|GtRNA\|GtRNA\|GtRNA\|GtRNA\|GtRNA\|GtRNA\|GtRNA\|GtRNA\|GtRNA\|GtRNA\|GtRNA\|GtRNA\|GtRNA\|GtRNA\|GtRNA\|GtRNA | chr1\|chr1\|chr1\|chr1\|chr1\|chr12\|chr12\|chr12\|chr17\|chr6\|chr6\|chr1\|chr1\|chr1\|chr1\|chr1\|chr12\|chr12\|chr12\|chr17\|chr6\|chr6 | Homo_sapiens | tRNA | trna69-AspGTC | trna69-AspGTC | TCCTCGTTAGTATAGTGGTGAGTATCCCCGCCTGTCACGCGGGAGACCGGGGTTCGATTCCCCGACGGGGAG | 343 | 1670 | 8732 | 3983 | 5501,175488 | 3,731857582 | 0,681932486 | 5,472473681 | 4,43797E-08 | 1,36666E-07 |
| HUS00117945 | Ensembl | ENST00000516517 | Homo_sapiens | snoRNA | ENSG00000252326 | SNORD116-25 | GGATCGATGATGACTTTAAAATGGATCTCATCGGAATCTGAACAAAATGAGTGACCAAATCACTTCTGTGCCACTTCTGTGAGCTGAGGTCC | 1145 | 1595 | 129 | 109 | 512,994431 | -1,986066527 | 0,364873404 | -5,443166052 | 5,23418E-08 | 1,60125E-07 |
| HUS00131360 | Ensembl | ENST00000363753 | Homo_sapiens | snoRNA | ENSG00000200623 | SNORD18A | CAGTAGTGATGAAATTCCACTTCATTGGTCCGTGTTTCTGAACCACATGATTTTCTCGGATGTTCTGATGCT | 22760 | 30385 | 2344 | 2129 | 9890,529879 | -2,020225898 | 0,372433802 | -5,424389216 | 5,8153E-08 | 1,7674E-07 |
| HUS00099652 | MiRBase | MIMAT0004558 | Homo_sapiens | mature_miRNA | hsa-miR-181a-2-3p | hsa-miR-181a-2-3p | ACCACTGACCGTTGACTGTACC | 33 | 42 | 59 | 78 | 70,97211288 | 2,327674024 | 0,429258416 | 5,422547207 | 5,87557E-08 | 1,77412E-07 |
| HUS00240215 | MiRBase | MIMAT0006764 | Homo_sapiens | mature_miRNA | hsa-miR-320d | hsa-miR-320d | AAAAGCTGGGTTGAGAGGA | 209 | 249 | 662 | 260 | 442,0108749 | 2,366248387 | 0,438048247 | 5,401798553 | 6,5976E-08 | 1,97928E-07 |
| HUS00045199 | Rfam\|Ensembl | RF00604\|ENST00000408454 | Homo_sapiens | snoRNA | SNORD88 | SNORD88 | GGGACCCCGTGATGTCCAGCACTGGGCTCTGACTGCCCCTGAGGACACGGTGCACCCCGGGACCTTTGACATCCGGGGTTCTGAGGGGCCC | 155 | 71 | 2 | 3 | 35,37061939 | -3,620062545 | 0,67755715 | -5,342815059 | 9,15141E-08 | 2,72782E-07 |
| HUS00100472 | GtRNA\|GtRNA\|GtRNA\|GtRNA | chr16\|chr2\|chr16\|chr2 | Homo_sapiens | tRNA | trna34-GlyCCC | trna34-GlyCCC | GCGCCGCTGGTGTAGTGGTATCATGCAAGATTCCCATTCTTGCGACCCGGGTTCGATTCCCGGGCGGCGCA | 283 | 363 | 638 | 336 | 498,0060628 | 2,000543938 | 0,378124157 | 5,29070651 | 1,21845E-07 | 3,58594E-07 |
| HUS00069953 | Rfam | RF00001 | Homo_sapiens | rRNA | 5S_rRNA | 5S_rRNA | TATGTATACATGTGCCATGCCCGATCTCGTCTGATCTCGGAAGCTAAGCAGGGTCGGGCCTGGTTAGTACTTGGATGGGACACTTGGAAGTTTTCAAAG | 146722 | 76997 | 347652 | 198680 | 258968,1157 | 2,659768131 | 0,502614746 | 5,291862505 | 1,21077E-07 | 3,58594E-07 |
| HUS00135537 | Ensembl | ENST00000607707 | Homo_sapiens | snoRNA | ENSG00000271852 | SNORD11B | TGATGGCAATGATGATTTTTACACTTATTGTTGTTCACCTGATAACATAAATATGAGGGTGTTCAGTCACTACCTCATCTGATGCCATCA | 130 | 177 | 8 | 9 | 53,487983 | -2,564461581 | 0,486290151 | -5,273521535 | 1,33831E-07 | 3,88945E-07 |
| HUS00024323 | Ensembl | ENST00000362761 | Homo_sapiens | snoRNA | ENSG00000199631 | SNORD33 | GCGGCCGGTGATGAGAACTTCTCCCACTCACATTCGAGTTTCCCGACCATGAGATGACTCCACATGCACTACCATCTGAGGCCAC | 362 | 188 | 701 | 338 | 506,5251899 | 2,317087687 | 0,43930905 | 5,274390972 | 1,33198E-07 | 3,88945E-07 |
| HUS00341222 | MiRBase | MIMAT0004682 | Homo_sapiens | mature_miRNA | hsa-miR-361-3p | hsa-miR-361-3p | TCCCCCAGGTGTGATTCTGATTT | 1640 | 1656 | 1873 | 2205 | 2257,101438 | 1,806111608 | 0,342913769 | 5,266955638 | 1,38705E-07 | 4,00607E-07 |
| HUS00356655 | Ensembl | ENST00000364953 | Homo_sapiens | snoRNA | ENSG00000201823 | SNORD48 | AGTGATGATGACCCCAGGTAACTCTTGAGTGTGTCGCTGATGCCATCACCGCAGCGCTCTGACC | 765 | 767 | 7 | 34 | 247,7545575 | -3,350622108 | 0,638059876 | -5,251265961 | 1,51057E-07 | 4,33591E-07 |
| HUS00137080 | Ensembl | ENST00000387416 | Homo_sapiens | Mt_tRNA | ENSG00000210151 | MT-TS1 | GAAAAAGTCATGGAGGCCATGGGGTTGGCTTGAAACCAGCTTTGGGGGGTTCGATTCCTTCCTTTTTTG | 62 | 106 | 1 | 0 | 25,75328044 | -4,702011809 | 0,898947125 | -5,230576615 | 1,68982E-07 | 4,82066E-07 |
| HUS00136572 | MiRBase | MIMAT0004749 | Homo_sapiens | mature_miRNA | hsa-miR-424-3p | hsa-miR-424-3p | CAAAACGTGAGGCGCTGCTAT | 297 | 424 | 551 | 850 | 724,7862134 | 2,415225495 | 0,462066508 | 5,227008345 | 1,72275E-07 | 4,88462E-07 |
| HUS00010416 | MiRBase | MIMAT0000073 | Homo_sapiens | mature_miRNA | hsa-miR-19a-3p | hsa-miR-19a-3p | TGTGCAAATCTATGCAAAACTGA | 2152 | 1301 | 178 | 81 | 615,5240952 | -2,197640528 | 0,42271171 | -5,198910921 | 2,0046E-07 | 5,64931E-07 |
| HUS00247941 | GtRNA\|GtRNA | chr17\|chr17 | Homo_sapiens | tRNA | trna16-GlnTTG | trna16-GlnTTG | GGTCCCATGGTGTAATGGTTAGCACTCTGGACTTTGAATCCAGCGATCCGAGTTCAAATCTCGGTGGGACCT | 1626 | 765 | 100 | 66 | 420,4504885 | -2,265215307 | 0,435865221 | -5,197054497 | 2,02471E-07 | 5,67163E-07 |
| HUS00032315 | MiRBase | MIMAT0004571 | Homo_sapiens | mature_miRNA | hsa-miR-200b-5p | hsa-miR-200b-5p | CATCTTACTGGGCAGCATTGGA | 599 | 305 | 708 | 704 | 737,678752 | 2,122703714 | 0,409997705 | 5,17735511 | 2,25054E-07 | 6,26646E-07 |
| HUS00147295 | MiRBase | MIMAT0001341 | Homo_sapiens | mature_miRNA | hsa-miR-424-5p | hsa-miR-424-5p | CAGCAGCAATTCATGTTTTGAA | 987 | 917 | 139 | 93 | 380,7058995 | -1,524681232 | 0,299224594 | -5,095440888 | 3,47931E-07 | 9,63022E-07 |
| HUS00059657 | MiRBase | MIMAT0004602 | Homo_sapiens | mature_miRNA | hsa-miR-125a-3p | hsa-miR-125a-3p | ACAGGTGAGGTTCTTGGGAGCC | 67 | 76 | 98 | 159 | 134,7930008 | 2,317000926 | 0,455268447 | 5,089307065 | 3,59374E-07 | 9,88811E-07 |
| HUS00253412 | Ensembl | ENST00000365223 | Homo_sapiens | snoRNA | ENSG00000202093 | SNORD58C | TTGCTGTGATGACTATCTTAGGACACCTTTGGAATAACTATGAAAGAAAACTATTCTGAGCAAC | 6810 | 10637 | 557 | 717 | 3184,999025 | -2,177222866 | 0,430388952 | -5,05873316 | 4,22051E-07 | 1,15443E-06 |
| HUS00039155 | Ensembl | ENST00000363315 | Homo_sapiens | snoRNA | ENSG00000274091 | SNORD1C | CTGAGCTGAGGATGATTTAAAGTTATCCCTGTCTGAAATGGTATCTTTTGTGAGGAGGTCTGACTTGCTGAGGCTCAG | 103 | 63 | 127 | 89 | 115,0051684 | 1,84162497 | 0,364762325 | 5,048835484 | 4,44511E-07 | 1,20876E-06 |
| HUS00030026 | Ensembl | ENST00000459623 | Homo_sapiens | snoRNA | ENSG00000238862 | SNORD19B | TTTTGGTTGAAATATGATGAGTGTACAAAATCTTGATTTAAGTGAATGAAAAATTACAAGATCCAACTCTGATTTCAGCCAGAG | 5468 | 8213 | 122 | 366 | 2283,23964 | -3,012221082 | 0,602870087 | -4,996467974 | 5,83899E-07 | 1,57856E-06 |
| HUS00082419 | Ensembl | ENST00000384645 | Homo_sapiens | snoRNA | ENSG00000207375 | SNORD116-23 | GGATCGATGATGACCTCAATACATGCATTCCTTGGAAAGCTGAACAAAATGAGTGAAAACTCTATACCGTCGTCCTCGTCAAACTGAGGTCC | 24 | 40 | 51 | 53 | 54,32493403 | 2,139530841 | 0,431128131 | 4,962633348 | 6,95438E-07 | 1,86924E-06 |
| HUS00325118 | Ensembl | ENST00000458892 | Homo_sapiens | snoRNA | ENSG00000239043 | SNORD127 | TGGCAACTGTGATGAAAGATTTGGTCTGTATGTAATAGATTTTATTACTAAATGAGGACAACAGTCCCTCTAAACTGATGTTGCCA | 8065 | 16050 | 994 | 771 | 4398,294645 | -2,218582358 | 0,447263089 | -4,960351998 | 7,03656E-07 | 1,88046E-06 |
| HUS00176719 | MiRBase | MIMAT0000459 | Homo_sapiens | mature_miRNA | hsa-miR-193a-3p | hsa-miR-193a-3p | AACTGGCCTACAAAGTCCCAGT | 61 | 76 | 2 | 1 | 21,78631809 | -3,595131777 | 0,729833447 | -4,925961933 | 8,39463E-07 | 2,23057E-06 |
| HUS00324610 | MiRBase | MIMAT0005797 | Homo_sapiens | mature_miRNA | hsa-miR-1301-3p | hsa-miR-1301-3p | TTGCAGCTGCCTGGGAGTGACTTC | 278 | 178 | 280 | 239 | 287,542937 | 1,673365689 | 0,339844695 | 4,92391294 | 8,48306E-07 | 2,24126E-06 |
| HUS00181043 | Ensembl\|Ensembl | ENST00000428514\|ENST00000426867 | Homo_sapiens | snoRNA | ENSG00000235284 | SNORD62A | TCTCAGTGATGTAATTCCAATAGATCCTTCTGACCCTCCACTGTGGACTCAATAGCAGGGAGATGAAGAGGACAGTGACTGAGAGA | 1656 | 2877 | 51 | 129 | 766,3089387 | -2,8948144 | 0,588112769 | -4,922209743 | 8,55724E-07 | 2,24809E-06 |
| HUS00003948 | MiRBase | MIMAT0004681 | Homo_sapiens | mature_miRNA | hsa-miR-26a-2-3p | hsa-miR-26a-2-3p | CCTATTCTTGATTACTTGTTTC | 124 | 104 | 6 | 9 | 40,4924725 | -2,314456054 | 0,472187978 | -4,901556498 | 9,50803E-07 | 2,48384E-06 |
| HUS00159244 | Ensembl | ENST00000459473 | Homo_sapiens | snoRNA | ENSG00000239112 | SNORD123 | GGTGAAAATGATGAATTCTGGGGCGCTGATTCATGTGACTTGAAAAATGCCATCCATTTCCTGATTCACC | 466 | 291 | 43 | 23 | 139,0369073 | -1,973517915 | 0,406930933 | -4,84976136 | 1,2361E-06 | 3,2111E-06 |
| HUS00277903 | Rfam | RF00334 | Homo_sapiens | snoRNA | SNORA3 | SNORA3 | GCCGAGACTAGAGTCACATCCTGACACAACTCTTGTCCTGGTGTGCTAGAGTACTCGAAGAGAATCTACTGGTCTTGA | 1338 | 3259 | 140 | 130 | 814,4063166 | -2,486488043 | 0,513078946 | -4,846209462 | 1,25843E-06 | 3,25094E-06 |
| HUS00272420 | Ensembl | ENST00000365530 | Homo_sapiens | snoRNA | ENSG00000202400 | SNORD82 | AGCACAAATGATGAATAACAAAGGGACTTAATACTGAAACCTGATGTTACATTGTAGTGTGCTGATGTGC | 22750 | 49087 | 2211 | 2182 | 12783,11395 | -2,426698927 | 0,502755464 | -4,826797719 | 1,38746E-06 | 3,56447E-06 |
| HUS00031587 | Ensembl | ENST00000516014 | Homo_sapiens | rRNA | ENSG00000251823 | RNA5SP162 | GCCTGGTTAGTACTTGGATGGGAGACCGCCTGGGAATACCGGGT | 324 | 950 | 1399 | 2282 | 1818,903293 | 2,874036049 | 0,595990884 | 4,822281898 | 1,41925E-06 | 3,62611E-06 |
| HUS00194464 | Ensembl | ENST00000386347 | Homo_sapiens | Mt_tRNA | ENSG00000209082 | MT-TL1 | GTTAAGATGGCAGAGCCCGGTAATCGCATAAAACTTAAAACTTTACAGTCAGAGGTTCAATTCCTCTTCTTAACA | 193 | 362 | 20 | 16 | 99,2071643 | -2,364171988 | 0,491252229 | -4,812542006 | 1,49023E-06 | 3,78664E-06 |
| HUS00196115 | Ensembl | ENST00000516006 | Homo_sapiens | snoRNA | ENSG00000251815 | SNORD116-26 | GGATCGATGATGACTATAAAAAAAATGGATCTCATCGGAATCTGAACAAAATGAGTGACCAAATCATTTCTGTGCCACTTCTGTGAGCTGAGGTCC | 1363 | 2586 | 173 | 135 | 727,7277159 | -2,129152595 | 0,443879425 | -4,796691347 | 1,61308E-06 | 4,07653E-06 |
| HUS00210876 | MiRBase | MIMAT0022706 | Homo_sapiens | mature_miRNA | hsa-miR-561-5p | hsa-miR-561-5p | ATCAAGGATCTTAAACTTTGCC | 102 | 85 | 7 | 5 | 32,83726797 | -2,35693711 | 0,492901471 | -4,781761162 | 1,73766E-06 | 4,36763E-06 |
| HUS00172572 | Ensembl\|Ensembl | ENST00000516733\|ENST00000629038 | Homo_sapiens | snoRNA | ENSG00000252542 | SNORD36C | TGCCAATGATGGTTAAGAATTTCTTCACCTGAATAAACCATGTGGTCAGCATTGCATCTGAGGCAA | 232 | 249 | 17 | 24 | 89,86803982 | -1,968087782 | 0,412476973 | -4,771388252 | 1,8296E-06 | 4,57401E-06 |
| HUS00293488 | MiRBase | MIMAT0004495 | Homo_sapiens | mature_miRNA | hsa-miR-22-5p | hsa-miR-22-5p | AGTTCTTCAGTGGCAAGCTTTA | 272 | 188 | 469 | 204 | 342,1708457 | 1,952639732 | 0,412893269 | 4,729163393 | 2,25447E-06 | 5,60603E-06 |
| HUS00151397 | MiRBase | MIMAT0000424 | Homo_sapiens | mature_miRNA | hsa-miR-128-3p | hsa-miR-128-3p | TCACAGTGAACCGGTCTCTTT | 6527 | 5211 | 6162 | 6556 | 7213,266308 | 1,617013376 | 0,343705338 | 4,704650173 | 2,54302E-06 | 6,28991E-06 |
| HUS00159697 | Rfam | RF00150 | Homo_sapiens | snoRNA | SNORD42 | SNORD42 | GGCTAATGATGGAAAAATCATTATTGGAAAAGAATGACATGAACAAAGGAACCACTGAAGTG | 1677 | 1060 | 131 | 142 | 522,0135515 | -1,743695289 | 0,376196041 | -4,63507081 | 3,56815E-06 | 8,77879E-06 |
| HUS00076519 | Ensembl | ENST00000459584 | Homo_sapiens | snoRNA | ENSG00000238649 | SNORD42A | GGGCTAATGATGGAAAAATCATTATTGGAAAAGAATGACATGAACAAAGGAACCACTGAAGTGC | 1669 | 1057 | 131 | 142 | 520,4049965 | -1,738186125 | 0,3757702 | -4,625662501 | 3,73403E-06 | 9,13854E-06 |
| HUS00001657 | MiRBase | MIMAT0003247 | Homo_sapiens | mature_miRNA | hsa-miR-582-5p | hsa-miR-582-5p | TTACAGTTGTTCAACCAGTTACT | 93 | 167 | 9 | 6 | 45,58104547 | -2,504936065 | 0,542009806 | -4,621569644 | 3,80847E-06 | 9,22365E-06 |
| HUS00284741 | MiRBase | MIMAT0000681 | Homo_sapiens | mature_miRNA | hsa-miR-29c-3p | hsa-miR-29c-3p | TAGCACCATTTGAAATCGGTTA | 434 | 262 | 41 | 22 | 128,7185744 | -1,917513523 | 0,414856442 | -4,622113405 | 3,7985E-06 | 9,22365E-06 |
| HUS00114818 | MiRBase | MIMAT0003239 | Homo_sapiens | mature_miRNA | hsa-miR-574-3p | hsa-miR-574-3p | CACGCTCATGCACACACCCACA | 165 | 143 | 498 | 140 | 299,8977416 | 2,362580052 | 0,511540989 | 4,618554723 | 3,86422E-06 | 9,31017E-06 |
| HUS00090506 | Ensembl | ENST00000364805 | Homo_sapiens | snoRNA | ENSG00000201675 | SNORD32A | GAGGTCAGTGATGAGCAACATTCACCATCTTTCGTTTGAGTCTCACGGCCATGAGATCAACCCCATGCACCGCTCTGAGACCTG | 13020 | 10196 | 1656 | 734 | 4421,333559 | -1,775869509 | 0,391181546 | -4,539757891 | 5,63189E-06 | 1,34991E-05 |
| HUS00190080 | Ensembl | ENST00000413522 | Homo_sapiens | snoRNA | ENSG00000229686 | SNORD56 | CCACAATGATGGCAATATTTTTCGTCAACAGCAGTTCACCTAGTGAGTGTTGAGACTCTGGGTCTGAGTGA | 1436 | 1517 | 214 | 95 | 567,3968962 | -1,758763867 | 0,391237837 | -4,495382861 | 6,94449E-06 | 1,65599E-05 |
| HUS00012939 | MiRBase | MIMAT0004678 | Homo_sapiens | mature_miRNA | hsa-miR-99b-3p | hsa-miR-99b-3p | CAAGCTCGTGTCTGTGGGTCCG | 642 | 607 | 644 | 972 | 896,1907812 | 1,8733333 | 0,418010707 | 4,481543821 | 7,4105E-06 | 1,7581E-05 |
| HUS00327327 | MiRBase | MIMAT0001340 | Homo_sapiens | mature_miRNA | hsa-miR-423-3p | hsa-miR-423-3p | AGCTCGGTCTGAGGCCCCTCAGT | 8740 | 3680 | 10053 | 10122 | 10466,98995 | 2,158762303 | 0,484353034 | 4,457001712 | 8,31139E-06 | 1,96183E-05 |
| HUS00184496 | Ensembl | ENST00000384516 | Homo_sapiens | snoRNA | ENSG00000207245 | SNORD116-29 | GGATCGATGATGACTTAAAAAAATGGAAACCTTGGAAATCTGAACAAAATGAGTGACCAAGACACTTCTGTGAGCTGAGGTCC | 95 | 138 | 138 | 179 | 173,0874365 | 1,913285156 | 0,429568666 | 4,453968148 | 8,42976E-06 | 1,97972E-05 |
| HUS00016157 | MiRBase | MIMAT0000090 | Homo_sapiens | mature_miRNA | hsa-miR-32-5p | hsa-miR-32-5p | TATTGCACATTACTAAGTTGCA | 3352 | 2429 | 419 | 192 | 1106,408056 | -1,732647807 | 0,390158637 | -4,440880304 | 8,95916E-06 | 2,09347E-05 |
| HUS00061946 | GtRNA\|GtRNA\|GtRNA\|GtRNA\|GtRNA\|GtRNA\|GtRNA\|GtRNA\|GtRNA\|GtRNA\|GtRNA\|GtRNA | chr1\|chr1\|chr10\|chr13\|chr17\|chr19\|chr1\|chr1\|chr10\|chr13\|chr17\|chr19 | Homo_sapiens | tRNA | trna26-AsnGTT | trna26-AsnGTT | GTCTCTGTGGCGCAATCGGTTAGCGCGTTCGGCTGTTAACCGAAAGGTTGGTGGTTCGAGCCCACCCAGGGACG | 216 | 117 | 512 | 156 | 316,0707759 | 2,330329424 | 0,525920613 | 4,430952823 | 9,38176E-06 | 2,18126E-05 |
| HUS00095312 | Ensembl | ENST00000458893 | Homo_sapiens | snoRNA | ENSG00000238423 | SNORD42B | GTGCATATGATGGAAAAGTTTTAATCTCCTGACACTTGTGATGTCTTCAAAGGAACCACTGATGCAC | 6822 | 6020 | 1019 | 479 | 2524,570315 | -1,604936525 | 0,362676185 | -4,425260306 | 9,63261E-06 | 2,22844E-05 |
| HUS00040038 | GtRNA\|GtRNA | chr19\|chr19 | Homo_sapiens | tRNA | trna8-SeC(e)TCA | trna8-SeC(e)TCA | GCCCGGATGATCCTCAGTGGTCTGGGGTGCAGGCTTCAAACCTGTAGCTGTCTAGCGACAGAGTGGTTCAATTCCACCTTTCGGGC | 172 | 65 | 321 | 136 | 220,4906341 | 2,309153894 | 0,524212127 | 4,404999002 | 1,05784E-05 | 2,43514E-05 |
| HUS00146483 | MiRBase | MIMAT0000728 | Homo_sapiens | mature_miRNA | hsa-miR-375 | hsa-miR-375 | TTTGTTCGTTCGGCTCGCGTGA | 17254 | 16143 | 15592 | 19756 | 20345,98613 | 1,589559167 | 0,367249031 | 4,328286887 | 1,50274E-05 | 3,44223E-05 |
| HUS00268102 | GtRNA\|GtRNA | chr14\|chr14 | Homo_sapiens | tRNA | trna20-ThrTGT | trna20-ThrTGT | GGCTCCATAGCTCAGGGGTTAGAGCACTGGTCTTGTAAACCAGGGGTCGCGAGTTCAAATCTCGCTGGGGCCT | 121 | 122 | 16 | 6 | 45,17295425 | -1,922961806 | 0,444664521 | -4,324522675 | 1,52862E-05 | 3,48437E-05 |
| HUS00079963 | MiRBase | MIMAT0002174 | Homo_sapiens | mature_miRNA | hsa-miR-484 | hsa-miR-484 | TCAGGCTCAGTCCCCTCCCGAT | 683 | 289 | 1089 | 509 | 795,8497242 | 2,108629559 | 0,488477182 | 4,316741161 | 1,5835E-05 | 3,59183E-05 |
| HUS00256189 | MiRBase | MIMAT0004700 | Homo_sapiens | mature_miRNA | hsa-miR-331-5p | hsa-miR-331-5p | CTAGGTATGGTCCCAGGGATCC | 119 | 124 | 338 | 99 | 210,6681387 | 2,168837803 | 0,504335286 | 4,300388775 | 1,70499E-05 | 3,84864E-05 |
| HUS00182106 | MiRBase | MIMAT0004597 | Homo_sapiens | mature_miRNA | hsa-miR-140-3p | hsa-miR-140-3p | TACCACAGGGTAGAACCACGG | 467 | 359 | 403 | 305 | 420,6312975 | 1,263065168 | 0,295257928 | 4,277836597 | 1,88718E-05 | 4,23933E-05 |
| HUS00144940 | MiRBase | MIMAT0000275 | Homo_sapiens | mature_miRNA | hsa-miR-218-5p | hsa-miR-218-5p | TTGTGCTTGATCTAACCATGT | 7728 | 8824 | 1340 | 654 | 3295,897588 | -1,564083996 | 0,366705826 | -4,265228104 | 1,99698E-05 | 4,4644E-05 |
| HUS00340266 | Ensembl | ENST00000410856 | Homo_sapiens | snRNA | ENSG00000222788 | RNU2-38P | ATCGCTTCTCGGCCTTTTGGCTAAGATCAAGTGTAGTATCTGTTCTTATCAGCTTAAATAATCAGGATGGAAATTCAAT | 1340 | 2513 | 4083 | 1918 | 3037,543094 | 1,99684417 | 0,46839286 | 4,263182344 | 2,01536E-05 | 4,48393E-05 |
| HUS00065412 | MiRBase | MIMAT0000086 | Homo_sapiens | mature_miRNA | hsa-miR-29a-3p | hsa-miR-29a-3p | TAGCACCATCTGAAATCGGTTA | 81062 | 47342 | 9682 | 6001 | 25463,66875 | -1,505746488 | 0,358196905 | -4,203683688 | 2,62606E-05 | 5,78729E-05 |
| HUS00330309 | MiRBase | MIMAT0000244 | Homo_sapiens | mature_miRNA | hsa-miR-30c-5p | hsa-miR-30c-5p | TGTAAACATCCTACACTCTCAGC | 3742 | 2918 | 2912 | 2346 | 3210,879201 | 1,153677803 | 0,27443934 | 4,203762493 | 2,62514E-05 | 5,78729E-05 |
| HUS00186714 | Ensembl | ENST00000408813 | Homo_sapiens | snoRNA | ENSG00000221740 | SNORD93 | TGGCCAAGGATGAGAACTCTAATCTGATTTTATGTGCTTCTGCTGTGATGGATTAAAGGATTTACCTGAGGCCA | 256 | 173 | 23 | 23 | 83,19565256 | -1,653878318 | 0,396050654 | -4,175926245 | 2,96776E-05 | 6,50947E-05 |
| HUS00334964 | Rfam | RF00218 | Homo_sapiens | snoRNA | SNORD46 | SNORD46 | AAGTAGGGTGATGAAAAAGAATCCTTAGGCGTGGTTGTGGCCGTCTTGGTCACCTGTGTGCCACTT | 32563 | 31419 | 5513 | 3663 | 13376,16642 | -1,295630103 | 0,311239735 | -4,162804286 | 3,14363E-05 | 6,86285E-05 |
| HUS00183703 | MiRBase | MIMAT0004953 | Homo_sapiens | mature_miRNA | hsa-miR-873-5p | hsa-miR-873-5p | GCAGGAACTTGTGAGTCTCCT | 219 | 228 | 201 | 195 | 236,1232602 | 1,317904382 | 0,318031701 | 4,143940298 | 3,41389E-05 | 7,41802E-05 |
| HUS00300555 | MiRBase | MIMAT0017990 | Homo_sapiens | mature_miRNA | hsa-miR-3613-5p | hsa-miR-3613-5p | TGTTGTACTTTTTTTTTTGTTC | 669 | 449 | 61 | 68 | 221,0722036 | -1,541500032 | 0,375704491 | -4,102958757 | 4,079E-05 | 8,82202E-05 |
| HUS00260724 | MiRBase | MIMAT0000088 | Homo_sapiens | mature_miRNA | hsa-miR-30a-3p | hsa-miR-30a-3p | CTTTCAGTCGGATGTTTGCAGC | 494 | 390 | 391 | 447 | 493,2962393 | 1,432290928 | 0,349478994 | 4,098360568 | 4,16087E-05 | 8,95742E-05 |
| HUS00122885 | Ensembl | ENST00000516438 | Homo_sapiens | snRNA | ENSG00000252247 | RNU6-70P | CTGCTTCGGCAGCACATATACTAAAATTGGAACGATACAGAGAAGATTAGCGTGGCCCCT | 70 | 70 | 67 | 115 | 101,4543989 | 1,870317688 | 0,460722856 | 4,059528764 | 4,91719E-05 | 0,000105368 |
| HUS00069196 | MiRBase | MIMAT0000691 | Homo_sapiens | mature_miRNA | hsa-miR-130b-3p | hsa-miR-130b-3p | CAGTGCAATGATGAAAGGGCAT | 137 | 118 | 293 | 91 | 191,5829856 | 1,939904 | 0,487102286 | 3,982539305 | 6,81828E-05 | 0,000145436 |
| HUS00246844 | MiRBase | MIMAT0000071 | Homo_sapiens | mature_miRNA | hsa-miR-17-3p | hsa-miR-17-3p | ACTGCAGTGAAGGCACTTGTAG | 211 | 130 | 23 | 13 | 65,2488548 | -1,692094811 | 0,429522034 | -3,939483138 | 8,16573E-05 | 0,000173382 |
| HUS00187789 | Ensembl | ENST00000363660 | Homo_sapiens | snoRNA | ENSG00000200530 | SNORD35B | GGCAGATGATGTTTGTTTTCACGATGGTCTTCAGATGCCCACGTGGGCACTGCTGAGAAAGCCACTTGGTAAAACTGATGCCGGAAA | 447 | 177 | 24 | 26 | 112,8688817 | -1,999564273 | 0,51164988 | -3,908071417 | 9,30358E-05 | 0,000196644 |
| HUS00151059 | Ensembl | ENST00000384567 | Homo_sapiens | snoRNA | ENSG00000207297 | SNORD7 | ATGCGATGATGAGTGAAGTAGAGCCTGACCTGGTATTGCCATTGCTTCACTGTTGGCTTTGACCAGGGTATGATCTCTTAATCTTCTCTCTGAGCTG | 235 | 304 | 23 | 33 | 105,4654706 | -1,683331827 | 0,434167918 | -3,877144662 | 0,00010569 | 0,000222378 |
| HUS00135422 | MiRBase | MIMAT0000255 | Homo_sapiens | mature_miRNA | hsa-miR-34a-5p | hsa-miR-34a-5p | TGGCAGTGTCTTAGCTGGTTGT | 183 | 252 | 31 | 19 | 86,18441737 | -1,598586528 | 0,412489876 | -3,875456395 | 0,000106425 | 0,000222917 |
| HUS00209789 | Ensembl | ENST00000517057 | Homo_sapiens | rRNA | ENSG00000252866 | RNA5SP243 | AACGCGCCCGATCTCGTCTGATCTCGGAAGCTAAG | 698 | 732 | 612 | 2275 | 1540,012567 | 2,447858979 | 0,6341014 | 3,860358893 | 0,000113221 | 0,000236088 |
| HUS00333348 | MiRBase | MIMAT0000091 | Homo_sapiens | mature_miRNA | hsa-miR-33a-5p | hsa-miR-33a-5p | GTGCATTGTAGTTGCATTGCA | 201 | 162 | 26 | 21 | 73,79812051 | -1,410840141 | 0,365820089 | -3,856650261 | 0,000114951 | 0,000238627 |
| HUS00042481 | Ensembl | ENST00000408189 | Homo_sapiens | snoRNA | ENSG00000221116 | SNORD110 | TTGCAGTGATGACTTGCGAATCAAATCTGTCAATCCCCTGAGTGCAATCACTGATGTCTCCATGTCTCTGAGCAA | 686 | 176 | 824 | 667 | 755,5639847 | 2,199726353 | 0,570979653 | 3,852547708 | 0,000116895 | 0,000241583 |
| HUS00199023 | MiRBase | MIMAT0000245 | Homo_sapiens | mature_miRNA | hsa-miR-30d-5p | hsa-miR-30d-5p | TGTAAACATCCCCGACTGGAAG | 38113 | 27963 | 30505 | 20239 | 30983,30551 | 1,097855319 | 0,285780061 | 3,841609233 | 0,00012223 | 0,000251492 |
| HUS00207745 | MiRBase | MIMAT0004555 | Homo_sapiens | mature_miRNA | hsa-miR-10a-3p | hsa-miR-10a-3p | CAAATTCGTATCTAGGGGAATA | 324 | 301 | 574 | 208 | 408,4785619 | 1,702590395 | 0,443892402 | 3,835592561 | 0,000125262 | 0,000256594 |
| HUS00079582 | Ensembl | ENST00000391150 | Homo_sapiens | snoRNA | ENSG00000212452 | SNORD69 | AATGTGAAGCAAATGATGATAAACTGGATCTGACTGACTGTGCTGAGTCTGTTCAATCCAACCCTGAGCTTCATGTT | 42795 | 33827 | 3933 | 5051 | 15287,96009 | -1,50167935 | 0,394087051 | -3,810527003 | 0,000138671 | 0,000282816 |
| HUS00286096 | Ensembl | ENST00000459342 | Homo_sapiens | snoRNA | ENSG00000239195 | SNORD5 | GTTCAGATGATGAATTTAACTGTTCAACTGCTGAATGATAACGGGCATGAACTAAAACTTAATTCTGACAGAG | 46435 | 17057 | 2470 | 2615 | 11467,34073 | -1,988566267 | 0,523056773 | -3,80181726 | 0,000143639 | 0,000291668 |
| HUS00339081 | MiRBase | MIMAT0005951 | Homo_sapiens | mature_miRNA | hsa-miR-1307-3p | hsa-miR-1307-3p | ACTCGGCGTGGCGTCGGTCGTG | 1007 | 523 | 900 | 645 | 873,3312796 | 1,486250453 | 0,391437681 | 3,796901846 | 0,000146516 | 0,000296217 |
| HUS00124785 | MiRBase | MIMAT0004592 | Homo_sapiens | mature_miRNA | hsa-miR-125b-1-3p | hsa-miR-125b-1-3p | ACGGGTTAGGCTCTTGGGAGCT | 1102 | 1382 | 1089 | 1060 | 1291,83075 | 1,277276362 | 0,337395851 | 3,785690776 | 0,000153282 | 0,000308555 |
| HUS00213960 | MiRBase | MIMAT0000693 | Homo_sapiens | mature_miRNA | hsa-miR-30e-3p | hsa-miR-30e-3p | CTTTCAGTCGGATGTTTACAGC | 949 | 658 | 693 | 613 | 793,1548065 | 1,200956043 | 0,320533578 | 3,746740206 | 0,000179147 | 0,000359067 |
| HUS00286360 | MiRBase | MIMAT0004518 | Homo_sapiens | mature_miRNA | hsa-miR-16-2-3p | hsa-miR-16-2-3p | CCAATATTACTGTGCTGCTTTA | 364 | 249 | 264 | 253 | 311,6387693 | 1,257598483 | 0,336506095 | 3,737223489 | 0,000186064 | 0,000371329 |
| HUS00235504 | Rfam\|GtRNA\|GtRNA | RF00005\|chr18\|chr18 | Homo_sapiens | tRNA | trna4-LysCTT | trna4-LysCTT | GACGAGCTAGCTCAGTCGGTAGAGCATGGGACTCTTAATCCCAGGGTCGTGGGTTTGAGCCCCATGTTGGGCA | 121 | 291 | 223 | 609 | 439,8601992 | 2,398812502 | 0,647046598 | 3,707325734 | 0,000209459 | 0,000416234 |
| HUS00301794 | Ensembl | ENST00000365012 | Homo_sapiens | snoRNA | ENSG00000201882 | snoU2-30 | GGGCAATGATGTAAAGGTTTTACGACTGACCTTTGTAACTATGAAGTTTTCTACACTTGACCTGAGCTCA | 95 | 89 | 12 | 9 | 36,26541827 | -1,581808525 | 0,426861895 | -3,705668141 | 0,000210834 | 0,000417183 |
| HUS00200246 | MiRBase | MIMAT0004801 | Homo_sapiens | mature_miRNA | hsa-miR-590-3p | hsa-miR-590-3p | TAATTTTATGTATAAGCTAGT | 285 | 283 | 48 | 16 | 110,4838374 | -1,645483797 | 0,444747033 | -3,699819616 | 0,000215753 | 0,000425106 |
| HUS00097169 | MiRBase | MIMAT0000250 | Homo_sapiens | mature_miRNA | hsa-miR-139-5p | hsa-miR-139-5p | TCTACAGTGCACGTGTCTCCAGT | 210 | 330 | 252 | 327 | 333,5131114 | 1,576247076 | 0,44023374 | 3,580477674 | 0,000342967 | 0,000672909 |
| HUS00022361 | MiRBase | MIMAT0001536 | Homo_sapiens | mature_miRNA | hsa-miR-429 | hsa-miR-429 | TAATACTGTCTGGTAAAACCGT | 63433 | 40568 | 9584 | 5870 | 21793,68179 | -1,23453973 | 0,345830984 | -3,569777689 | 0,000357284 | 0,000698055 |
| HUS00004089 | Ensembl | ENST00000387342 | Homo_sapiens | Mt_tRNA | ENSG00000210077 | MT-TV | CAGAGTGTAGCTTAACACAAAGCACCCAACTTACACTTAGGAGATTTCAACTTAACTTGACCGCTCTGA | 1005 | 1402 | 200 | 78 | 474,569351 | -1,622851278 | 0,455359972 | -3,563886544 | 0,000365404 | 0,000710932 |
| HUS00072747 | MiRBase | MIMAT0004775 | Homo_sapiens | mature_miRNA | hsa-miR-502-3p | hsa-miR-502-3p | AATGCACCTGGGCAAGGATTCA | 930 | 620 | 1089 | 492 | 874,5124936 | 1,451494483 | 0,407619471 | 3,560905668 | 0,000369578 | 0,000716057 |
| HUS00116405 | GtRNA\|GtRNA | chr12\|chr12 | Homo_sapiens | tRNA | trna8-AlaTGC | trna8-AlaTGC | GGGGATGTAGCTCAGTGGTAGAGCGCATGCTTTGCACGTATGAGGCCCCGGGTTCAATCCCCGGCATCTCCA | 89 | 84 | 67 | 183 | 139,1378487 | 2,011738174 | 0,569900314 | 3,529982565 | 0,000415587 | 0,000801859 |
| HUS00180924 | MiRBase | MIMAT0000068 | Homo_sapiens | mature_miRNA | hsa-miR-15a-5p | hsa-miR-15a-5p | TAGCAGCACATAATGGTTTGTG | 231 | 161 | 34 | 17 | 78,98669218 | -1,417119828 | 0,402025415 | -3,524950849 | 0,000423561 | 0,000810519 |
| HUS00199736 | MiRBase | MIMAT0003339 | Homo_sapiens | mature_miRNA | hsa-miR-421 | hsa-miR-421 | ATCAACAGACATTAATTGGGCGC | 639 | 626 | 660 | 371 | 614,9127221 | 1,154941317 | 0,327641422 | 3,525016193 | 0,000423457 | 0,000810519 |
| HUS00089784 | Rfam\|Ensembl | RF00054\|ENST00000365607 | Homo_sapiens | snoRNA | SNORD25 | SNORD25 | CTTCCTATGATGAGGACCTTTTCACAGACCTGTACTGAGCTCCGTGAGGATAAATAACTCTGAGGAGAT | 18601 | 9255 | 1673 | 1670 | 5527,811291 | -1,471202619 | 0,421188193 | -3,492981621 | 0,000477659 | 0,000910293 |
| HUS00277420 | Ensembl | ENST00000384304 | Homo_sapiens | snoRNA | ENSG00000207031 | SNORD59A | CCTTCTATGATGATTTTATCAAAATGACTTTCGTTCTTCTGAGTTTGCTGAAGCCACATTTAGGTACTGAGAAGG | 8741 | 10507 | 1363 | 1486 | 4112,737003 | -1,209032889 | 0,348396941 | -3,470274125 | 0,000519927 | 0,000986801 |
| HUS00129468 | MiRBase | MIMAT0000261 | Homo_sapiens | mature_miRNA | hsa-miR-183-5p | hsa-miR-183-5p | TATGGCACTGGTAGAATTCACT | 47277 | 52306 | 35978 | 35065 | 45287,64227 | 1,013307114 | 0,294013513 | 3,446464429 | 0,000567973 | 0,001073608 |
| HUS00215288 | MiRBase | MIMAT0000259 | Homo_sapiens | mature_miRNA | hsa-miR-182-5p | hsa-miR-182-5p | TTTGGCAATGGTAGAACTCACACT | 73174 | 81857 | 58527 | 51313 | 69872,44388 | 0,994185918 | 0,288821079 | 3,442220778 | 0,000576959 | 0,001086178 |
| HUS00085841 | MiRBase | MIMAT0005949 | Homo_sapiens | mature_miRNA | hsa-miR-664a-3p | hsa-miR-664a-3p | TATTCATTTATCCCCAGCCTACA | 58 | 136 | 163 | 88 | 132,9847561 | 1,735613824 | 0,508320926 | 3,414405616 | 0,000639213 | 0,001198525 |
| HUS00155377 | Ensembl | ENST00000459124 | Homo_sapiens | snoRNA | ENSG00000238317 | SNORD11 | GTGTTCAATGATGATTTCTATTTGTTTGCCTGATTTCCTTTTGGATAATGAAGGCATCTTTAGTCACTACCTCTTCTGAGACAC | 338 | 216 | 49 | 26 | 112,7811855 | -1,35971779 | 0,39953841 | -3,403221706 | 0,000665962 | 0,001243664 |
| HUS00154150 | MiRBase | MIMAT0003389 | Homo_sapiens | mature_miRNA | hsa-miR-542-3p | hsa-miR-542-3p | TGTGACAGATTGATAACTGAAA | 1842 | 2362 | 335 | 323 | 912,3995552 | -1,143732602 | 0,346853466 | -3,297451848 | 0,000975664 | 0,001814735 |
| HUS00351381 | MiRBase | MIMAT0003321 | Homo_sapiens | mature_miRNA | hsa-miR-651-5p | hsa-miR-651-5p | TTTAGGATAAGCTTGACTTTTG | 1215 | 1512 | 266 | 189 | 599,9195035 | -1,080007687 | 0,33027044 | -3,270070695 | 0,001075206 | 0,001991916 |
| HUS00217438 | Ensembl | ENST00000384706 | Homo_sapiens | snoRNA | ENSG00000274544 | SNORD28 | GTCAGATGATTTGAATTGATAAGCTGATGTTCTGTGAGGTACAAAAGTTAATAGCATGTTAGAGTTCTGATGGCA | 100 | 117 | 80 | 153 | 136,2588139 | 1,601081981 | 0,491382926 | 3,258318305 | 0,001120746 | 0,002068044 |
| HUS00226723 | MiRBase | MIMAT0022724 | Homo_sapiens | mature_miRNA | hsa-miR-1277-5p | hsa-miR-1277-5p | AAATATATATATATATGTACGTAT | 112 | 78 | 17 | 7 | 37,88098828 | -1,447355857 | 0,449220876 | -3,221924747 | 0,001273326 | 0,002340302 |
| HUS00236686 | Ensembl | ENST00000383903 | Homo_sapiens | snoRNA | ENSG00000206630 | SNORD60 | AGTCTGTGATGAATTGCTTTGACTTCTGACACCTCGTATGAAAACTGCACGTGCAGTCTGATTATTTAGCAAGACTGAGGCTT | 5735 | 6796 | 1354 | 895 | 2816,793921 | -0,984227334 | 0,30913905 | -3,183769032 | 0,00145371 | 0,002661319 |
| HUS00027911 | MiRBase | MIMAT0003249 | Homo_sapiens | mature_miRNA | hsa-miR-584-5p | hsa-miR-584-5p | TTATGGTTTGCCTGGGACTGAG | 9526 | 8353 | 6951 | 5185 | 7765,517576 | 0,92630013 | 0,291980126 | 3,17247664 | 0,001511447 | 0,002756168 |
| HUS00207528 | Ensembl | ENST00000448188 | Homo_sapiens | snoRNA | ENSG00000226572 | SNORD57 | TGGAGGTGATGAACTGTCTGAGCCTGACCTTGTAGAATGGAGGCAAAAAAACTGATTTAATGAGCCTGATCC | 2638 | 3240 | 2124 | 2524 | 2894,390016 | 1,163989353 | 0,367547648 | 3,166907363 | 0,001540694 | 0,002798526 |
| HUS00139855 | Ensembl\|Ensembl | ENST00000362874\|ENST00000625314 | Homo_sapiens | snoRNA | ENSG00000199744 | SNORD36A | TTGCAATGATGTGAATCTCTCACTGAATTCAACCTTGAAGTGCGAATCCATGAGCTTTTTAACCCTGAGCAAT | 931 | 790 | 72 | 132 | 346,6134343 | -1,449927332 | 0,462121875 | -3,137543169 | 0,001703702 | 0,003082573 |
| HUS00310422 | MiRBase | MIMAT0002821 | Homo_sapiens | mature_miRNA | hsa-miR-181d-5p | hsa-miR-181d-5p | AACATTCATTGTTGTCGGTGGGT | 2309 | 2093 | 2292 | 1143 | 2065,511005 | 1,082503954 | 0,347490701 | 3,11520265 | 0,001838186 | 0,003313009 |
| HUS00318268 | Rfam | RF00069 | Homo_sapiens | snoRNA | SNORD24 | SNORD24 | TGCAGATGATGTAAAAGAATATTTGCTATCTGAGAGATGGTGATGACATTTTA | 1991 | 502 | 147 | 77 | 454,3547227 | -1,851112597 | 0,595474301 | -3,108635577 | 0,001879534 | 0,003374453 |
| HUS00335130 | MiRBase | MIMAT0000439 | Homo_sapiens | mature_miRNA | hsa-miR-153-3p | hsa-miR-153-3p | TTGCATAGTCACAAAAGTGATC | 90 | 61 | 13 | 6 | 30,111477 | -1,442202656 | 0,464109109 | -3,107464661 | 0,001886995 | 0,003374819 |
| HUS00327920 | MiRBase | MIMAT0004799 | Homo_sapiens | mature_miRNA | hsa-miR-589-5p | hsa-miR-589-5p | TGAGAACCACGTCTGCTCTGAG | 162 | 181 | 132 | 112 | 154,7925474 | 0,993276756 | 0,320349686 | 3,10060162 | 0,001931279 | 0,003440785 |
| HUS00267196 | Ensembl\|Ensembl\|Ensembl\|Ensembl\|Ensembl | ENST00000364884\|ENST00000579069\|ENST00000582902\|ENST00000583619\|ENST00000583541 | Homo_sapiens | snoRNA | ENSG00000201754 | SNORD52 | GGGAATGATGATTTCACAGACTAGAGTCTCCGATGCTGGTCATGATGTCAAAACTAAGTTCTGACTC | 7211 | 2045 | 605 | 322 | 1728,968408 | -1,720038646 | 0,560215216 | -3,070317616 | 0,002138312 | 0,003795096 |
| HUS00348792 | MiRBase | MIMAT0005900 | Homo_sapiens | mature_miRNA | hsa-miR-1248 | hsa-miR-1248 | ACCTTCTTGTATAAGCACTGTGCTAAA | 79 | 61 | 13 | 5 | 28,06117111 | -1,418638957 | 0,463144396 | -3,063059747 | 0,002190863 | 0,003873579 |
| HUS00117855 | MiRBase | MIMAT0004494 | Homo_sapiens | mature_miRNA | hsa-miR-21-3p | hsa-miR-21-3p | CAACACCAGTCGATGGGCTGT | 9270 | 8041 | 763 | 1368 | 3521,688046 | -1,395927723 | 0,456415937 | -3,058455261 | 0,002224813 | 0,003918705 |
| HUS00208903 | Ensembl | ENST00000607313 | Homo_sapiens | snoRNA | ENSG00000271982 | SNORD58B | CTGCGATGATGGCATTTCTTAGGACACCTTTGGATTAATAATGAAAACAACTACTCTCTGAGCAGC | 6256 | 9229 | 731 | 1196 | 3182,478879 | -1,40119908 | 0,470281805 | -2,979488179 | 0,002887304 | 0,005066401 |
| HUS00077824 | MiRBase | MIMAT0000425 | Homo_sapiens | mature_miRNA | hsa-miR-130a-3p | hsa-miR-130a-3p | CAGTGCAATGTTAAAAGGGCAT | 232 | 239 | 338 | 117 | 253,4012387 | 1,333617437 | 0,457446232 | 2,915353422 | 0,00355286 | 0,006210826 |
| HUS00326588 | MiRBase | MIMAT0004945 | Homo_sapiens | mature_miRNA | hsa-miR-744-5p | hsa-miR-744-5p | TGCGGGGCTAGGGCTAACAGCA | 1257 | 913 | 722 | 860 | 1006,821516 | 1,065795929 | 0,366361933 | 2,909133925 | 0,003624316 | 0,00631201 |
| HUS00133065 | PiRNA | DQ593049.1 | Homo_sapiens | piRNA | piR-33161 | piR-33161 | CCGGCTAGCTCAGTCGGTAGAGCATGAGA | 2987 | 2270 | 224 | 421 | 1066,943826 | -1,388862845 | 0,478066299 | -2,905167858 | 0,003670562 | 0,006368699 |
| HUS00124307 | MiRBase | MIMAT0000072 | Homo_sapiens | mature_miRNA | hsa-miR-18a-5p | hsa-miR-18a-5p | TAAGGTGCATCTAGTGCAGATAG | 512 | 617 | 125 | 76 | 252,7384293 | -0,995834103 | 0,346885559 | -2,870785699 | 0,00409453 | 0,007077905 |
| HUS00305139 | MiRBase | MIMAT0003338 | Homo_sapiens | mature_miRNA | hsa-miR-660-5p | hsa-miR-660-5p | TACCCATTGCATATCGGAGTTG | 1036 | 573 | 162 | 87 | 339,735651 | -1,171149306 | 0,410355973 | -2,853983816 | 0,004317473 | 0,007435648 |
| HUS00196430 | MiRBase | MIMAT0003393 | Homo_sapiens | mature_miRNA | hsa-miR-425-5p | hsa-miR-425-5p | AATGACACGATCACTCCCGTTGA | 2725 | 2413 | 2188 | 1253 | 2188,830921 | 0,882431546 | 0,309593884 | 2,850287401 | 0,004367974 | 0,007494863 |
| HUS00157605 | MiRBase | MIMAT0000688 | Homo_sapiens | mature_miRNA | hsa-miR-301a-3p | hsa-miR-301a-3p | CAGTGCAATAGTATTGTCAAAGC | 133 | 160 | 32 | 12 | 61,70008811 | -1,238281748 | 0,440603359 | -2,81042285 | 0,004947645 | 0,00845829 |
| HUS00018123 | GtRNA\|GtRNA | chr11\|chr11 | Homo_sapiens | tRNA | trna16-ValTAC | trna16-ValTAC | GGTTCCATAGTGTAGCGGTTATCACGTCTGCTTTACACGCAGAAGGTCCTGGGTTCGAGCCCCAGTGGAACCA | 480 | 239 | 403 | 209 | 357,4673453 | 1,212591911 | 0,43541653 | 2,784900957 | 0,005354408 | 0,009120145 |
| HUS00054339 | MiRBase | MIMAT0004958 | Homo_sapiens | mature_miRNA | hsa-miR-301b-3p | hsa-miR-301b-3p | CAGTGCAATGATATTGTCAAAGC | 184 | 235 | 43 | 28 | 92,50942906 | -1,054776647 | 0,379617705 | -2,778523325 | 0,005460659 | 0,009267177 |
| HUS00363631 | MiRBase | MIMAT0000758 | Homo_sapiens | mature_miRNA | hsa-miR-135b-5p | hsa-miR-135b-5p | TATGGCTTTTCATTCCTATGTGA | 823 | 928 | 202 | 85 | 378,785862 | -1,130936374 | 0,408812454 | -2,76639413 | 0,005667999 | 0,00958407 |
| HUS00074818 | MiRBase | MIMAT0004956 | Homo_sapiens | mature_miRNA | hsa-miR-374b-3p | hsa-miR-374b-3p | CTTAGCAGGTTGTATTATCATT | 195 | 186 | 41 | 30 | 86,64673884 | -0,909819514 | 0,339143726 | -2,682695992 | 0,007303134 | 0,012304194 |
| HUS00154599 | MiRBase | MIMAT0004673 | Homo_sapiens | mature_miRNA | hsa-miR-29c-5p | hsa-miR-29c-5p | TGACCGATTTCTCCTGGTGTTC | 68 | 67 | 8 | 12 | 28,93177686 | -1,194524456 | 0,448332172 | -2,664373717 | 0,007713182 | 0,012948121 |
| HUS00234961 | MiRBase | MIMAT0004487 | Homo_sapiens | mature_miRNA | hsa-let-7f-2-3p | hsa-let-7f-2-3p | CTATACAGTCTACTGTCTTTCC | 204 | 140 | 19 | 31 | 72,99276584 | -1,187085642 | 0,446319294 | -2,659722891 | 0,007820497 | 0,013081046 |
| HUS00135032 | MiRBase | MIMAT0000680 | Homo_sapiens | mature_miRNA | hsa-miR-106b-5p | hsa-miR-106b-5p | TAAAGTGCTGACAGTGCAGAT | 2245 | 1887 | 472 | 152 | 864,5921731 | -1,245596788 | 0,473659695 | -2,629729322 | 0,008545288 | 0,014242146 |
| HUS00223555 | MiRBase | MIMAT0004688 | Homo_sapiens | mature_miRNA | hsa-miR-374a-3p | hsa-miR-374a-3p | CTTATCAGATTGTATTGTAATT | 784 | 904 | 201 | 133 | 392,2528002 | -0,842146493 | 0,321142968 | -2,622341374 | 0,008732791 | 0,014502671 |
| HUS00240252 | Ensembl | ENST00000386307 | Homo_sapiens | snoRNA | ENSG00000209042 | SNORD12C | GCTGGTGTAAATGATGACTTCACTTTTTTCCCCATCAGATCGACAATGCTGACGTCTTATATTTTGCCAGTTAGTTCTGATACATCGGC | 1709 | 2863 | 478 | 143 | 938,386723 | -1,40154724 | 0,539911381 | -2,595883859 | 0,009434795 | 0,015612739 |
| HUS00137971 | MiRBase | MIMAT0000260 | Homo_sapiens | mature_miRNA | hsa-miR-182-3p | hsa-miR-182-3p | TGGTTCTAGACTTGCCAACTA | 142 | 84 | 21 | 15 | 48,44996086 | -1,106377149 | 0,426714276 | -2,592782129 | 0,009520306 | 0,015698377 |
| HUS00032905 | MiRBase | MIMAT0004692 | Homo_sapiens | mature_miRNA | hsa-miR-340-5p | hsa-miR-340-5p | TTATAAAGCAATGAGACTGATT | 7260 | 6632 | 1317 | 1413 | 3244,523162 | -0,804417529 | 0,312522427 | -2,573951366 | 0,010054441 | 0,016520547 |
| HUS00206089 | Ensembl | ENST00000384390 | Homo_sapiens | snoRNA | ENSG00000207118 | SNORD14D | TCGCTATGATGATGGATTCCAAAACCATTCGTAGTTTCCACCAGAAAGTCTTATGTTGGCCAGTTCCTTCCTTGGATGTTTGAGCGA | 398 | 269 | 278 | 167 | 283,3807336 | 0,886875268 | 0,346845179 | 2,556977358 | 0,010558609 | 0,017287863 |
| HUS00209158 | MiRBase | MIMAT0004504 | Homo_sapiens | mature_miRNA | hsa-miR-31-3p | hsa-miR-31-3p | TGCTATGCCAACATATTGCCAT | 72 | 70 | 17 | 6 | 30,4482879 | -1,112453582 | 0,445091403 | -2,499382318 | 0,012441001 | 0,020298476 |
| HUS00328720 | MiRBase | MIMAT0004551 | Homo_sapiens | mature_miRNA | hsa-miR-30d-3p | hsa-miR-30d-3p | CTTTCAGTCAGATGTTTGCTGC | 457 | 357 | 87 | 76 | 190,0923872 | -0,792751963 | 0,318284868 | -2,490699505 | 0,01274919 | 0,020728578 |
| HUS00172157 | MiRBase | MIMAT0000757 | Homo_sapiens | mature_miRNA | hsa-miR-151a-3p | hsa-miR-151a-3p | CTAGACTGAAGCTCCTTGAGG | 33938 | 37360 | 20960 | 21165 | 28725,38694 | 0,747917658 | 0,301994124 | 2,476596725 | 0,013264168 | 0,021490725 |
| HUS00273335 | GtRNA\|GtRNA\|GtRNA\|GtRNA\|GtRNA\|GtRNA\|GtRNA\|GtRNA\|GtRNA\|GtRNA | chr14\|chr16\|chr16\|chr16\|chr5\|chr14\|chr16\|chr16\|chr16\|chr5 | Homo_sapiens | tRNA | trna6-ProTGG | trna6-ProTGG | GGCTCGTTGGTCTAGGGGTATGATTCTCGCTTTGGGTGCGAGAGGTCCCGGGTTCAAATCCCGGACGAGCCC | 189 | 190 | 18 | 37 | 81,17515605 | -1,177231238 | 0,476115029 | -2,47257735 | 0,013414268 | 0,021658454 |
| HUS00177237 | MiRBase | MIMAT0003886 | Homo_sapiens | mature_miRNA | hsa-miR-769-5p | hsa-miR-769-5p | TGAGACCTCTGGGTTCTGAGCT | 1605 | 1963 | 1038 | 1494 | 1644,369924 | 1,020643073 | 0,417223579 | 2,446273713 | 0,014434137 | 0,023224477 |
| HUS00063789 | MiRBase | MIMAT0004549 | Homo_sapiens | mature_miRNA | hsa-miR-148a-5p | hsa-miR-148a-5p | AAAGTTCTGAGACACTCCGACT | 167 | 312 | 19 | 41 | 99,3793909 | -1,366153511 | 0,56221624 | -2,429943166 | 0,015101191 | 0,024213978 |
| HUS00024184 | MiRBase | MIMAT0000420 | Homo_sapiens | mature_miRNA | hsa-miR-30b-5p | hsa-miR-30b-5p | TGTAAACATCCTACACTCAGCT | 4759 | 4650 | 1227 | 466 | 2088,354328 | -1,004680357 | 0,424041088 | -2,369299543 | 0,017821813 | 0,028478155 |
| HUS00087565 | MiRBase | MIMAT0000750 | Homo_sapiens | mature_miRNA | hsa-miR-340-3p | hsa-miR-340-3p | TCCGTCTCAGTTACTTTATAGC | 109 | 81 | 60 | 59 | 79,10440772 | 0,829655797 | 0,352930636 | 2,350761626 | 0,018735032 | 0,029738678 |
| HUS00167922 | MiRBase | MIMAT0000772 | Homo_sapiens | mature_miRNA | hsa-miR-345-5p | hsa-miR-345-5p | GCTGACTCCTAGTCCAGGGCTC | 558 | 397 | 429 | 214 | 405,3917745 | 0,879816647 | 0,374279935 | 2,350691459 | 0,018738565 | 0,029738678 |
| HUS00237964 | MiRBase | MIMAT0000692 | Homo_sapiens | mature_miRNA | hsa-miR-30e-5p | hsa-miR-30e-5p | TGTAAACATCCTTGACTGGAAG | 5160 | 3231 | 1046 | 426 | 1837,004816 | -1,019455852 | 0,434496886 | -2,346290354 | 0,01896133 | 0,029989858 |
| HUS00123005 | MiRBase | MIMAT0000764 | Homo_sapiens | mature_miRNA | hsa-miR-339-5p | hsa-miR-339-5p | TCCCTGTCCTCCAGGAGCTCACG | 328 | 176 | 221 | 130 | 219,6959013 | 0,945385333 | 0,403511915 | 2,342893227 | 0,019134858 | 0,030161726 |
| HUS00054713 | Ensembl | ENST00000458770 | Homo_sapiens | snoRNA | ENSG00000238531 | SNORD105B | CCACATGCGGCTGATGACAGCACTTCTGCTGAGACGCTGTGATTGCTCTGTCCAAAGTAAACGCCCTGACGCACTGTGG | 2381 | 1404 | 1298 | 1058 | 1554,918187 | 0,818058706 | 0,349700465 | 2,339312608 | 0,019319261 | 0,030349514 |
| HUS00145874 | MiRBase | MIMAT0000753 | Homo_sapiens | mature_miRNA | hsa-miR-342-3p | hsa-miR-342-3p | TCTCACACAGAAATCGCACCCGT | 178 | 514 | 502 | 205 | 392,484212 | 1,358377555 | 0,584738486 | 2,323051393 | 0,020176392 | 0,0315893 |
| HUS00000804 | GtRNA\|GtRNA | chr16\|chr16 | Homo_sapiens | tRNA | trna18-GlyGCC | trna18-GlyGCC | GCATTGGTGGTTCAGTGGTAGAATTCTCGCCTGCCATGCGGGCGGCCGGGCTTCGATTCCTGGCCAATGCA | 20707 | 3779 | 1785 | 967 | 4676,512139 | -1,517541871 | 0,655485365 | -2,315142261 | 0,020605148 | 0,032152328 |
| HUS00192933 | MiRBase | MIMAT0002871 | Homo_sapiens | mature_miRNA | hsa-miR-500a-3p | hsa-miR-500a-3p | ATGCACCTGGGCAAGGATTCTG | 397 | 296 | 256 | 159 | 275,3522978 | 0,736071447 | 0,324333945 | 2,269486311 | 0,023238769 | 0,03614056 |
| HUS00007674 | GtRNA\|GtRNA\|GtRNA\|GtRNA\|GtRNA\|GtRNA\|GtRNA\|GtRNA\|GtRNA\|GtRNA\|GtRNA\|GtRNA | chr1\|chr16\|chr16\|chr17\|chr2\|chr6\|chr1\|chr16\|chr16\|chr17\|chr2\|chr6 | Homo_sapiens | tRNA | trna68-GlyGCC | trna68-GlyGCC | GCATTGGTGGTTCAGTGGTAGAATTCTCGCCTGCCACGCGGGAGGCCCGGGTTCGATTCCCGGCCAATGCA | 23442 | 7963 | 14854 | 10657 | 15287,60081 | 1,166762686 | 0,520607166 | 2,24115756 | 0,025015871 | 0,0387746 |
| HUS00120552 | MiRBase | MIMAT0000087 | Homo_sapiens | mature_miRNA | hsa-miR-30a-5p | hsa-miR-30a-5p | TGTAAACATCCTCGACTGGAAG | 7433 | 7061 | 1539 | 1586 | 3502,71118 | -0,678216465 | 0,303468113 | -2,234885432 | 0,025424871 | 0,039277625 |
| HUS00285455 | MiRBase | MIMAT0000095 | Homo_sapiens | mature_miRNA | hsa-miR-96-5p | hsa-miR-96-5p | TTTGGCACTAGCACATTTTTGCT | 12870 | 9494 | 2954 | 1168 | 4985,925787 | -0,958714361 | 0,430684 | -2,226027345 | 0,02601235 | 0,040052128 |
| HUS00011631 | GtRNA\|GtRNA\|GtRNA\|GtRNA\|GtRNA\|GtRNA | chr6\|chr6\|chr6\|chr6\|chr6\|chr6 | Homo_sapiens | tRNA | trna130-GlnTTG | trna130-GlnTTG | GGCCCCATGGTGTAATGGTTAGCACTCTGGACTTTGAATCCAGCGATCCGAGTTCAAATCTCGGTGGGACCT | 460 | 630 | 134 | 86 | 255,5476968 | -0,816785306 | 0,367596005 | -2,221964587 | 0,026285699 | 0,040339439 |
| HUS00122550 | Ensembl | ENST00000384512 | Homo_sapiens | snoRNA | ENSG00000207241 | SNORD45A | GGTCAATGATGTGTTGGCATGTATTATCTGAATCTATTGCTGATGTGTAATAACACTTTAGCTCTAGAATTACTCTGAGACCTT | 12263 | 4088 | 1360 | 1171 | 3460,166353 | -1,101931343 | 0,503505146 | -2,188520517 | 0,028631708 | 0,043795212 |
| HUS00144499 | GtRNA\|GtRNA | chr16\|chr16 | Homo_sapiens | tRNA | trna25-GlyGCC | trna25-GlyGCC | GCATTGGTGGTTCAGTGGTAGAATTCTCGCCTGCCACGCGGGAGGCCCGGGTTTGATTCCCGGCCAGTGCA | 23421 | 7939 | 14351 | 10550 | 15040,83965 | 1,137379895 | 0,521491152 | 2,18101475 | 0,029182326 | 0,044491086 |
| HUS00015877 | MiRBase | MIMAT0000254 | Homo_sapiens | mature_miRNA | hsa-miR-10b-5p | hsa-miR-10b-5p | TACCCTGTAGAACCGAATTTGTG | 7510 | 12281 | 6167 | 8203 | 9252,679949 | 1,027780097 | 0,476441446 | 2,157201282 | 0,030989985 | 0,047092624 |
| HUS00358483 | MiRBase | MIMAT0004928 | Homo_sapiens | mature_miRNA | hsa-miR-147b | hsa-miR-147b | GTGTGCGGAAATGCTTCTGCTA | 57 | 55 | 5 | 13 | 24,84959074 | -1,054341515 | 0,491095111 | -2,146919185 | 0,031799716 | 0,048165694 |
| HUS00041926 | GtRNA\|GtRNA | chr7\|chr7 | Homo_sapiens | tRNA | trna3-ArgCCT | trna3-ArgCCT | GCCCCAGTGGCCTAATGGATAAGGCATTGGCCTCCTAAGCCAGGGATTGTGGGTTCGAGTCCCATCTGGGGTG | 67 | 76 | 14 | 13 | 32,9234267 | -0,877030801 | 0,41693572 | -2,103515623 | 0,035420721 | 0,053476089 |
| HUS00090560 | MiRBase | MIMAT0004693 | Homo_sapiens | mature_miRNA | hsa-miR-330-5p | hsa-miR-330-5p | TCTCTGGGCCTGTGTCTTAGGC | 375 | 290 | 338 | 129 | 287,5310843 | 0,908970763 | 0,433048342 | 2,099005293 | 0,035816434 | 0,053898517 |
| HUS00166636 | MiRBase | MIMAT0000081 | Homo_sapiens | mature_miRNA | hsa-miR-25-3p | hsa-miR-25-3p | CATTGCACTTGTCTCGGTCTGA | 9663 | 9468 | 7361 | 4129 | 7603,405856 | 0,719788222 | 0,343301536 | 2,096664727 | 0,036023265 | 0,054034898 |
| HUS00059375 | MiRBase | MIMAT0000243 | Homo_sapiens | mature_miRNA | hsa-miR-148a-3p | hsa-miR-148a-3p | TCAGTGCACTACAGAACTTTGT | 31945 | 51984 | 3642 | 8250 | 18008,06423 | -1,175893226 | 0,56924207 | -2,065717362 | 0,038855175 | 0,058095358 |
| HUS00165368 | MiRBase | MIMAT0000101 | Homo_sapiens | mature_miRNA | hsa-miR-103a-3p | hsa-miR-103a-3p | AGCAGCATTGTACAGGGCTATGA | 76511 | 90650 | 72186 | 34209 | 68589,19578 | 0,778374678 | 0,377877884 | 2,059857724 | 0,039412144 | 0,058739253 |
| HUS00216180 | MiRBase | MIMAT0000080 | Homo_sapiens | mature_miRNA | hsa-miR-24-3p | hsa-miR-24-3p | TGGCTCAGTTCAGCAGGAACAG | 81273 | 82807 | 95164 | 29599 | 74428,87498 | 0,986449695 | 0,480178231 | 2,054340724 | 0,039942726 | 0,059339832 |
| HUS00204561 | PiRNA | DQ597347.1 | Homo_sapiens | piRNA | piR-35413 | piR-35413 | GCCCGGCTAGCTCAGTCGGTAGAGCATGAGAC | 9815 | 9113 | 5146 | 5725 | 7509,539914 | 0,71783064 | 0,34972698 | 2,052545788 | 0,04011665 | 0,059408415 |
| HUS00006058 | MiRBase | MIMAT0000416 | Homo_sapiens | mature_miRNA | hsa-miR-1-3p | hsa-miR-1-3p | TGGAATGTAAAGAAGTATGTAT | 1328 | 1311 | 826 | 581 | 983,4690531 | 0,574776666 | 0,280643208 | 2,048069042 | 0,040553235 | 0,059864299 |
| HUS00207346 | MiRBase | MIMAT0010195 | Homo_sapiens | mature_miRNA | hsa-let-7a-2-3p | hsa-let-7a-2-3p | CTGTACAGCCTCCTAGCTTTCC | 64 | 73 | 12 | 14 | 31,76064456 | -0,860780987 | 0,426956839 | -2,016084316 | 0,04379116 | 0,064439523 |
| HUS00224224 | PiRNA | DQ575882.1 | Homo_sapiens | piRNA | piR-43994 | piR-43994 | TCCGTAGTGTAGTGGTTATCACGTTCGCCTCA | 488 | 368 | 157 | 537 | 444,7496066 | 1,233136192 | 0,619995177 | 1,988944814 | 0,046707295 | 0,068513855 |
| HUS00122515 | MiRBase | MIMAT0000759 | Homo_sapiens | mature_miRNA | hsa-miR-148b-3p | hsa-miR-148b-3p | TCAGTGCATCACAGAACTTTGT | 23639 | 27673 | 6865 | 4537 | 12447,40253 | -0,67833317 | 0,342101572 | -1,982841428 | 0,047385145 | 0,069289599 |
| HUS00196798 | MiRBase | MIMAT0005876 | Homo_sapiens | mature_miRNA | hsa-miR-1285-3p | hsa-miR-1285-3p | TCTGGGCAACAAAGTGAGACCT | 87 | 67 | 19 | 12 | 35,76095156 | -0,792708247 | 0,403776878 | -1,963233384 | 0,049619062 | 0,072102699 |
| HUS00338512 | Ensembl\|Ensembl\|Ensembl\|Ensembl\|Ensembl\|Ensembl\|Ensembl | ENST00000580739\|ENST00000581604\|ENST00000582289\|ENST00000579171\|ENST00000582411\|ENST00000583430\|ENST00000584275 | Homo_sapiens | snoRNA | ENSG00000265607 | SNORD84 | GCCATATGATGTTTTCTTTTCGAAAGGTGAGCGCTTTGCGCAGTGATGACCCTCATCTATCACCCTTGACTGATGGCT | 880 | 636 | 218 | 95 | 352,1633137 | -0,791777642 | 0,403142171 | -1,964015922 | 0,049528246 | 0,072102699 |
| HUS00004298 | MiRBase | MIMAT0004701 | Homo_sapiens | mature_miRNA | hsa-miR-338-5p | hsa-miR-338-5p | AACAATATCCTGGTGCTGAGTG | 167 | 87 | 75 | 82 | 105,0187074 | 0,824133987 | 0,42374384 | 1,94488724 | 0,051788551 | 0,075020798 |
| HUS00194110 | MiRBase | MIMAT0000278 | Homo_sapiens | mature_miRNA | hsa-miR-221-3p | hsa-miR-221-3p | AGCTACATTGTCTGCTGGGTTTC | 69280 | 51294 | 63580 | 20997 | 51822,42908 | 0,89167835 | 0,460566231 | 1,936048043 | 0,052861817 | 0,076337717 |
| HUS00009768 | MiRBase | MIMAT0018206 | Homo_sapiens | mature_miRNA | hsa-miR-3929 | hsa-miR-3929 | GAGGCTGATGTGAGTAGACCACT | 65 | 69 | 33 | 49 | 56,02311243 | 0,806790179 | 0,422535945 | 1,909400108 | 0,056210497 | 0,080922232 |
| HUS00001045 | MiRBase | MIMAT0000267 | Homo_sapiens | mature_miRNA | hsa-miR-210-3p | hsa-miR-210-3p | CTGTGCGTGTGACAGCGGCTGA | 1046 | 986 | 1075 | 352 | 875,4056875 | 0,884554327 | 0,470681146 | 1,879306903 | 0,0602026 | 0,086401879 |
| HUS00066662 | Ensembl | ENST00000581525 | Homo_sapiens | snoRNA | ENSG00000264294 | SNORD55 | CGTGTATGATGACAACTCGGTAATGCTGCATACTCCCGAGTGCGCGGTGGGGAAGCCAACCTTGGAGAGCTGAGCGTGCG | 7346 | 7948 | 1771 | 1777 | 3807,727661 | -0,580196087 | 0,310772729 | -1,866946593 | 0,061909046 | 0,088577559 |
| HUS00212169 | PiRNA | DQ570992.1 | Homo_sapiens | piRNA | piR-31104 | piR-31104 | AGCCCGGCTAGCTCAGTCGGTAGAGCATGAGA | 4835 | 5256 | 2579 | 2865 | 3857,03847 | 0,625153388 | 0,335737497 | 1,862030285 | 0,062598821 | 0,089016673 |
| HUS00301914 | PiRNA | DQ597346.1 | Homo_sapiens | piRNA | piR-35412 | piR-35412 | GCCCGGCTAGCTCAGTCGGTAGAGCATGAGA | 4835 | 5256 | 2579 | 2865 | 3857,03847 | 0,625153388 | 0,335737497 | 1,862030285 | 0,062598821 | 0,089016673 |
| HUS00120713 | GtRNA\|GtRNA | chr11\|chr11 | Homo_sapiens | tRNA | trna5-LysTTT | trna5-LysTTT | GCCCGGATAGCTCAGTCGGTAGAGCATCAGACTTTTAATCTGAGGGTCCGGGGTTCAAGTCCCTGTTCGGGCG | 317 | 572 | 90 | 87 | 210,1117202 | -0,803702949 | 0,44126458 | -1,821362934 | 0,068551706 | 0,097184583 |
| HUS00051340 | MiRBase | MIMAT0000064 | Homo_sapiens | mature_miRNA | hsa-let-7c-5p | hsa-let-7c-5p | TGAGGTAGTAGGTTGTATGGTT | 17108 | 11151 | 7285 | 9500 | 11488,82321 | 0,781608098 | 0,430648893 | 1,814954389 | 0,069530919 | 0,098273184 |
| HUS00163831 | PiRNA | DQ575881.1 | Homo_sapiens | piRNA | piR-43993 | piR-43993 | TCCGTAGTGTAGTGGTTATCACGTTCGCCT | 450 | 360 | 133 | 475 | 399,1966373 | 1,127954124 | 0,626603283 | 1,800108865 | 0,07184345 | 0,101233952 |
| HUS00002012 | MiRBase | MIMAT0002870 | Homo_sapiens | mature_miRNA | hsa-miR-499a-5p | hsa-miR-499a-5p | TTAAGACTTGCAGTGATGTTT | 885 | 474 | 163 | 127 | 322,4249938 | -0,692958108 | 0,388185698 | -1,785120142 | 0,074241831 | 0,104297437 |
| HUS00151922 | MiRBase | MIMAT0003150 | Homo_sapiens | mature_miRNA | hsa-miR-455-5p | hsa-miR-455-5p | TATGTGCCTTTGGACTACATCG | 705 | 478 | 150 | 125 | 291,5551454 | -0,578659632 | 0,330162164 | -1,752652775 | 0,079661627 | 0,111574266 |
| HUS00066746 | Ensembl | ENST00000459475 | Homo_sapiens | snoRNA | ENSG00000238344 | SNORD126 | AGTTTGCCATGATGAAATGCATGTTAAGTCCGTGTTTCAGCTGATCAGCCTGATTAAACACATGCTCTGAGCAGACT | 720 | 1052 | 247 | 153 | 433,0993944 | -0,663742305 | 0,381851165 | -1,73822255 | 0,082171607 | 0,114744136 |
| HUS00159377 | MiRBase | MIMAT0001545 | Homo_sapiens | mature_miRNA | hsa-miR-450a-5p | hsa-miR-450a-5p | TTTTGCGATGTGTTCCTAATAT | 3644 | 3394 | 910 | 839 | 1794,534464 | -0,48695819 | 0,28038705 | -1,736735664 | 0,08243384 | 0,114765675 |
| HUS00316666 | Ensembl | ENST00000391002 | Homo_sapiens | snoRNA | ENSG00000212304 | SNORD12 | GCCTTTGCAGCTGATGATACAGCTTCTTTCCCCATCAGATCGACCCTGTTGATCTCTACACTATTGGCCAGTTTTGTCTGATGCATTGGC | 10885 | 9759 | 3255 | 1627 | 5078,39752 | -0,60428885 | 0,356070067 | -1,697106569 | 0,089676515 | 0,124476356 |
| HUS00042578 | MiRBase | MIMAT0002883 | Homo_sapiens | mature_miRNA | hsa-miR-514a-3p | hsa-miR-514a-3p | ATTGACACTTCTGTGAGTAGA | 63 | 113 | 22 | 14 | 41,60576494 | -0,786998808 | 0,47431741 | -1,659223951 | 0,097070673 | 0,134338878 |
| HUS00110371 | MiRBase | MIMAT0004909 | Homo_sapiens | mature_miRNA | hsa-miR-450b-5p | hsa-miR-450b-5p | TTTTGCAATATGTTCCTGAATA | 1404 | 2011 | 351 | 400 | 838,3144732 | -0,647309573 | 0,398740632 | -1,623385031 | 0,104507123 | 0,144201222 |
| HUS00158576 | GtRNA\|GtRNA\|GtRNA\|GtRNA | chr13\|chr2\|chr13\|chr2 | Homo_sapiens | tRNA | trna5-GluTTC | trna5-GluTTC | TCCCATATGGTCTAGCGGTTAGGATTCCTGGTTTTCACCCAGGTGGCCCGGGTTCGACTCCCGGTATGGGAA | 4397 | 3207 | 2169 | 1569 | 2696,338931 | 0,470534318 | 0,290985181 | 1,617038769 | 0,105869913 | 0,1455321 |
| HUS00094161 | MiRBase | MIMAT0005878 | Homo_sapiens | mature_miRNA | hsa-miR-1287-5p | hsa-miR-1287-5p | TGCTGGATCAGTGGTTCGAGTC | 446 | 336 | 246 | 155 | 282,9041919 | 0,517328708 | 0,320132159 | 1,615984818 | 0,106097596 | 0,1455321 |
| HUS00086448 | Ensembl | ENST00000384468 | Homo_sapiens | snoRNA | ENSG00000207197 | SNORD116-12 | GGATCAATGATGACTTCCATATATACATTCCTTGGAAAGCTGAATAAAATGAATGAAAACTCTATACCATCATCCTCATTGAACTGAGGTCC | 88 | 133 | 33 | 15 | 52,89329089 | -0,721050391 | 0,452812889 | -1,592380448 | 0,111299247 | 0,152218088 |
| HUS00018689 | Ensembl | ENST00000362803 | Homo_sapiens | snoRNA | ENSG00000199673 | SNORD16 | TGCAATGATGTCGTAATTTGCGTCTTACTCTGTTCTCAGCGACAGTTGCCTGCTGTCAGTAAGCTGGTACAGAAGGTTGACGAAAATTCTTACTGAGCA | 296 | 734 | 85 | 108 | 240,6501027 | -0,858477794 | 0,539957978 | -1,589897417 | 0,11185793 | 0,152533541 |
| HUS00060833 | MiRBase | MIMAT0000682 | Homo_sapiens | mature_miRNA | hsa-miR-200a-3p | hsa-miR-200a-3p | TAACACTGTCTGGTAACGATGT | 131622 | 82892 | 23260 | 25828 | 52865,62842 | -0,570041899 | 0,359218984 | -1,586892466 | 0,112537001 | 0,153010835 |
| HUS00185767 | Ensembl | ENST00000383875 | Homo_sapiens | snoRNA | ENSG00000206602 | SNORD58A | CTGCAGTGATGACTTTCTTAGGACACCTTTGGATTTACCGTGAAAATTAATAAATTCTGAGCAGC | 1356 | 2462 | 951 | 1320 | 1570,243016 | 0,751246583 | 0,477836686 | 1,572182725 | 0,115908181 | 0,157134997 |
| HUS00334209 | MiRBase | MIMAT0000705 | Homo_sapiens | mature_miRNA | hsa-miR-362-5p | hsa-miR-362-5p | AATCCTTGGAACCTAGGTGTGAGT | 394 | 398 | 268 | 144 | 288,1028817 | 0,514855188 | 0,341029405 | 1,509709076 | 0,131117674 | 0,176723822 |
| HUS00244129 | MiRBase | MIMAT0000094 | Homo_sapiens | mature_miRNA | hsa-miR-95-3p | hsa-miR-95-3p | TTCAACGGGTATTTATTGAGCA | 2279 | 2824 | 1210 | 1412 | 1899,693122 | 0,554029265 | 0,36693873 | 1,509868596 | 0,131076957 | 0,176723822 |
| HUS00130773 | PiRNA | DQ570991.1 | Homo_sapiens | piRNA | piR-31103 | piR-31103 | AGCCCGGCTAGCTCAGTCGGTAGAGCATGAG | 2816 | 3207 | 1323 | 1822 | 2276,045098 | 0,591406548 | 0,395315609 | 1,49603642 | 0,134644165 | 0,180430941 |
| HUS00283633 | PiRNA | DQ597345.1 | Homo_sapiens | piRNA | piR-35411 | piR-35411 | GCCCGGCTAGCTCAGTCGGTAGAGCATGAG | 2816 | 3207 | 1323 | 1822 | 2276,045098 | 0,591406548 | 0,395315609 | 1,49603642 | 0,134644165 | 0,180430941 |
| HUS00022334 | MiRBase | MIMAT0002874 | Homo_sapiens | mature_miRNA | hsa-miR-503-5p | hsa-miR-503-5p | TAGCAGCGGGAACAGTTCTGCAG | 541 | 782 | 328 | 375 | 502,8450032 | 0,591589418 | 0,400750376 | 1,476204276 | 0,139889062 | 0,18692073 |
| HUS00074709 | MiRBase | MIMAT0000417 | Homo_sapiens | mature_miRNA | hsa-miR-15b-5p | hsa-miR-15b-5p | TAGCAGCACATCATGGTTTACA | 359 | 427 | 387 | 110 | 315,8214075 | 0,718344057 | 0,512747937 | 1,400969181 | 0,161223289 | 0,2148104 |
| HUS00230717 | Ensembl | ENST00000383893 | Homo_sapiens | snoRNA | ENSG00000206620 | SNORD45C | GGTCAATGATGAGTTGGCATGTATTCTGAATCTAAAGTTGATTATTACTACTTTAGCTCTAGAATTACTCTGAGACCTG | 20909 | 1433 | 1010 | 819 | 3977,55081 | -1,518011049 | 1,084754893 | -1,399404657 | 0,161691671 | 0,214818935 |
| HUS00079848 | GtRNA\|GtRNA | chr19\|chr19 | Homo_sapiens | tRNA | trna13-ValCAC | trna13-ValCAC | GTTTCCGTAGTGTAGCGGTTATCACATTCGCCTCACACGCGAAAGGTCCCCGGTTCGATCCCGGGCGGAAACA | 277 | 861 | 327 | 402 | 490,1830434 | 0,812892004 | 0,582408973 | 1,395740866 | 0,162792543 | 0,215665335 |
| HUS00106205 | MiRBase | MIMAT0004702 | Homo_sapiens | mature_miRNA | hsa-miR-339-3p | hsa-miR-339-3p | TGAGCGCCTCGACGACAGAGCCG | 630 | 342 | 267 | 225 | 351,6918502 | 0,528381366 | 0,381363565 | 1,38550563 | 0,165897912 | 0,218617929 |
| HUS00340664 | MiRBase | MIMAT0000252 | Homo_sapiens | mature_miRNA | hsa-miR-7-5p | hsa-miR-7-5p | TGGAAGACTAGTGATTTTGTTGT | 814 | 1019 | 406 | 536 | 685,5990465 | 0,561050821 | 0,405003897 | 1,385297338 | 0,165961567 | 0,218617929 |
| HUS00299348 | MiRBase | MIMAT0000455 | Homo_sapiens | mature_miRNA | hsa-miR-185-5p | hsa-miR-185-5p | TGGAGAGAAAGGCAGTTCCTGA | 4475 | 2914 | 1083 | 818 | 1892,777435 | -0,44182984 | 0,320815601 | -1,377208085 | 0,168447923 | 0,221266339 |
| HUS00108901 | MiRBase | MIMAT0000456 | Homo_sapiens | mature_miRNA | hsa-miR-186-5p | hsa-miR-186-5p | CAAAGAATTCTCCTTTTGGGCT | 13555 | 6924 | 8966 | 3381 | 8001,804588 | 0,695758148 | 0,508514916 | 1,368215809 | 0,171244534 | 0,224306221 |
| HUS00264750 | PiRNA | DQ594465.1 | Homo_sapiens | piRNA | piR-60577 | piR-60577 | TTCCGTAGTGTAGTGGTTATCACGTTCGCCTC | 964 | 554 | 246 | 640 | 624,6364536 | 0,790250141 | 0,579379147 | 1,363960276 | 0,172580068 | 0,225420595 |
| HUS00251475 | Ensembl | ENST00000384252 | Homo_sapiens | snoRNA | ENSG00000206979 | SNORD61 | GCTATGATGAATTTGATTGCATTGATCGTCTGACATGATAATGTATTTTTGTCCTCTAAGAAGTTCTGAGCTT | 603 | 749 | 115 | 815 | 638,9161564 | 0,988552093 | 0,757455091 | 1,305096638 | 0,191859943 | 0,249901607 |
| HUS00115905 | MiRBase | MIMAT0000438 | Homo_sapiens | mature_miRNA | hsa-miR-152-3p | hsa-miR-152-3p | TCAGTGCATGACAGAACTTGG | 1437 | 1402 | 1223 | 407 | 1078,020342 | 0,605317102 | 0,465351028 | 1,300775255 | 0,193335394 | 0,251119995 |
| HUS00329822 | MiRBase | MIMAT0000441 | Homo_sapiens | mature_miRNA | hsa-miR-9-5p | hsa-miR-9-5p | TCTTTGGTTATCTAGCTGTATGA | 4240 | 5190 | 3099 | 1642 | 3366,970224 | 0,455768874 | 0,357629178 | 1,274417475 | 0,202515502 | 0,262311166 |
| HUS00244573 | GtRNA\|GtRNA\|GtRNA\|GtRNA\|GtRNA\|GtRNA\|GtRNA\|GtRNA\|GtRNA\|GtRNA | chr1\|chr1\|chr11\|chr17\|chr6\|chr1\|chr1\|chr11\|chr17\|chr6 | Homo_sapiens | tRNA | trna54-LysTTT | trna54-LysTTT | GCCCGGATAGCTCAGTCGGTAGAGCATCAGACTTTTAATCTGAGGGTCCAGGGTTCAAGTCCCTGTTCGGGCG | 883 | 1327 | 232 | 290 | 559,6907745 | -0,538789402 | 0,426036166 | -1,26465649 | 0,205994494 | 0,266076221 |
| HUS00270258 | Ensembl\|Ensembl | ENST00000384462\|ENST00000384404 | Homo_sapiens | snoRNA | ENSG00000207191 | SNORD116-5 | GGATCGATGATGAGTCCCCCATAAAAACATTCCTTGGAAAAGCTGAACAAAATGAGTGAGAACTCATACCGTCGTTCTCATCAGAACTGAGGTCC | 1217 | 526 | 233 | 174 | 426,753288 | -0,559023882 | 0,444322226 | -1,258149715 | 0,208337617 | 0,268357318 |
| HUS00176710 | MiRBase | MIMAT0000104 | Homo_sapiens | mature_miRNA | hsa-miR-107 | hsa-miR-107 | AGCAGCATTGTACAGGGCTATCA | 4597 | 3486 | 3709 | 1048 | 3094,043786 | 0,631421087 | 0,505855526 | 1,248224157 | 0,21194898 | 0,272254905 |
| HUS00021488 | MiRBase | MIMAT0000083 | Homo_sapiens | mature_miRNA | hsa-miR-26b-5p | hsa-miR-26b-5p | TTCAAGTAATTCAGGATAGGT | 27904 | 17312 | 16079 | 7600 | 16367,2735 | 0,520667359 | 0,42062722 | 1,237835628 | 0,215777015 | 0,276408573 |
| HUS00101701 | MiRBase | MIMAT0002888 | Homo_sapiens | mature_miRNA | hsa-miR-532-5p | hsa-miR-532-5p | CATGCCTTGAGTGTAGGACCGT | 19866 | 22774 | 8464 | 13133 | 15889,18063 | 0,554114708 | 0,449109014 | 1,233808919 | 0,217274115 | 0,277561713 |
| HUS00154466 | Ensembl | ENST00000628177 | Homo_sapiens | snoRNA | ENSG00000281010 | snoR1 | GTCTGTGATGAATTGCTTTGACTTCTGACACCTCGTATGAAAACTGCACGTGCAGTCTGATTATTTAGCAAGACTGAGGC | 1074 | 1155 | 609 | 415 | 761,5817886 | 0,356810188 | 0,298851129 | 1,193939565 | 0,232501609 | 0,29620068 |
| HUS00221643 | MiRBase | MIMAT0000264 | Homo_sapiens | mature_miRNA | hsa-miR-203a-3p | hsa-miR-203a-3p | GTGAAATGTTTAGGACCACTAG | 9123 | 16774 | 3007 | 3180 | 6577,815253 | -0,539123924 | 0,457255326 | -1,179043508 | 0,238380852 | 0,302860918 |
| HUS00299630 | MiRBase | MIMAT0004559 | Homo_sapiens | mature_miRNA | hsa-miR-181c-3p | hsa-miR-181c-3p | AACCATCGACCGTTGAGTGGAC | 355 | 371 | 152 | 187 | 255,6111482 | 0,42622937 | 0,366598249 | 1,162660683 | 0,2449672 | 0,310380784 |
| HUS00145689 | Ensembl | ENST00000364849 | Homo_sapiens | snoRNA | ENSG00000254341 | SNORD87 | GCTGGCACAATGATGACTTAAATTACTTTTTGCCGTTTACCCAGCTGAGGTTGTCTTTGAAGAAATAATTTTAAGACTGAGATGCCAGT | 844 | 1393 | 961 | 329 | 856,3226206 | 0,592472447 | 0,511325956 | 1,158698167 | 0,246579238 | 0,311574309 |
| HUS00086527 | Ensembl\|Ensembl | ENST00000384287\|ENST00000384000 | Homo_sapiens | snoRNA | ENSG00000207014 | SNORD116-3 | GGATCGATGATGAGTCCCCCATAAAAACATTCCTTGGAAAAGCTGAACAAAATGAGTGAGAACTCATACCGTCGTTCTCATCGGAACTGAGGTCC | 1286 | 575 | 253 | 200 | 464,2650911 | -0,497950905 | 0,434761898 | -1,145341641 | 0,252067663 | 0,317646242 |
| HUS00307885 | MiRBase | MIMAT0002876 | Homo_sapiens | mature_miRNA | hsa-miR-505-3p | hsa-miR-505-3p | CGTCAACACTTGCTGGTTTCCT | 77 | 101 | 34 | 10 | 44,29543992 | -0,555090556 | 0,486603142 | -1,140745934 | 0,253975666 | 0,319185634 |
| HUS00068672 | MiRBase | MIMAT0000445 | Homo_sapiens | mature_miRNA | hsa-miR-126-3p | hsa-miR-126-3p | TCGTACCGTGAGTAATAATGCG | 3319 | 2831 | 1123 | 553 | 1602,231781 | -0,403205333 | 0,354156924 | -1,138493437 | 0,254914498 | 0,319501999 |
| HUS00101578 | GtRNA\|GtRNA\|GtRNA\|GtRNA\|GtRNA\|GtRNA\|GtRNA\|GtRNA\|GtRNA\|GtRNA | chr15\|chr17\|chr6\|chr6\|chr6\|chr15\|chr17\|chr6\|chr6\|chr6 | Homo_sapiens | tRNA | trna7-GlnCTG | trna7-GlnCTG | GGTTCCATGGTGTAATGGTTAGCACTCTGGACTCTGAATCCAGCGATCCGAGTTCAAATCTCGGTGGAACCT | 4024 | 12191 | 4311 | 5001 | 6502,387416 | 0,660273265 | 0,583473739 | 1,131624649 | 0,257792273 | 0,322240341 |
| HUS00014238 | Ensembl\|Ensembl | ENST00000458961\|ENST00000459128 | Homo_sapiens | snoRNA | ENSG00000239169 | SNORD109B | GGATCGATGATGAGAATAATTGTCTGAGGATGCTGAGGGACTCATTCCAGATGTCAATCTGAGGTCC | 418 | 224 | 97 | 67 | 163,1854971 | -0,448661082 | 0,399488512 | -1,123088822 | 0,261399773 | 0,325637487 |
| HUS00188312 | MiRBase | MIMAT0004796 | Homo_sapiens | mature_miRNA | hsa-miR-576-3p | hsa-miR-576-3p | AAGATGTGGAAAAATTGGAATC | 195 | 151 | 45 | 46 | 90,39910526 | -0,395703281 | 0,352712312 | -1,121886783 | 0,261910581 | 0,325637487 |
| HUS00149815 | MiRBase | MIMAT0018186 | Homo_sapiens | mature_miRNA | hsa-miR-3912-3p | hsa-miR-3912-3p | TAACGCATAATATGGACATGT | 112 | 150 | 47 | 21 | 67,10500853 | -0,475300743 | 0,427620273 | -1,111501892 | 0,266352376 | 0,330276946 |
| HUS00055793 | MiRBase | MIMAT0000089 | Homo_sapiens | mature_miRNA | hsa-miR-31-5p | hsa-miR-31-5p | AGGCAAGATGCTGGCATAGCT | 2995 | 2400 | 2105 | 735 | 1944,414512 | 0,495103649 | 0,450184032 | 1,099780565 | 0,271427742 | 0,334146328 |
| HUS00261293 | MiRBase | MIMAT0002819 | Homo_sapiens | mature_miRNA | hsa-miR-193b-3p | hsa-miR-193b-3p | AACTGGCCCTCAAAGTCCCGCT | 65 | 84 | 27 | 47 | 55,15167636 | 0,51700771 | 0,469649178 | 1,100838101 | 0,270967126 | 0,334146328 |
| HUS00149512 | MiRBase | MIMAT0004481 | Homo_sapiens | mature_miRNA | hsa-let-7a-3p | hsa-let-7a-3p | CTATACAATCTACTGTCTTTC | 388 | 77 | 140 | 125 | 180,0471047 | 0,691033004 | 0,628600563 | 1,099319734 | 0,271628628 | 0,334146328 |
| HUS00279417 | PiRNA | DQ594464.1 | Homo_sapiens | piRNA | piR-60576 | piR-60576 | TTCCGTAGTGTAGTGGTTATCACGTTCGCC | 761 | 477 | 182 | 450 | 468,0880613 | 0,604534703 | 0,559099817 | 1,081264355 | 0,279579539 | 0,343019751 |
| HUS00203520 | MiRBase | MIMAT0000707 | Homo_sapiens | mature_miRNA | hsa-miR-363-3p | hsa-miR-363-3p | AATTGCACGGTATCCATCTGTA | 108 | 95 | 32 | 23 | 53,29790659 | -0,377677159 | 0,357199425 | -1,057328575 | 0,290361656 | 0,354378399 |
| HUS00282547 | GtRNA\|GtRNA | chr6\|chr6 | Homo_sapiens | tRNA | trna42-GlnCTG | trna42-GlnCTG | GGTTCCATGGTGTAATGGTTAGCACTCTGGACTCTGAATCCGGTAATCCGAGTTCAAATCTCGGTGGAACCT | 862 | 2882 | 697 | 1452 | 1533,8608 | 0,691159992 | 0,653279537 | 1,057985063 | 0,290062252 | 0,354378399 |
| HUS00114474 | MiRBase | MIMAT0000070 | Homo_sapiens | mature_miRNA | hsa-miR-17-5p | hsa-miR-17-5p | CAAAGTGCTTACAGTGCAGGTAG | 7079 | 6393 | 6091 | 1524 | 5023,313464 | 0,55877367 | 0,541252569 | 1,032371396 | 0,301898167 | 0,367493842 |
| HUS00325638 | GtRNA\|GtRNA | chr6\|chr6 | Homo_sapiens | tRNA | trna146-GlnCTG | trna146-GlnCTG | GGTTCCATGGTGTAATGGTTAGCACTCTGGACTCTGAATCCAGCGATCCGAGTTCAAGTCTCGGTGGAACCT | 3990 | 11482 | 3815 | 4579 | 5997,610865 | 0,588547715 | 0,572158179 | 1,028645112 | 0,303646471 | 0,368656943 |
| HUS00011339 | MiRBase | MIMAT0000765 | Homo_sapiens | mature_miRNA | hsa-miR-335-5p | hsa-miR-335-5p | TCAAGAGCAATAACGAAAAATGT | 1247 | 719 | 936 | 207 | 740,4055673 | 0,598277034 | 0,58901557 | 1,015723631 | 0,309761012 | 0,375101225 |
| HUS00316822 | MiRBase | MIMAT0018925 | Homo_sapiens | mature_miRNA | hsa-miR-1268b | hsa-miR-1268b | CGGGCGTGGTGGTGGGGGTG | 67 | 71 | 31 | 32 | 47,67350573 | 0,372442643 | 0,371367112 | 1,002896138 | 0,315910976 | 0,381131972 |
| HUS00203479 | MiRBase | MIMAT0000732 | Homo_sapiens | mature_miRNA | hsa-miR-378a-3p | hsa-miR-378a-3p | ACTGGACTTGGAGTCAGAAGGC | 22890 | 24322 | 9230 | 12314 | 16453,83134 | 0,401539187 | 0,400768291 | 1,001923547 | 0,316380519 | 0,381131972 |
| HUS00221936 | MiRBase | MIMAT0005922 | Homo_sapiens | mature_miRNA | hsa-miR-1268a | hsa-miR-1268a | CGGGCGTGGTGGTGGGGG | 66 | 70 | 31 | 31 | 46,89269982 | 0,368670661 | 0,369756133 | 0,997064357 | 0,318733273 | 0,382974087 |
| HUS00127823 | MiRBase | MIMAT0000069 | Homo_sapiens | mature_miRNA | hsa-miR-16-5p | hsa-miR-16-5p | TAGCAGCACGTAAATATTGGCG | 3137 | 2932 | 1213 | 414 | 1558,73411 | -0,450975446 | 0,454439599 | -0,992377088 | 0,321013612 | 0,384719921 |
| HUS00363289 | MiRBase | MIMAT0000063 | Homo_sapiens | mature_miRNA | hsa-let-7b-5p | hsa-let-7b-5p | TGAGGTAGTAGGTTGTGTGGTT | 57716 | 73599 | 27438 | 30009 | 44461,16099 | 0,321790157 | 0,34523558 | 0,932088624 | 0,351290726 | 0,419923362 |
| HUS00208746 | Ensembl | ENST00000363981 | Homo_sapiens | snoRNA | ENSG00000275996 | SNORD27 | ACTCCATGATGAACACAAAATGACAAGCATATGGCTGAACTTTCAAGTGATGTCATCTTACTACTGAGAAGT | 11421 | 6068 | 5136 | 3020 | 5955,210632 | 0,381573069 | 0,410269828 | 0,930053938 | 0,352343158 | 0,420101458 |
| HUS00196288 | MiRBase | MIMAT0000066 | Homo_sapiens | mature_miRNA | hsa-let-7e-5p | hsa-let-7e-5p | TGAGGTAGGAGGTTGTATAGTT | 8062 | 7467 | 3956 | 2690 | 5093,509836 | 0,260604034 | 0,28149426 | 0,925788092 | 0,354556117 | 0,421658809 |
| HUS00343657 | MiRBase | MIMAT0004589 | Homo_sapiens | mature_miRNA | hsa-miR-30b-3p | hsa-miR-30b-3p | CTGGGAGGTGGATGTTTACTTC | 61 | 66 | 23 | 12 | 33,39692199 | -0,367828066 | 0,405581518 | -0,906915254 | 0,364451614 | 0,431221375 |
| HUS00338938 | MiRBase | MIMAT0000414 | Homo_sapiens | mature_miRNA | hsa-let-7g-5p | hsa-let-7g-5p | TGAGGTAGTAGTTTGTACAGTT | 342591 | 330935 | 108307 | 84406 | 181750,8747 | -0,30044869 | 0,331141228 | -0,907312846 | 0,364241383 | 0,431221375 |
| HUS00190386 | MiRBase | MIMAT0000457 | Homo_sapiens | mature_miRNA | hsa-miR-188-5p | hsa-miR-188-5p | CATCCCTTGCATGGTGGAGGG | 65 | 53 | 27 | 4 | 29,57933685 | -0,4727092 | 0,554922756 | -0,851846847 | 0,394299101 | 0,464369712 |
| HUS00114989 | MiRBase | MIMAT0000077 | Homo_sapiens | mature_miRNA | hsa-miR-22-3p | hsa-miR-22-3p | AAGCTGCCAGTTGAAGAACTGT | 68170 | 54557 | 36993 | 17891 | 40716,0032 | 0,296505786 | 0,348195903 | 0,851548752 | 0,394464594 | 0,464369712 |
| HUS00291447 | GtRNA\|GtRNA\|GtRNA\|GtRNA | chr13\|chr15\|chr13\|chr15 | Homo_sapiens | tRNA | trna3-GluTTC | trna3-GluTTC | TCCCACATGGTCTAGCGGTTAGGATTCCTGGTTTTCACCCAGGCGGCCCGGGTTCGACTCCCGGTGTGGGAA | 4142 | 41528 | 21132 | 16602 | 22992,32662 | 0,892507052 | 1,055263546 | 0,84576697 | 0,397682755 | 0,466975962 |
| HUS00107267 | PiRNA | DQ597344.1 | Homo_sapiens | piRNA | piR-35410 | piR-35410 | GCCCGGCTAGCTCAGTCGGTAGAGCATGA | 1974 | 2563 | 819 | 1185 | 1559,555295 | 0,3545411 | 0,426644592 | 0,830998697 | 0,405974366 | 0,475511537 |
| HUS00209723 | MiRBase | MIMAT0005919 | Homo_sapiens | mature_miRNA | hsa-miR-548o-3p | hsa-miR-548o-3p | CCAAAACTGCAGTTACTTTTGC | 110 | 140 | 58 | 51 | 83,84647125 | 0,295588578 | 0,3627313 | 0,814896807 | 0,415131381 | 0,485015307 |
| HUS00321713 | MiRBase | MIMAT0000727 | Homo_sapiens | mature_miRNA | hsa-miR-374a-5p | hsa-miR-374a-5p | TTATAATACAACCTGATAAGTG | 1343 | 1112 | 545 | 116 | 625,0633025 | -0,459063568 | 0,575572321 | -0,79757756 | 0,425115677 | 0,495435564 |
| HUS00231444 | MiRBase | MIMAT0004697 | Homo_sapiens | mature_miRNA | hsa-miR-151a-5p | hsa-miR-151a-5p | TCGAGGAGCTCACAGTCTAGT | 66367 | 10032 | 53567 | 7413 | 34667,34639 | 0,838980151 | 1,05480643 | 0,795387786 | 0,426387963 | 0,495676007 |
| HUS00322632 | GtRNA\|GtRNA\|GtRNA\|GtRNA\|GtRNA\|GtRNA\|GtRNA\|GtRNA\|GtRNA\|GtRNA\|GtRNA\|GtRNA\|GtRNA\|GtRNA | chr1\|chr1\|chr1\|chr1\|chr1\|chr6\|chr6\|chr1\|chr1\|chr1\|chr1\|chr1\|chr6\|chr6 | Homo_sapiens | tRNA | trna116-GluCTC | trna116-GluCTC | TCCCTGGTGGTCTAGTGGTTAGGATTCGGCGCTCTCACCGCCGCGGCCCGGGTTCGATTCCCGGTCAGGGAA | 3764 | 72488 | 24892 | 40944 | 40915,95746 | 0,88649447 | 1,151093041 | 0,770132768 | 0,44122114 | 0,511640474 |
| HUS00224636 | MiRBase | MIMAT0030417 | Homo_sapiens | mature_miRNA | hsa-miR-6516-5p | hsa-miR-6516-5p | TTTGCAGTAACAGGTGTGAGCA | 92 | 65 | 33 | 34 | 52,0147436 | 0,288036742 | 0,378531485 | 0,760932058 | 0,44669765 | 0,516702505 |
| HUS00045284 | MiRBase | MIMAT0000460 | Homo_sapiens | mature_miRNA | hsa-miR-194-5p | hsa-miR-194-5p | TGTAACAGCAACTCCATGTGGA | 851 | 583 | 513 | 171 | 486,915391 | 0,358865899 | 0,47776017 | 0,751142356 | 0,452566988 | 0,522192678 |
| HUS00092501 | MiRBase | MIMAT0010214 | Homo_sapiens | mature_miRNA | hsa-miR-151b | hsa-miR-151b | TCGAGGAGCTCACAGTCT | 2296 | 251 | 2033 | 212 | 1230,269673 | 0,853495479 | 1,145932112 | 0,744804574 | 0,456389869 | 0,525300221 |
| HUS00132200 | Ensembl | ENST00000362883 | Homo_sapiens | snoRNA | ENSG00000199753 | SNORD104 | GCTGTGATGACATTCCAATTAAAGCACGTGTTAGACTGCTGACGCGGGTGATGCGAACTGGAGTCTGAGC | 1496 | 1077 | 209 | 1002 | 942,7845944 | 0,510544294 | 0,688507255 | 0,741523478 | 0,458376102 | 0,526283672 |
| HUS00234511 | MiRBase | MIMAT0018926 | Homo_sapiens | mature_miRNA | hsa-miR-378d | hsa-miR-378d | ACTGGACTTGGAGTCAGAAA | 1664 | 1193 | 360 | 442 | 771,3614718 | -0,277573857 | 0,377012164 | -0,736246422 | 0,461580746 | 0,527852952 |
| HUS00273560 | MiRBase | MIMAT0016847 | Homo_sapiens | mature_miRNA | hsa-miR-378c | hsa-miR-378c | ACTGGACTTGGAGTCAGAAGAGTGG | 5675 | 4689 | 1636 | 1494 | 2873,945568 | -0,206137859 | 0,280255361 | -0,735535826 | 0,462013229 | 0,527852952 |
| HUS00363967 | MiRBase | MIMAT0000082 | Homo_sapiens | mature_miRNA | hsa-miR-26a-5p | hsa-miR-26a-5p | TTCAAGTAATCCAGGATAGGCT | 229978 | 128769 | 93641 | 61457 | 117591,0828 | 0,283994143 | 0,390765699 | 0,726763235 | 0,467371009 | 0,532665488 |
| HUS00020859 | GtRNA\|GtRNA | chr1\|chr1 | Homo_sapiens | tRNA | trna59-GluCTC | trna59-GluCTC | TCCCTGGTGGTCTAGTGGTTAGGATTCGGCGCTCTCACCGCCGCGGCCCGGGTTCGATTCCCGGTCAGGAAA | 3657 | 72423 | 21734 | 39918 | 39212,81262 | 0,822570626 | 1,157098118 | 0,710890989 | 0,47715179 | 0,542483087 |
| HUS00352572 | PiRNA | DQ600952.1 | Homo_sapiens | piRNA | piR-39018 | piR-39018 | TAGAGCATGAGACTCTTAATCTCAGGGTCGTG | 297 | 143 | 56 | 139 | 152,6575695 | 0,413506507 | 0,584028054 | 0,708025076 | 0,478929685 | 0,54317635 |
| HUS00209434 | GtRNA\|GtRNA | chr6\|chr6 | Homo_sapiens | tRNA | trna149-LysTTT | trna149-LysTTT | GCCTGGGTAGCTCAGTCGGTAGAGCATCAGACTTTTAATCTGAGGGTCCAGGGTTCAAGTCCCTGTCCAGGCG | 88 | 251 | 72 | 87 | 120,6348669 | 0,390256251 | 0,564115864 | 0,691801589 | 0,489061938 | 0,553318251 |
| HUS00288844 | Ensembl | ENST00000365633 | Homo_sapiens | snoRNA | ENSG00000202503 | SNORD34 | TGGCGTCCATGATGTTCCGCAACTACCTACATTGTTTGATCCTCATGAAAGCAGCACTGGCTGAGACGCCA | 234 | 156 | 59 | 55 | 106,3232777 | -0,246421398 | 0,360943643 | -0,682714333 | 0,494787371 | 0,558437203 |
| HUS00084780 | Ensembl | ENST00000585078 | Homo_sapiens | snoRNA | ENSG00000264994 | SNORD92 | GTGCTGTGATGATGCCTTAATATTGTGGTTTCGACTCACTGAGAGTAAAATGAGGACCTACAATTCCTTGGCTGTGTCTGAGCAC | 2714 | 1561 | 457 | 692 | 1135,35564 | -0,310170946 | 0,456480037 | -0,679484142 | 0,496831152 | 0,559386164 |
| HUS00044086 | PiRNA | DQ600951.1 | Homo_sapiens | piRNA | piR-39017 | piR-39017 | TAGAGCATGAGACTCTTAATCTCAGGGTCGT | 290 | 141 | 52 | 137 | 148,8847701 | 0,401684209 | 0,59427222 | 0,675926276 | 0,499087454 | 0,560569242 |
| HUS00342424 | PiRNA | DQ593356.1 | Homo_sapiens | piRNA | piR-33468 | piR-33468 | CGGCTAGCTCAGTCGGTAGAGCATGAGACT | 4556 | 4057 | 538 | 1592 | 2251,750945 | -0,381142769 | 0,579778961 | -0,657393239 | 0,510928097 | 0,5724857 |
| HUS00199016 | GtRNA\|GtRNA | chr6\|chr6 | Homo_sapiens | tRNA | trna143-LysTTT | trna143-LysTTT | GCCTGGATAGCTCAGTCGGTAGAGCATCAGACTTTTAATCTGAGGGTCCAGGGTTCAAGTCCCTGTTCAGGCG | 101 | 301 | 78 | 107 | 142,181956 | 0,371873267 | 0,585192002 | 0,635472231 | 0,525120472 | 0,586973605 |
| HUS00175622 | MiRBase | MIMAT0004703 | Homo_sapiens | mature_miRNA | hsa-miR-335-3p | hsa-miR-335-3p | TTTTTCATTATTGCTCCTGACC | 536 | 770 | 205 | 346 | 440,1992945 | 0,295990571 | 0,480086885 | 0,616535422 | 0,537541201 | 0,597982436 |
| HUS00073883 | Ensembl\|Ensembl\|Ensembl\|Ensembl\|Ensembl\|Ensembl\|Ensembl\|Ensembl | ENST00000579412\|ENST00000582702\|ENST00000581427\|ENST00000364915\|ENST00000584949\|ENST00000578360\|ENST00000583420\|ENST00000580417 | Homo_sapiens | snoRNA | ENSG00000265116 | SNORD117 | GCCAAATGATGTTTATTTGAAACAGGAGCACCTCAGTGCAAGGACGACTCTTATCTATCACCCATGACTGATGGCT | 772 | 841 | 373 | 279 | 515,5463339 | 0,182582991 | 0,295782859 | 0,617287261 | 0,537045268 | 0,597982436 |
| HUS00202853 | Ensembl | ENST00000411292 | Homo_sapiens | snoRNA | ENSG00000223224 | SNORD71 | TGTGTGTTGGAGGATGAAAGTACGGAGTGATCCATCGGCTAAGTGTCTTGTCACAATGCTGACACTCAAACTGCTGACAGCACACG | 2012 | 3997 | 1017 | 789 | 1671,950914 | -0,249346977 | 0,44352166 | -0,56219797 | 0,573981145 | 0,636995781 |
| HUS00348518 | MiRBase | MIMAT0004505 | Homo_sapiens | mature_miRNA | hsa-miR-32-3p | hsa-miR-32-3p | CAATTTAGTGTGTGTGATATTT | 77 | 82 | 30 | 19 | 44,17214722 | -0,20569773 | 0,375397224 | -0,547946859 | 0,583728392 | 0,64627072 |
| HUS00002935 | MiRBase | MIMAT0000435 | Homo_sapiens | mature_miRNA | hsa-miR-143-3p | hsa-miR-143-3p | TGAGATGAAGCACTGTAGCTC | 79 | 62 | 37 | 21 | 44,9230918 | 0,20228234 | 0,376990698 | 0,536571171 | 0,591563862 | 0,653390014 |
| HUS00312965 | MiRBase | MIMAT0005882 | Homo_sapiens | mature_miRNA | hsa-miR-548k | hsa-miR-548k | AAAAGTACTTGCGGATTTTGCT | 252 | 167 | 71 | 94 | 133,9071822 | 0,201815733 | 0,409150103 | 0,493255976 | 0,621831731 | 0,683573888 |
| HUS00148000 | GtRNA\|GtRNA | chr16\|chr16 | Homo_sapiens | tRNA | trna10-LysCTT | trna10-LysCTT | GCCCGGCTAGCTCAGTCGGTAGAGCATGGGACTCTTAATCTCAGGGTCGTGGGTTCGAGCCCCACGTTGGGCG | 299 | 3166 | 766 | 1244 | 1423,466237 | 0,536627185 | 1,086832494 | 0,493753351 | 0,621480382 | 0,683573888 |
| HUS00010900 | MiRBase | MIMAT0022696 | Homo_sapiens | mature_miRNA | hsa-miR-301a-5p | hsa-miR-301a-5p | GCTCTGACTTTATTGCACTACT | 181 | 204 | 91 | 63 | 122,1028954 | 0,161708136 | 0,331429894 | 0,487910533 | 0,625613214 | 0,686108831 |
| HUS00106020 | Ensembl | ENST00000391250 | Homo_sapiens | snoRNA | ENSG00000212552 | SNORD91B | CTTAAGAGCCAATGATGTTTTTATTCAAAATGTCTGAACCTGTCTGAAGCATCCCAGTGATGCAACTTCTGTGTGATACTGAGGCTTTTTTGCCA | 590 | 143 | 87 | 111 | 192,5367376 | -0,288194935 | 0,609189873 | -0,473078999 | 0,636156819 | 0,696030402 |
| HUS00009287 | MiRBase | MIMAT0004550 | Homo_sapiens | mature_miRNA | hsa-miR-30c-2-3p | hsa-miR-30c-2-3p | CTGGGAGAAGGCTGTTTACTCT | 112 | 111 | 31 | 38 | 63,22333693 | -0,162928248 | 0,379082082 | -0,429796754 | 0,667343494 | 0,728438321 |
| HUS00075510 | MiRBase | MIMAT0004568 | Homo_sapiens | mature_miRNA | hsa-miR-221-5p | hsa-miR-221-5p | ACCTGGCATACAATGTAGATTT | 1559 | 1259 | 681 | 417 | 874,8846887 | 0,122514224 | 0,310239581 | 0,394901977 | 0,692915231 | 0,754579818 |
| HUS00240268 | MiRBase | MIMAT0000419 | Homo_sapiens | mature_miRNA | hsa-miR-27b-3p | hsa-miR-27b-3p | TTCACAGTGGCTAAGTTCTGC | 34566 | 36476 | 13512 | 13402 | 22143,51367 | 0,116145484 | 0,304921802 | 0,380902523 | 0,70327558 | 0,762993211 |
| HUS00248747 | MiRBase | MIMAT0000084 | Homo_sapiens | mature_miRNA | hsa-miR-27a-3p | hsa-miR-27a-3p | TTCACAGTGGCTAAGTTCCGC | 95118 | 90196 | 47040 | 25781 | 57686,98259 | 0,119144851 | 0,313513912 | 0,380030508 | 0,703922769 | 0,762993211 |
| HUS00030082 | GtRNA\|GtRNA | chr6\|chr6 | Homo_sapiens | tRNA | trna51-SerTGA | trna51-SerTGA | GTAGTCGTGGCCGAGTGGTTAAGGCGATGGACTTGAAATCCATTGGGGTTTCCCCGCGCAGGTTCGAATCCTGCCGACTACG | 119 | 185 | 69 | 52 | 96,75415166 | 0,149815338 | 0,400876483 | 0,37371945 | 0,708613053 | 0,764512923 |
| HUS00203086 | GtRNA\|GtRNA | chr6\|chr6 | Homo_sapiens | tRNA | trna147-SerAGA | trna147-SerAGA | GTAGTCGTGGCCGAGTGGTTAAGGCGATGGACTAGAAATCCATTGGGGTTTCCCCGCGCAGGTTCGAATCCTGCCGACTACG | 119 | 185 | 69 | 52 | 96,75415166 | 0,149815338 | 0,400876483 | 0,37371945 | 0,708613053 | 0,764512923 |
| HUS00261584 | MiRBase | MIMAT0000431 | Homo_sapiens | mature_miRNA | hsa-miR-140-5p | hsa-miR-140-5p | CAGTGGTTTTACCCTATGGTAG | 735 | 484 | 296 | 99 | 338,9227524 | -0,174734862 | 0,477880623 | -0,365645421 | 0,714629677 | 0,767979878 |
| HUS00338982 | GtRNA\|GtRNA\|GtRNA\|GtRNA\|GtRNA\|GtRNA\|GtRNA\|GtRNA\|GtRNA\|GtRNA | chr1\|chr16\|chr5\|chr5\|chr6\|chr1\|chr16\|chr5\|chr5\|chr6 | Homo_sapiens | tRNA | trna119-LysCTT | trna119-LysCTT | GCCCGGCTAGCTCAGTCGGTAGAGCATGAGACTCTTAATCTCAGGGTCGTGGGTTCGAGCCCCACGTTGGGCG | 848 | 12829 | 2521 | 4665 | 5316,625089 | 0,413865849 | 1,133954965 | 0,36497556 | 0,71512965 | 0,767979878 |
| HUS00130828 | MiRBase | MIMAT0003885 | Homo_sapiens | mature_miRNA | hsa-miR-454-3p | hsa-miR-454-3p | TAGTGCAATATTGCTTATAGGGT | 319 | 421 | 188 | 55 | 208,115137 | -0,183560306 | 0,512138562 | -0,358419225 | 0,720029607 | 0,771460293 |
| HUS00244741 | Ensembl | ENST00000386745 | Homo_sapiens | snoRNA | ENSG00000209480 | SNORD83B | GCTGTTCAGTGATGAGGCCTGGAATGTGCGCTGGGCACAGCGCCCGAGACAGACTGCGGAACCGTTCCTTGTTGCCTTCCTTCTGAGAACAGC | 559 | 473 | 156 | 232 | 323,8062544 | 0,142496617 | 0,411133961 | 0,34659413 | 0,728896257 | 0,777377889 |
| HUS00235996 | MiRBase | MIMAT0004955 | Homo_sapiens | mature_miRNA | hsa-miR-374b-5p | hsa-miR-374b-5p | ATATAATACAACCTGCTAAGTG | 1774 | 2429 | 1288 | 471 | 1341,525741 | 0,161891153 | 0,466246234 | 0,347222437 | 0,728424216 | 0,777377889 |
| HUS00295291 | MiRBase | MIMAT0000258 | Homo_sapiens | mature_miRNA | hsa-miR-181c-5p | hsa-miR-181c-5p | AACATTCAACCTGTCGGTGAGT | 827 | 406 | 260 | 137 | 344,7048112 | -0,142773949 | 0,437822694 | -0,326099927 | 0,744348752 | 0,792041578 |
| HUS00000020 | MiRBase | MIMAT0002809 | Homo_sapiens | mature_miRNA | hsa-miR-146b-5p | hsa-miR-146b-5p | TGAGAACTGAATTCCATAGGCT | 1309 | 794 | 338 | 330 | 596,2455688 | -0,118263298 | 0,365845851 | -0,323259914 | 0,746498409 | 0,792515435 |
| HUS00359261 | MiRBase | MIMAT0018927 | Homo_sapiens | mature_miRNA | hsa-miR-378e | hsa-miR-378e | ACTGGACTTGGAGTCAGGA | 148 | 66 | 43 | 25 | 59,55995616 | -0,146888428 | 0,466689273 | -0,31474567 | 0,752954765 | 0,795736286 |
| HUS00011999 | MiRBase | MIMAT0004795 | Homo_sapiens | mature_miRNA | hsa-miR-574-5p | hsa-miR-574-5p | TGAGTGTGTGTGTGTGAGTGTGT | 326 | 516 | 95 | 162 | 240,695273 | -0,156190405 | 0,495628081 | -0,315136311 | 0,75265816 | 0,795736286 |
| HUS00297603 | MiRBase | MIMAT0000096 | Homo_sapiens | mature_miRNA | hsa-miR-98-5p | hsa-miR-98-5p | TGAGGTAGTAAGTTGTATTGTT | 14696 | 14802 | 6260 | 3633 | 8501,586068 | -0,102399675 | 0,340888197 | -0,300390792 | 0,763879087 | 0,805450738 |
| HUS00305224 | MiRBase | MIMAT0000222 | Homo_sapiens | mature_miRNA | hsa-miR-192-5p | hsa-miR-192-5p | CTGACCTATGAATTGACAGCC | 1850 | 1272 | 472 | 680 | 966,925663 | 0,122115375 | 0,420780095 | 0,290211863 | 0,771654162 | 0,811808111 |
| HUS00115329 | MiRBase | MIMAT0001620 | Homo_sapiens | mature_miRNA | hsa-miR-200a-5p | hsa-miR-200a-5p | CATCTTACCGGACAGTGCTGGA | 3246 | 4585 | 964 | 1491 | 2258,272755 | -0,12148976 | 0,451875283 | -0,268856839 | 0,788039852 | 0,827175014 |
| HUS00014488 | MiRBase | MIMAT0001080 | Homo_sapiens | mature_miRNA | hsa-miR-196b-5p | hsa-miR-196b-5p | TAGGTAGTTTCCTGTTGTTGGG | 2812 | 2567 | 1371 | 466 | 1539,711601 | -0,113484535 | 0,455732606 | -0,249015615 | 0,803348703 | 0,841344925 |
| HUS00060594 | Ensembl | ENST00000391232 | Homo_sapiens | snoRNA | ENSG00000212534 | SNORD70 | TTCGTTGTTGTCAATGATGTATTCTTCTTGGAACTGAATCTAAGTGATCTGACTCAATATTCGTCACTACCACTGAGACAACGATGAA | 563 | 103 | 135 | 72 | 181,6209214 | -0,161531392 | 0,656766806 | -0,245949385 | 0,805721414 | 0,841933612 |
| HUS00268487 | MiRBase | MIMAT0000093 | Homo_sapiens | mature_miRNA | hsa-miR-93-5p | hsa-miR-93-5p | CAAAGTGCTGTTCGTGCAGGTAG | 8698 | 12736 | 5428 | 1926 | 6189,361419 | -0,118390054 | 0,495499623 | -0,238930665 | 0,811159347 | 0,845326217 |
| HUS00121420 | GtRNA\|GtRNA\|GtRNA\|GtRNA | chr14\|chr15\|chr14\|chr15 | Homo_sapiens | tRNA | trna13-LysCTT | trna13-LysCTT | GCCCGGCTAGCTCAGTCGGTAGAGCATGGGACTCTTAATCCCAGGGTCGTGGGTTCGAGCCCCACGTTGGGCG | 2419 | 133213 | 24451 | 41795 | 50444,96013 | 0,287033753 | 1,210764665 | 0,237068161 | 0,812603912 | 0,845326217 |
| HUS00090028 | MiRBase | MIMAT0022727 | Homo_sapiens | mature_miRNA | hsa-miR-1307-5p | hsa-miR-1307-5p | TCGACCGGACCTCGACCGGCT | 748 | 428 | 274 | 168 | 357,0689392 | 0,081694352 | 0,381072859 | 0,214379876 | 0,830250833 | 0,861755887 |
| HUS00121119 | MiRBase | MIMAT0004586 | Homo_sapiens | mature_miRNA | hsa-miR-15b-3p | hsa-miR-15b-3p | CGAATCATTATTTGCTGCTCTA | 661 | 605 | 326 | 157 | 386,4610557 | 0,069803494 | 0,363878514 | 0,191831864 | 0,84787391 | 0,87808768 |
| HUS00275841 | MiRBase | MIMAT0000318 | Homo_sapiens | mature_miRNA | hsa-miR-200b-3p | hsa-miR-200b-3p | TAATACTGCCTGGTAATGATGA | 89596 | 60493 | 36808 | 19451 | 45379,57238 | 0,059601957 | 0,349083438 | 0,170738427 | 0,864429446 | 0,893243761 |
| HUS00189290 | MiRBase | MIMAT0000065 | Homo_sapiens | mature_miRNA | hsa-let-7d-5p | hsa-let-7d-5p | AGAGGTAGTAGGTTGCATAGTT | 18732 | 15418 | 6852 | 5530 | 10309,57555 | 0,043316595 | 0,306617299 | 0,141272508 | 0,887654667 | 0,915209358 |
| HUS00194813 | Rfam | RF01290 | Homo_sapiens | snoRNA | SNORD10 | SNORD10 | GCTCTGTGATGGAGCCCATGCGTGTCATCTGAGCCTCTGGCTTCCCTGCCAGTGC | 282 | 345 | 192 | 54 | 191,9977425 | 0,061557232 | 0,514776515 | 0,119580498 | 0,904815469 | 0,924701524 |
| HUS00328928 | MiRBase | MIMAT0000266 | Homo_sapiens | mature_miRNA | hsa-miR-205-5p | hsa-miR-205-5p | TCCTTCATTCCACCGGAGTCTG | 2108 | 2097 | 922 | 550 | 1238,322761 | -0,038549919 | 0,308213723 | -0,125075284 | 0,90046395 | 0,924701524 |
| HUS00273754 | Ensembl | ENST00000384550 | Homo_sapiens | snoRNA | ENSG00000207280 | SNORD20 | TGGATATGATGACTGATTACCTGAGAAATAATTGATGAAATCTCAAGAAAATTCCTCTAGATAGTCAAGTTCTGATCCAG | 2061 | 4019 | 667 | 1301 | 1798,149514 | -0,066886662 | 0,552098408 | -0,121149892 | 0,903572312 | 0,924701524 |
| HUS00362011 | Ensembl | ENST00000626963 | Homo_sapiens | snoRNA | ENSG00000281147 | SNORD50A | TATCTGTGATGATCTTATCCCGAACCTGAACTTCTGTTGAAAAAAAAAAACTTTTACGGATCTGGCTTCTGAGAT | 15242 | 7367 | 3646 | 3801 | 6521,053166 | -0,053036843 | 0,439489298 | -0,120678349 | 0,903945809 | 0,924701524 |
| HUS00003302 | Ensembl | ENST00000384365 | Homo_sapiens | snoRNA | ENSG00000207093 | SNORD116-8 | GGATCGATGATGAGTCCTCCAAAAAAACATTCCTTGGAAAAGCTGAACAAAATGAGTGAGAACTCATACCGTCGTTCTCATCGGAACTGAGGTCC | 274 | 356 | 116 | 112 | 192,0720729 | 0,039978414 | 0,358590382 | 0,111487691 | 0,911229626 | 0,929214422 |
| HUS00322329 | MiRBase | MIMAT0004501 | Homo_sapiens | mature_miRNA | hsa-miR-27a-5p | hsa-miR-27a-5p | AGGGCTTAGCTGCTTGTGAGCA | 1484 | 1977 | 469 | 681 | 1024,118024 | -0,044421464 | 0,434684896 | -0,102192334 | 0,918604013 | 0,934684608 |
| HUS00287119 | Rfam\|Ensembl | RF00611\|ENST00000408139 | Homo_sapiens | snoRNA | SNORD111 | SNORD111 | CAGCCTGAAATGATGACTCTTTAAAAAATTTCATGTCTCTTCTCTGACATTTTTCTCTGGACACAGTTTTTGCCTTATGAATCTGATCAGGCTG | 251 | 400 | 111 | 113 | 194,2683134 | -0,032428564 | 0,411902453 | -0,078728746 | 0,937248381 | 0,948970162 |
| HUS00025602 | MiRBase | MIMAT0005794 | Homo_sapiens | mature_miRNA | hsa-miR-1296-5p | hsa-miR-1296-5p | TTAGGGCCCTGGCTCCATCTCC | 429 | 391 | 141 | 149 | 246,8362984 | 0,025306353 | 0,325277023 | 0,077799387 | 0,937987634 | 0,948970162 |
| HUS00041672 | Rfam | RF00334 | Homo_sapiens | snoRNA | SNORA3 | SNORA3 | ATCGAGGCTAGAGTCACGCTTGGGTATCGGCTATTGCCTGAGTGTGCTAGAGTCCTCGAAGAGTAACTGCTGACCTTA | 328 | 1060 | 385 | 156 | 431,608549 | 0,046823387 | 0,609515222 | 0,076820702 | 0,938766182 | 0,948970162 |
| HUS00152146 | MiRBase | MIMAT0017997 | Homo_sapiens | mature_miRNA | hsa-miR-3617-5p | hsa-miR-3617-5p | AAAGACATAGTTGCAAGATGGG | 93 | 76 | 43 | 19 | 50,38662356 | 0,025496278 | 0,399792436 | 0,063773789 | 0,949150349 | 0,957385927 |
| HUS00304927 | MiRBase | MIMAT0003880 | Homo_sapiens | mature_miRNA | hsa-miR-671-5p | hsa-miR-671-5p | AGGAAGCCCTGGAGGGGCTGGAG | 126 | 57 | 41 | 23 | 53,30576872 | -0,016147075 | 0,465864462 | -0,034660457 | 0,972350493 | 0,976550711 |
| HUS00300548 | MiRBase | MIMAT0000085 | Homo_sapiens | mature_miRNA | hsa-miR-28-5p | hsa-miR-28-5p | AAGGAGCTCACAGTCTATTGAG | 3463 | 2208 | 1459 | 623 | 1685,167975 | 0,015081153 | 0,421451166 | 0,035783869 | 0,971454696 | 0,976550711 |
| HUS00323553 | MiRBase | MIMAT0000075 | Homo_sapiens | mature_miRNA | hsa-miR-20a-5p | hsa-miR-20a-5p | TAAAGTGCTTATAGTGCAGGTAG | 18300 | 19267 | 9703 | 4184 | 11267,53395 | 0,009315795 | 0,426717803 | 0,021831279 | 0,982582543 | 0,984700178 |
| HUS00102309 | Ensembl | ENST00000383953 | Homo_sapiens | snoRNA | ENSG00000206680 | SNORD21 | GCTGAATGATGATATCCCACTAACTGAGCAGTCAGTAGTTGGTCCTTTGGTTGCATATGATGCGATAATTGTTTCAAGACGGGACTGATGGCAGC | 1974 | 2033 | 1138 | 354 | 1195,635067 | -0,002887556 | 0,483364604 | -0,005973868 | 0,995233571 | 0,995233571 |
