# Supplementary material for: miR-10a-5p and miR-29b-3p as Extracellular Vesicle-Associated Prostate Cancer Detection Markers
Source: Cancers (Basel). 2019 Dec 21;12(1):43. doi: 10.3390/cancers12010043 (PMC7017198; doi:10.3390/cancers12010043)
Supplement: Supplementary file 1 [file cancers-12-00043-s001.zip › cancers-672708-suppl-XML/cancers-672708-Table S2.docx]

| **ncRNA_ID** | **Source_DB** | **Source_ID** | **Organism** | **ncRNA_type** | **ncRNA_Symbol** | **Familiy** | **ncRNA_Sequence** | **PC3_Cells_1** | **PC3_Cells_2** | **PC3_EV_1** | **PC3_EV_2** | **baseMean** | **log2FoldChange** | **lfcSE** | **stat** | **p-value** | **FDR adj p-value** |
| --- | --- | --- | --- | --- | --- | --- | --- | --- | --- | --- | --- | --- | --- | --- | --- | --- | --- |
| HUS00024472 | MiRBase | MIMAT0004484 | Homo_sapiens | mature_miRNA | hsa-let-7d-3p | let7 | CTATACGACCTGCTGCCTTTCT | 257 | 129 | 2756 | 2270 | 2183,794182 | 5,093968698 | 0,38946735 | 13,07932152 | 4,32E-39 | 1,34E-37 |
| HUS00272786 | MiRBase | MIMAT0003218 | Homo_sapiens | mature_miRNA | hsa-miR-92b-3p | 25 | TATTGCACTCGTCCCGGCCTCC | 287 | 342 | 1628 | 1299 | 1330,526916 | 3,655421725 | 0,307413761 | 11,89088513 | 1,32E-32 | 3,61E-31 |
| HUS00035811 | MiRBase | MIMAT0017982 | Homo_sapiens | mature_miRNA | hsa-miR-3605-3p | 3605 | CCTCCGTGTTACCTGTCCTCTAG | 4 | 7 | 197 | 126 | 136,2181404 | 6,058592463 | 0,510044908 | 11,87854711 | 1,53E-32 | 3,95E-31 |
| HUS00283325 | MiRBase | MIMAT0000689 | Homo_sapiens | mature_miRNA | hsa-miR-99b-5p | 10 | CACCCGTAGAACCGACCTTGCG | 12541 | 10826 | 48275 | 59016 | 50019,25791 | 3,670431453 | 0,341005489 | 10,76355533 | 5,12E-27 | 7,93E-26 |
| HUS00006456 | MiRBase | MIMAT0005792 | Homo_sapiens | mature_miRNA | hsa-miR-320b | 320 | AAAAGCTGGGTTGAGAGGGCAA | 555 | 672 | 3352 | 1897 | 2354,404883 | 3,491406425 | 0,342846399 | 10,18358787 | 2,35E-24 | 3,12E-23 |
| HUS00147596 | MiRBase | MIMAT0004808 | Homo_sapiens | mature_miRNA | hsa-miR-625-3p | 625 | GACTATAGAACTTTCCCCCTCA | 66 | 89 | 336 | 306 | 296,7198237 | 3,479171704 | 0,356811456 | 9,750728701 | 1,83E-22 | 2,13E-21 |
| HUS00196652 | MiRBase | MIMAT0005923 | Homo_sapiens | mature_miRNA | hsa-miR-1269a | 1269 | CTGGACTGAGCCGTGCTACTGG | 255 | 182 | 787 | 504 | 602,5986627 | 3,000007028 | 0,327100569 | 9,171512723 | 4,66E-20 | 4,09E-19 |
| HUS00172894 | MiRBase | MIMAT0004774 | Homo_sapiens | mature_miRNA | hsa-miR-501-3p | 500 | AATGCACCCGGGCAAGGATTCT | 279 | 189 | 812 | 561 | 643,9633832 | 2,997974611 | 0,327514648 | 9,153711539 | 5,50E-20 | 4,74E-19 |
| HUS00099216 | MiRBase | MIMAT0000443 | Homo_sapiens | mature_miRNA | hsa-miR-125a-5p | 10 | TCCCTGAGACCCTTTAACCTGTGA | 7115 | 3950 | 33369 | 17797 | 22712,71417 | 3,594022051 | 0,399531541 | 8,995590301 | 2,35E-19 | 1,95E-18 |
| HUS00213419 | MiRBase | MIMAT0004482 | Homo_sapiens | mature_miRNA | hsa-let-7b-3p | let7 | CTATACAACCTACTGCCTTCCC | 34 | 17 | 143 | 103 | 110,7356844 | 3,670445973 | 0,408634934 | 8,98221289 | 2,65E-19 | 2,16E-18 |
| HUS00364807 | MiRBase | MIMAT0000751 | Homo_sapiens | mature_miRNA | hsa-miR-330-3p | 330 | GCAAAGCACACGGCCTGCAGAGA | 252 | 232 | 724 | 459 | 564,6734719 | 2,730077592 | 0,304171736 | 8,975447983 | 2,82E-19 | 2,26E-18 |
| HUS00045094 | MiRBase | MIMAT0005793 | Homo_sapiens | mature_miRNA | hsa-miR-320c | 320 | AAAAGCTGGGTTGAGAGGGT | 277 | 340 | 1512 | 804 | 1046,640726 | 3,291294423 | 0,367304408 | 8,960672263 | 3,23E-19 | 2,54E-18 |
| HUS00120793 | MiRBase | MIMAT0004984 | Homo_sapiens | mature_miRNA | hsa-miR-941 | 941 | CACCCGGCTGTGTGCACATGTGC | 157 | 177 | 432 | 391 | 400,3737855 | 2,762397727 | 0,316560416 | 8,726289151 | 2,63E-18 | 1,94E-17 |
| HUS00120931 | MiRBase | MIMAT0000449 | Homo_sapiens | mature_miRNA | hsa-miR-146a-5p | 146 | TGAGAACTGAATTCCATGGGTT | 761 | 455 | 6873 | 2352 | 3888,297927 | 4,195691956 | 0,483777924 | 8,672764394 | 4,22E-18 | 3,06E-17 |
| HUS00043489 | MiRBase | MIMAT0005825 | Homo_sapiens | mature_miRNA | hsa-miR-1180-3p | 1180 | TTTCCGGCTCGCGTGGGTGTGT | 341 | 237 | 773 | 623 | 675,5792112 | 2,738495934 | 0,315844096 | 8,670404059 | 4,31E-18 | 3,08E-17 |
| HUS00047103 | MiRBase | MIMAT0004614 | Homo_sapiens | mature_miRNA | hsa-miR-193a-5p | 193 | TGGGTCTTTGCGGGCGAGATGA | 322 | 241 | 923 | 554 | 696,412929 | 2,824173682 | 0,329511145 | 8,570798656 | 1,03E-17 | 7,24E-17 |
| HUS00104947 | MiRBase | MIMAT0022717 | Homo_sapiens | mature_miRNA | hsa-miR-873-3p | 873 | GGAGACTGATGAGTTCCCGGGA | 14 | 17 | 74 | 59 | 60,81962028 | 3,498154453 | 0,408713433 | 8,558941712 | 1,14E-17 | 7,91E-17 |
| HUS00195952 | MiRBase | MIMAT0005899 | Homo_sapiens | mature_miRNA | hsa-miR-1247-5p | 1247 | ACCCGTCCCGTTCGTCCCCGGA | 27 | 27 | 90 | 98 | 89,03313079 | 3,238489602 | 0,392399567 | 8,253040709 | 1,54E-16 | 9,97E-16 |
| HUS00014348 | MiRBase | MIMAT0005898 | Homo_sapiens | mature_miRNA | hsa-miR-1246 | 1246 | AATGGATTTTTGGAGCAGG | 2380 | 2637 | 175 | 124 | 876,1081783 | -2,540340845 | 0,309521484 | -8,20731671 | 2,26E-16 | 1,42E-15 |
| HUS00224414 | MiRBase | MIMAT0004980 | Homo_sapiens | mature_miRNA | hsa-miR-937-3p | 937 | ATCCGCGCTCTGACTCTCTGCC | 32 | 20 | 107 | 149 | 119,6146522 | 3,720187181 | 0,457009462 | 8,140284804 | 3,94E-16 | 2,38E-15 |
| HUS00088227 | MiRBase | MIMAT0000102 | Homo_sapiens | mature_miRNA | hsa-miR-105-5p | 105 | TCAAATGCTCAGACTCCTGTGGT | 21 | 25 | 80 | 66 | 68,68892823 | 3,08871124 | 0,383530257 | 8,053370463 | 8,05E-16 | 4,80E-15 |
| HUS00187563 | MiRBase | MIMAT0003322 | Homo_sapiens | mature_miRNA | hsa-miR-652-3p | 652 | AATGGCGCCACTAGGGTTGTG | 307 | 284 | 658 | 437 | 545,2955404 | 2,342740233 | 0,294171209 | 7,963866499 | 1,67E-15 | 9,81E-15 |
| HUS00334425 | MiRBase | MIMAT0000279 | Homo_sapiens | mature_miRNA | hsa-miR-222-3p | 221 | AGCTACATCTGGCTACTGGGT | 12949 | 9884 | 28290 | 17104 | 22233,5332 | 2,435981272 | 0,311271565 | 7,825903644 | 5,04E-15 | 2,73E-14 |
| HUS00149351 | MiRBase | MIMAT0000092 | Homo_sapiens | mature_miRNA | hsa-miR-92a-3p | 25 | TATTGCACTTGTCCCGGCCTGT | 32602 | 37682 | 71161 | 72622 | 72203,82407 | 2,506556684 | 0,322933828 | 7,761827556 | 8,37E-15 | 4,47E-14 |
| HUS00310391 | MiRBase | MIMAT0000510 | Homo_sapiens | mature_miRNA | hsa-miR-320a | 320 | AAAAGCTGGGTTGAGAGGGCGA | 5180 | 5938 | 17762 | 10169 | 13222,32598 | 2,734426521 | 0,36188493 | 7,55606629 | 4,15E-14 | 2,17E-13 |
| HUS00311403 | MiRBase | MIMAT0004680 | Homo_sapiens | mature_miRNA | hsa-miR-130b-5p | 130 | ACTCTTTCCCTGTTGCACTAC | 95 | 60 | 206 | 243 | 217,2508544 | 2,999307038 | 0,397902673 | 7,537790622 | 4,78E-14 | 2,47E-13 |
| HUS00060652 | MiRBase | MIMAT0004748 | Homo_sapiens | mature_miRNA | hsa-miR-423-5p | 423 | TGAGGGGCAGAGAGCGAGACTTT | 1281 | 1518 | 4065 | 2197 | 3001,665826 | 2,569663247 | 0,347490783 | 7,394910524 | 1,42E-13 | 7,00E-13 |
| HUS00127643 | MiRBase | MIMAT0004502 | Homo_sapiens | mature_miRNA | hsa-miR-28-3p | 28 | CACTAGATTGTGAGCTCCTGGA | 1184 | 1319 | 2147 | 1770 | 2032,390805 | 2,116850869 | 0,286545413 | 7,387488246 | 1,50E-13 | 7,25E-13 |
| HUS00280833 | MiRBase | MIMAT0004485 | Homo_sapiens | mature_miRNA | hsa-let-7e-3p | let7 | CTATACGGCCTCCTAGCTTTCC | 42 | 31 | 87 | 72 | 78,13430492 | 2,576195961 | 0,358814983 | 7,179733518 | 6,98E-13 | 3,22E-12 |
| HUS00155913 | MiRBase | MIMAT0000450 | Homo_sapiens | mature_miRNA | hsa-miR-149-5p | 149 | TCTGGCTCCGTGTCTTCACTCCC | 315 | 279 | 549 | 363 | 469,1402831 | 2,076324395 | 0,295117891 | 7,035576143 | 1,98E-12 | 8,85E-12 |
| HUS00242017 | MiRBase | MIMAT0004672 | Homo_sapiens | mature_miRNA | hsa-miR-106b-3p | 17 | CCGCACTGTGGGTACTTGCTGC | 1210 | 1344 | 1939 | 1777 | 1965,456015 | 2,020281658 | 0,298272725 | 6,773269868 | 1,26E-11 | 5,18E-11 |
| HUS00024483 | MiRBase | MIMAT0000226 | Homo_sapiens | mature_miRNA | hsa-miR-196a-5p | 196 | TAGGTAGTTTCATGTTGTTGGG | 89 | 97 | 185 | 126 | 157,8836561 | 2,188414352 | 0,325525932 | 6,722703597 | 1,78E-11 | 7,28E-11 |
| HUS00076751 | MiRBase | MIMAT0017985 | Homo_sapiens | mature_miRNA | hsa-miR-3607-3p | 3607 | ACTGTAAACGCTTTCTGATG | 173 | 603 | 1 | 4 | 121,0578983 | -5,068759025 | 0,77336079 | -6,554197069 | 5,59E-11 | 2,19E-10 |
| HUS00250686 | MiRBase | MIMAT0000617 | Homo_sapiens | mature_miRNA | hsa-miR-200c-3p | 8 | TAATACTGCCGGGTAATGATGGA | 95 | 61 | 180 | 115 | 145,9441692 | 2,36131886 | 0,361029062 | 6,540522944 | 6,13E-11 | 2,38E-10 |
| HUS00196788 | MiRBase | MIMAT0010133 | Homo_sapiens | mature_miRNA | hsa-miR-2110 | 2110 | TTGGGGAAACGGCCGCTGAGTG | 27 | 15 | 56 | 82 | 66,70871848 | 3,147118331 | 0,481329719 | 6,538383585 | 6,22E-11 | 2,39E-10 |
| HUS00301195 | MiRBase | MIMAT0000074 | Homo_sapiens | mature_miRNA | hsa-miR-19b-3p | 19 | TGTGCAAATCCATGCAAAACTGA | 7418 | 6230 | 588 | 258 | 2374,278016 | -2,493618046 | 0,383385897 | -6,504198688 | 7,81E-11 | 2,93E-10 |
| HUS00149858 | MiRBase | MIMAT0000253 | Homo_sapiens | mature_miRNA | hsa-miR-10a-5p | 10 | TACCCTGTAGATCCGAATTTGTG | 15577 | 18945 | 26079 | 21157 | 25148,20217 | 1,919101041 | 0,298972285 | 6,418993115 | 1,37E-10 | 5,06E-10 |
| HUS00285163 | MiRBase | MIMAT0004569 | Homo_sapiens | mature_miRNA | hsa-miR-222-5p | 221 | CTCAGTAGCCAGTGTAGATCCT | 51 | 52 | 94 | 116 | 106,5336644 | 2,496346441 | 0,392065101 | 6,367173292 | 1,93E-10 | 6,94E-10 |
| HUS00256382 | MiRBase | MIMAT0000703 | Homo_sapiens | mature_miRNA | hsa-miR-361-5p | 361 | TTATCAGAATCTCCAGGGGTAC | 1451 | 1212 | 3567 | 1636 | 2521,0782 | 2,368191594 | 0,373001992 | 6,349005217 | 2,17E-10 | 7,75E-10 |
| HUS00181846 | MiRBase | MIMAT0000440 | Homo_sapiens | mature_miRNA | hsa-miR-191-5p | 191 | CAACGGAATCCCAAAAGCAGCTG | 36030 | 26499 | 66658 | 35236 | 51232,70989 | 2,137233004 | 0,337155563 | 6,339011546 | 2,31E-10 | 8,21E-10 |
| HUS00197467 | MiRBase | MIMAT0030019 | Homo_sapiens | mature_miRNA | hsa-miR-7704 | 7704 | CGGGGTCGGCGGCGACGTG | 41 | 27 | 71 | 96 | 82,94698413 | 2,756347904 | 0,436864823 | 6,309383952 | 2,80E-10 | 9,87E-10 |
| HUS00045571 | MiRBase | MIMAT0004810 | Homo_sapiens | mature_miRNA | hsa-miR-629-5p | 629 | TGGGTTTACGTTGGGAGAACT | 663 | 525 | 911 | 622 | 817,6345522 | 1,835012689 | 0,295490236 | 6,210062012 | 5,30E-10 | 1,85E-09 |
| HUS00133308 | MiRBase | MIMAT0000257 | Homo_sapiens | mature_miRNA | hsa-miR-181b-5p | 181 | AACATTCATTGCTGTCGGTGGGT | 616 | 554 | 1250 | 654 | 957,8813035 | 2,127154856 | 0,343370101 | 6,194933254 | 5,83E-10 | 2,02E-09 |
| HUS00338313 | MiRBase | MIMAT0000100 | Homo_sapiens | mature_miRNA | hsa-miR-29b-3p | 29 | TAGCACCATTTGAAATCAGTGTT | 3085 | 2138 | 250 | 206 | 966,3810773 | -1,969397793 | 0,319472316 | -6,16453349 | 7,07E-10 | 2,42E-09 |
| HUS00274539 | MiRBase | MIMAT0000752 | Homo_sapiens | mature_miRNA | hsa-miR-328-3p | 328 | CTGGCCCTCTCTGCCCTTCCGT | 219 | 87 | 432 | 382 | 390,8285679 | 2,842021633 | 0,461017225 | 6,164675595 | 7,06E-10 | 2,42E-09 |
| HUS00011838 | MiRBase | MIMAT0000423 | Homo_sapiens | mature_miRNA | hsa-miR-125b-5p | 10 | TCCCTGAGACCCTAACTTGTGA | 21637 | 13857 | 53872 | 22005 | 36038,43652 | 2,478765058 | 0,416899342 | 5,945715929 | 2,75E-09 | 9,01E-09 |
| HUS00316219 | MiRBase | MIMAT0000078 | Homo_sapiens | mature_miRNA | hsa-miR-23a-3p | 23 | ATCACATTGCCAGGGATTTCC | 22361 | 21706 | 49246 | 22653 | 35945,9631 | 2,1136564 | 0,357284809 | 5,915886558 | 3,30E-09 | 1,07E-08 |
| HUS00117841 | MiRBase | MIMAT0000097 | Homo_sapiens | mature_miRNA | hsa-miR-99a-5p | 10 | AACCCGTAGATCCGATCTTGTG | 351 | 324 | 455 | 521 | 522,2706071 | 2,025735944 | 0,344466297 | 5,880795773 | 4,08E-09 | 1,32E-08 |
| HUS00001691 | MiRBase | MIMAT0000256 | Homo_sapiens | mature_miRNA | hsa-miR-181a-5p | 181 | AACATTCAACGCTGTCGGTGAGT | 1735 | 1126 | 2815 | 1563 | 2231,240212 | 2,051267369 | 0,357764549 | 5,733568004 | 9,83E-09 | 3,13E-08 |
| HUS00042803 | MiRBase | MIMAT0022494 | Homo_sapiens | mature_miRNA | hsa-miR-5701 | 5701 | TTATTGTCACGTTCTGATT | 133 | 82 | 2 | 1 | 32,98645628 | -4,155715388 | 0,727891287 | -5,709252823 | 1,13E-08 | 3,59E-08 |
| HUS00104973 | MiRBase | MIMAT0000099 | Homo_sapiens | mature_miRNA | hsa-miR-101-3p | 101 | TACAGTACTGTGATAACTGAA | 18177 | 15282 | 1352 | 1520 | 6215,24655 | -1,965514957 | 0,347258229 | -5,660096126 | 1,51E-08 | 4,75E-08 |
| HUS00225878 | MiRBase | MIMAT0000418 | Homo_sapiens | mature_miRNA | hsa-miR-23b-3p | 23 | ATCACATTGCCAGGGATTACC | 3286 | 2951 | 9261 | 3617 | 6142,27681 | 2,410384983 | 0,432984319 | 5,566910577 | 2,59E-08 | 8,09E-08 |
| HUS00099652 | MiRBase | MIMAT0004558 | Homo_sapiens | mature_miRNA | hsa-miR-181a-2-3p | 181 | ACCACTGACCGTTGACTGTACC | 33 | 42 | 59 | 78 | 70,97211288 | 2,327674024 | 0,429258416 | 5,422547207 | 5,88E-08 | 1,77E-07 |
| HUS00240215 | MiRBase | MIMAT0006764 | Homo_sapiens | mature_miRNA | hsa-miR-320d | 320 | AAAAGCTGGGTTGAGAGGA | 209 | 249 | 662 | 260 | 442,0108749 | 2,366248387 | 0,438048247 | 5,401798553 | 6,60E-08 | 1,98E-07 |
| HUS00341222 | MiRBase | MIMAT0004682 | Homo_sapiens | mature_miRNA | hsa-miR-361-3p | 361 | TCCCCCAGGTGTGATTCTGATTT | 1640 | 1656 | 1873 | 2205 | 2257,101438 | 1,806111608 | 0,342913769 | 5,266955638 | 1,39E-07 | 4,01E-07 |
| HUS00136572 | MiRBase | MIMAT0004749 | Homo_sapiens | mature_miRNA | hsa-miR-424-3p | 322 | CAAAACGTGAGGCGCTGCTAT | 297 | 424 | 551 | 850 | 724,7862134 | 2,415225495 | 0,462066508 | 5,227008345 | 1,72E-07 | 4,88E-07 |
| HUS00010416 | MiRBase | MIMAT0000073 | Homo_sapiens | mature_miRNA | hsa-miR-19a-3p | 19 | TGTGCAAATCTATGCAAAACTGA | 2152 | 1301 | 178 | 81 | 615,5240952 | -2,197640528 | 0,42271171 | -5,198910921 | 2,00E-07 | 5,65E-07 |
| HUS00032315 | MiRBase | MIMAT0004571 | Homo_sapiens | mature_miRNA | hsa-miR-200b-5p | 8 | CATCTTACTGGGCAGCATTGGA | 599 | 305 | 708 | 704 | 737,678752 | 2,122703714 | 0,409997705 | 5,17735511 | 2,25E-07 | 6,27E-07 |
| HUS00147295 | MiRBase | MIMAT0001341 | Homo_sapiens | mature_miRNA | hsa-miR-424-5p | 322 | CAGCAGCAATTCATGTTTTGAA | 987 | 917 | 139 | 93 | 380,7058995 | -1,524681232 | 0,299224594 | -5,095440888 | 3,48E-07 | 9,63E-07 |
| HUS00059657 | MiRBase | MIMAT0004602 | Homo_sapiens | mature_miRNA | hsa-miR-125a-3p | 10 | ACAGGTGAGGTTCTTGGGAGCC | 67 | 76 | 98 | 159 | 134,7930008 | 2,317000926 | 0,455268447 | 5,089307065 | 3,59E-07 | 9,89E-07 |
| HUS00176719 | MiRBase | MIMAT0000459 | Homo_sapiens | mature_miRNA | hsa-miR-193a-3p | 193 | AACTGGCCTACAAAGTCCCAGT | 61 | 76 | 2 | 1 | 21,78631809 | -3,595131777 | 0,729833447 | -4,925961933 | 8,39E-07 | 2,23E-06 |
| HUS00324610 | MiRBase | MIMAT0005797 | Homo_sapiens | mature_miRNA | hsa-miR-1301-3p | 1301 | TTGCAGCTGCCTGGGAGTGACTTC | 278 | 178 | 280 | 239 | 287,542937 | 1,673365689 | 0,339844695 | 4,92391294 | 8,48E-07 | 2,24E-06 |
| HUS00003948 | MiRBase | MIMAT0004681 | Homo_sapiens | mature_miRNA | hsa-miR-26a-2-3p | 26 | CCTATTCTTGATTACTTGTTTC | 124 | 104 | 6 | 9 | 40,4924725 | -2,314456054 | 0,472187978 | -4,901556498 | 9,51E-07 | 2,48E-06 |
| HUS00210876 | MiRBase | MIMAT0022706 | Homo_sapiens | mature_miRNA | hsa-miR-561-5p | 561 | ATCAAGGATCTTAAACTTTGCC | 102 | 85 | 7 | 5 | 32,83726797 | -2,35693711 | 0,492901471 | -4,781761162 | 1,74E-06 | 4,37E-06 |
| HUS00293488 | MiRBase | MIMAT0004495 | Homo_sapiens | mature_miRNA | hsa-miR-22-5p | 22 | AGTTCTTCAGTGGCAAGCTTTA | 272 | 188 | 469 | 204 | 342,1708457 | 1,952639732 | 0,412893269 | 4,729163393 | 2,25E-06 | 5,61E-06 |
| HUS00151397 | MiRBase | MIMAT0000424 | Homo_sapiens | mature_miRNA | hsa-miR-128-3p | 128 | TCACAGTGAACCGGTCTCTTT | 6527 | 5211 | 6162 | 6556 | 7213,266308 | 1,617013376 | 0,343705338 | 4,704650173 | 2,54E-06 | 6,29E-06 |
| HUS00284741 | MiRBase | MIMAT0000681 | Homo_sapiens | mature_miRNA | hsa-miR-29c-3p | 29 | TAGCACCATTTGAAATCGGTTA | 434 | 262 | 41 | 22 | 128,7185744 | -1,917513523 | 0,414856442 | -4,622113405 | 3,80E-06 | 9,22E-06 |
| HUS00001657 | MiRBase | MIMAT0003247 | Homo_sapiens | mature_miRNA | hsa-miR-582-5p | 582 | TTACAGTTGTTCAACCAGTTACT | 93 | 167 | 9 | 6 | 45,58104547 | -2,504936065 | 0,542009806 | -4,621569644 | 3,81E-06 | 9,22E-06 |
| HUS00114818 | MiRBase | MIMAT0003239 | Homo_sapiens | mature_miRNA | hsa-miR-574-3p | 574 | CACGCTCATGCACACACCCACA | 165 | 143 | 498 | 140 | 299,8977416 | 2,362580052 | 0,511540989 | 4,618554723 | 3,86E-06 | 9,31E-06 |
| HUS00012939 | MiRBase | MIMAT0004678 | Homo_sapiens | mature_miRNA | hsa-miR-99b-3p | 10 | CAAGCTCGTGTCTGTGGGTCCG | 642 | 607 | 644 | 972 | 896,1907812 | 1,8733333 | 0,418010707 | 4,481543821 | 7,41E-06 | 1,76E-05 |
| HUS00327327 | MiRBase | MIMAT0001340 | Homo_sapiens | mature_miRNA | hsa-miR-423-3p | 423 | AGCTCGGTCTGAGGCCCCTCAGT | 8740 | 3680 | 10053 | 10122 | 10466,98995 | 2,158762303 | 0,484353034 | 4,457001712 | 8,31E-06 | 1,96E-05 |
| HUS00016157 | MiRBase | MIMAT0000090 | Homo_sapiens | mature_miRNA | hsa-miR-32-5p | 32 | TATTGCACATTACTAAGTTGCA | 3352 | 2429 | 419 | 192 | 1106,408056 | -1,732647807 | 0,390158637 | -4,440880304 | 8,96E-06 | 2,09E-05 |
| HUS00146483 | MiRBase | MIMAT0000728 | Homo_sapiens | mature_miRNA | hsa-miR-375 | 375 | TTTGTTCGTTCGGCTCGCGTGA | 17254 | 16143 | 15592 | 19756 | 20345,98613 | 1,589559167 | 0,367249031 | 4,328286887 | 1,50E-05 | 3,44E-05 |
| HUS00079963 | MiRBase | MIMAT0002174 | Homo_sapiens | mature_miRNA | hsa-miR-484 | 484 | TCAGGCTCAGTCCCCTCCCGAT | 683 | 289 | 1089 | 509 | 795,8497242 | 2,108629559 | 0,488477182 | 4,316741161 | 1,58E-05 | 3,59E-05 |
| HUS00256189 | MiRBase | MIMAT0004700 | Homo_sapiens | mature_miRNA | hsa-miR-331-5p | 331 | CTAGGTATGGTCCCAGGGATCC | 119 | 124 | 338 | 99 | 210,6681387 | 2,168837803 | 0,504335286 | 4,300388775 | 1,70E-05 | 3,85E-05 |
| HUS00182106 | MiRBase | MIMAT0004597 | Homo_sapiens | mature_miRNA | hsa-miR-140-3p | 140 | TACCACAGGGTAGAACCACGG | 467 | 359 | 403 | 305 | 420,6312975 | 1,263065168 | 0,295257928 | 4,277836597 | 1,89E-05 | 4,24E-05 |
| HUS00144940 | MiRBase | MIMAT0000275 | Homo_sapiens | mature_miRNA | hsa-miR-218-5p | 218 | TTGTGCTTGATCTAACCATGT | 7728 | 8824 | 1340 | 654 | 3295,897588 | -1,564083996 | 0,366705826 | -4,265228104 | 2,00E-05 | 4,46E-05 |
| HUS00065412 | MiRBase | MIMAT0000086 | Homo_sapiens | mature_miRNA | hsa-miR-29a-3p | 29 | TAGCACCATCTGAAATCGGTTA | 81062 | 47342 | 9682 | 6001 | 25463,66875 | -1,505746488 | 0,358196905 | -4,203683688 | 2,63E-05 | 5,79E-05 |
| HUS00330309 | MiRBase | MIMAT0000244 | Homo_sapiens | mature_miRNA | hsa-miR-30c-5p | 30 | TGTAAACATCCTACACTCTCAGC | 3742 | 2918 | 2912 | 2346 | 3210,879201 | 1,153677803 | 0,27443934 | 4,203762493 | 2,63E-05 | 5,79E-05 |
| HUS00183703 | MiRBase | MIMAT0004953 | Homo_sapiens | mature_miRNA | hsa-miR-873-5p | 873 | GCAGGAACTTGTGAGTCTCCT | 219 | 228 | 201 | 195 | 236,1232602 | 1,317904382 | 0,318031701 | 4,143940298 | 3,41E-05 | 7,42E-05 |
| HUS00300555 | MiRBase | MIMAT0017990 | Homo_sapiens | mature_miRNA | hsa-miR-3613-5p | 3613 | TGTTGTACTTTTTTTTTTGTTC | 669 | 449 | 61 | 68 | 221,0722036 | -1,541500032 | 0,375704491 | -4,102958757 | 4,08E-05 | 8,82E-05 |
| HUS00260724 | MiRBase | MIMAT0000088 | Homo_sapiens | mature_miRNA | hsa-miR-30a-3p | 30 | CTTTCAGTCGGATGTTTGCAGC | 494 | 390 | 391 | 447 | 493,2962393 | 1,432290928 | 0,349478994 | 4,098360568 | 4,16E-05 | 8,96E-05 |
| HUS00069196 | MiRBase | MIMAT0000691 | Homo_sapiens | mature_miRNA | hsa-miR-130b-3p | 130 | CAGTGCAATGATGAAAGGGCAT | 137 | 118 | 293 | 91 | 191,5829856 | 1,939904 | 0,487102286 | 3,982539305 | 6,82E-05 | 1,45E-04 |
| HUS00246844 | MiRBase | MIMAT0000071 | Homo_sapiens | mature_miRNA | hsa-miR-17-3p | 17 | ACTGCAGTGAAGGCACTTGTAG | 211 | 130 | 23 | 13 | 65,2488548 | -1,692094811 | 0,429522034 | -3,939483138 | 8,17E-05 | 1,73E-04 |
| HUS00135422 | MiRBase | MIMAT0000255 | Homo_sapiens | mature_miRNA | hsa-miR-34a-5p | 34 | TGGCAGTGTCTTAGCTGGTTGT | 183 | 252 | 31 | 19 | 86,18441737 | -1,598586528 | 0,412489876 | -3,875456395 | 1,06E-04 | 2,23E-04 |
| HUS00333348 | MiRBase | MIMAT0000091 | Homo_sapiens | mature_miRNA | hsa-miR-33a-5p | 33 | GTGCATTGTAGTTGCATTGCA | 201 | 162 | 26 | 21 | 73,79812051 | -1,410840141 | 0,365820089 | -3,856650261 | 1,15E-04 | 2,39E-04 |
| HUS00199023 | MiRBase | MIMAT0000245 | Homo_sapiens | mature_miRNA | hsa-miR-30d-5p | 30 | TGTAAACATCCCCGACTGGAAG | 38113 | 27963 | 30505 | 20239 | 30983,30551 | 1,097855319 | 0,285780061 | 3,841609233 | 1,22E-04 | 2,51E-04 |
| HUS00207745 | MiRBase | MIMAT0004555 | Homo_sapiens | mature_miRNA | hsa-miR-10a-3p | 10 | CAAATTCGTATCTAGGGGAATA | 324 | 301 | 574 | 208 | 408,4785619 | 1,702590395 | 0,443892402 | 3,835592561 | 1,25E-04 | 2,57E-04 |
| HUS00339081 | MiRBase | MIMAT0005951 | Homo_sapiens | mature_miRNA | hsa-miR-1307-3p | 1307 | ACTCGGCGTGGCGTCGGTCGTG | 1007 | 523 | 900 | 645 | 873,3312796 | 1,486250453 | 0,391437681 | 3,796901846 | 1,47E-04 | 2,96E-04 |
| HUS00124785 | MiRBase | MIMAT0004592 | Homo_sapiens | mature_miRNA | hsa-miR-125b-1-3p | 10 | ACGGGTTAGGCTCTTGGGAGCT | 1102 | 1382 | 1089 | 1060 | 1291,83075 | 1,277276362 | 0,337395851 | 3,785690776 | 1,53E-04 | 3,09E-04 |
| HUS00213960 | MiRBase | MIMAT0000693 | Homo_sapiens | mature_miRNA | hsa-miR-30e-3p | 30 | CTTTCAGTCGGATGTTTACAGC | 949 | 658 | 693 | 613 | 793,1548065 | 1,200956043 | 0,320533578 | 3,746740206 | 1,79E-04 | 3,59E-04 |
| HUS00286360 | MiRBase | MIMAT0004518 | Homo_sapiens | mature_miRNA | hsa-miR-16-2-3p | 15 | CCAATATTACTGTGCTGCTTTA | 364 | 249 | 264 | 253 | 311,6387693 | 1,257598483 | 0,336506095 | 3,737223489 | 1,86E-04 | 3,71E-04 |
| HUS00200246 | MiRBase | MIMAT0004801 | Homo_sapiens | mature_miRNA | hsa-miR-590-3p | 590 | TAATTTTATGTATAAGCTAGT | 285 | 283 | 48 | 16 | 110,4838374 | -1,645483797 | 0,444747033 | -3,699819616 | 2,16E-04 | 4,25E-04 |
| HUS00097169 | MiRBase | MIMAT0000250 | Homo_sapiens | mature_miRNA | hsa-miR-139-5p | 139 | TCTACAGTGCACGTGTCTCCAGT | 210 | 330 | 252 | 327 | 333,5131114 | 1,576247076 | 0,44023374 | 3,580477674 | 3,43E-04 | 6,73E-04 |
| HUS00022361 | MiRBase | MIMAT0001536 | Homo_sapiens | mature_miRNA | hsa-miR-429 | 8 | TAATACTGTCTGGTAAAACCGT | 63433 | 40568 | 9584 | 5870 | 21793,68179 | -1,23453973 | 0,345830984 | -3,569777689 | 3,57E-04 | 6,98E-04 |
| HUS00072747 | MiRBase | MIMAT0004775 | Homo_sapiens | mature_miRNA | hsa-miR-502-3p | 500 | AATGCACCTGGGCAAGGATTCA | 930 | 620 | 1089 | 492 | 874,5124936 | 1,451494483 | 0,407619471 | 3,560905668 | 3,70E-04 | 7,16E-04 |
| HUS00180924 | MiRBase | MIMAT0000068 | Homo_sapiens | mature_miRNA | hsa-miR-15a-5p | 15 | TAGCAGCACATAATGGTTTGTG | 231 | 161 | 34 | 17 | 78,98669218 | -1,417119828 | 0,402025415 | -3,524950849 | 4,24E-04 | 8,11E-04 |
| HUS00199736 | MiRBase | MIMAT0003339 | Homo_sapiens | mature_miRNA | hsa-miR-421 | 95 | ATCAACAGACATTAATTGGGCGC | 639 | 626 | 660 | 371 | 614,9127221 | 1,154941317 | 0,327641422 | 3,525016193 | 4,23E-04 | 8,11E-04 |
| HUS00129468 | MiRBase | MIMAT0000261 | Homo_sapiens | mature_miRNA | hsa-miR-183-5p | 183 | TATGGCACTGGTAGAATTCACT | 47277 | 52306 | 35978 | 35065 | 45287,64227 | 1,013307114 | 0,294013513 | 3,446464429 | 5,68E-04 | 1,07E-03 |
| HUS00215288 | MiRBase | MIMAT0000259 | Homo_sapiens | mature_miRNA | hsa-miR-182-5p | 182 | TTTGGCAATGGTAGAACTCACACT | 73174 | 81857 | 58527 | 51313 | 69872,44388 | 0,994185918 | 0,288821079 | 3,442220778 | 5,77E-04 | 1,09E-03 |
| HUS00085841 | MiRBase | MIMAT0005949 | Homo_sapiens | mature_miRNA | hsa-miR-664a-3p | 664 | TATTCATTTATCCCCAGCCTACA | 58 | 136 | 163 | 88 | 132,9847561 | 1,735613824 | 0,508320926 | 3,414405616 | 6,39E-04 | 1,20E-03 |
| HUS00154150 | MiRBase | MIMAT0003389 | Homo_sapiens | mature_miRNA | hsa-miR-542-3p | 542 | TGTGACAGATTGATAACTGAAA | 1842 | 2362 | 335 | 323 | 912,3995552 | -1,143732602 | 0,346853466 | -3,297451848 | 9,76E-04 | 1,81E-03 |
| HUS00351381 | MiRBase | MIMAT0003321 | Homo_sapiens | mature_miRNA | hsa-miR-651-5p | 651 | TTTAGGATAAGCTTGACTTTTG | 1215 | 1512 | 266 | 189 | 599,9195035 | -1,080007687 | 0,33027044 | -3,270070695 | 1,08E-03 | 1,99E-03 |
| HUS00226723 | MiRBase | MIMAT0022724 | Homo_sapiens | mature_miRNA | hsa-miR-1277-5p | 1277 | AAATATATATATATATGTACGTAT | 112 | 78 | 17 | 7 | 37,88098828 | -1,447355857 | 0,449220876 | -3,221924747 | 1,27E-03 | 2,34E-03 |
| HUS00027911 | MiRBase | MIMAT0003249 | Homo_sapiens | mature_miRNA | hsa-miR-584-5p | 584 | TTATGGTTTGCCTGGGACTGAG | 9526 | 8353 | 6951 | 5185 | 7765,517576 | 0,92630013 | 0,291980126 | 3,17247664 | 1,51E-03 | 2,76E-03 |
| HUS00310422 | MiRBase | MIMAT0002821 | Homo_sapiens | mature_miRNA | hsa-miR-181d-5p | 181 | AACATTCATTGTTGTCGGTGGGT | 2309 | 2093 | 2292 | 1143 | 2065,511005 | 1,082503954 | 0,347490701 | 3,11520265 | 1,84E-03 | 3,31E-03 |
| HUS00335130 | MiRBase | MIMAT0000439 | Homo_sapiens | mature_miRNA | hsa-miR-153-3p | 153 | TTGCATAGTCACAAAAGTGATC | 90 | 61 | 13 | 6 | 30,111477 | -1,442202656 | 0,464109109 | -3,107464661 | 1,89E-03 | 3,37E-03 |
| HUS00327920 | MiRBase | MIMAT0004799 | Homo_sapiens | mature_miRNA | hsa-miR-589-5p | 589 | TGAGAACCACGTCTGCTCTGAG | 162 | 181 | 132 | 112 | 154,7925474 | 0,993276756 | 0,320349686 | 3,10060162 | 1,93E-03 | 3,44E-03 |
| HUS00348792 | MiRBase | MIMAT0005900 | Homo_sapiens | mature_miRNA | hsa-miR-1248 | 1248 | ACCTTCTTGTATAAGCACTGTGCTAAA | 79 | 61 | 13 | 5 | 28,06117111 | -1,418638957 | 0,463144396 | -3,063059747 | 2,19E-03 | 3,87E-03 |
| HUS00117855 | MiRBase | MIMAT0004494 | Homo_sapiens | mature_miRNA | hsa-miR-21-3p | 21 | CAACACCAGTCGATGGGCTGT | 9270 | 8041 | 763 | 1368 | 3521,688046 | -1,395927723 | 0,456415937 | -3,058455261 | 2,22E-03 | 3,92E-03 |
| HUS00077824 | MiRBase | MIMAT0000425 | Homo_sapiens | mature_miRNA | hsa-miR-130a-3p | 130 | CAGTGCAATGTTAAAAGGGCAT | 232 | 239 | 338 | 117 | 253,4012387 | 1,333617437 | 0,457446232 | 2,915353422 | 3,55E-03 | 6,21E-03 |
| HUS00326588 | MiRBase | MIMAT0004945 | Homo_sapiens | mature_miRNA | hsa-miR-744-5p | 744 | TGCGGGGCTAGGGCTAACAGCA | 1257 | 913 | 722 | 860 | 1006,821516 | 1,065795929 | 0,366361933 | 2,909133925 | 3,62E-03 | 6,31E-03 |
| HUS00124307 | MiRBase | MIMAT0000072 | Homo_sapiens | mature_miRNA | hsa-miR-18a-5p | 17 | TAAGGTGCATCTAGTGCAGATAG | 512 | 617 | 125 | 76 | 252,7384293 | -0,995834103 | 0,346885559 | -2,870785699 | 4,09E-03 | 7,08E-03 |
| HUS00305139 | MiRBase | MIMAT0003338 | Homo_sapiens | mature_miRNA | hsa-miR-660-5p | 188 | TACCCATTGCATATCGGAGTTG | 1036 | 573 | 162 | 87 | 339,735651 | -1,171149306 | 0,410355973 | -2,853983816 | 4,32E-03 | 7,44E-03 |
| HUS00196430 | MiRBase | MIMAT0003393 | Homo_sapiens | mature_miRNA | hsa-miR-425-5p | 425 | AATGACACGATCACTCCCGTTGA | 2725 | 2413 | 2188 | 1253 | 2188,830921 | 0,882431546 | 0,309593884 | 2,850287401 | 4,37E-03 | 7,49E-03 |
| HUS00157605 | MiRBase | MIMAT0000688 | Homo_sapiens | mature_miRNA | hsa-miR-301a-3p | 130 | CAGTGCAATAGTATTGTCAAAGC | 133 | 160 | 32 | 12 | 61,70008811 | -1,238281748 | 0,440603359 | -2,81042285 | 4,95E-03 | 8,46E-03 |
| HUS00054339 | MiRBase | MIMAT0004958 | Homo_sapiens | mature_miRNA | hsa-miR-301b-3p | 130 | CAGTGCAATGATATTGTCAAAGC | 184 | 235 | 43 | 28 | 92,50942906 | -1,054776647 | 0,379617705 | -2,778523325 | 5,46E-03 | 9,27E-03 |
| HUS00363631 | MiRBase | MIMAT0000758 | Homo_sapiens | mature_miRNA | hsa-miR-135b-5p | 135 | TATGGCTTTTCATTCCTATGTGA | 823 | 928 | 202 | 85 | 378,785862 | -1,130936374 | 0,408812454 | -2,76639413 | 5,67E-03 | 9,58E-03 |
| HUS00074818 | MiRBase | MIMAT0004956 | Homo_sapiens | mature_miRNA | hsa-miR-374b-3p | 374 | CTTAGCAGGTTGTATTATCATT | 195 | 186 | 41 | 30 | 86,64673884 | -0,909819514 | 0,339143726 | -2,682695992 | 7,30E-03 | 1,23E-02 |
| HUS00154599 | MiRBase | MIMAT0004673 | Homo_sapiens | mature_miRNA | hsa-miR-29c-5p | 29 | TGACCGATTTCTCCTGGTGTTC | 68 | 67 | 8 | 12 | 28,93177686 | -1,194524456 | 0,448332172 | -2,664373717 | 7,71E-03 | 1,29E-02 |
| HUS00234961 | MiRBase | MIMAT0004487 | Homo_sapiens | mature_miRNA | hsa-let-7f-2-3p | let7 | CTATACAGTCTACTGTCTTTCC | 204 | 140 | 19 | 31 | 72,99276584 | -1,187085642 | 0,446319294 | -2,659722891 | 7,82E-03 | 1,31E-02 |
| HUS00135032 | MiRBase | MIMAT0000680 | Homo_sapiens | mature_miRNA | hsa-miR-106b-5p | 17 | TAAAGTGCTGACAGTGCAGAT | 2245 | 1887 | 472 | 152 | 864,5921731 | -1,245596788 | 0,473659695 | -2,629729322 | 8,55E-03 | 1,42E-02 |
| HUS00223555 | MiRBase | MIMAT0004688 | Homo_sapiens | mature_miRNA | hsa-miR-374a-3p | 374 | CTTATCAGATTGTATTGTAATT | 784 | 904 | 201 | 133 | 392,2528002 | -0,842146493 | 0,321142968 | -2,622341374 | 8,73E-03 | 1,45E-02 |
| HUS00137971 | MiRBase | MIMAT0000260 | Homo_sapiens | mature_miRNA | hsa-miR-182-3p | 182 | TGGTTCTAGACTTGCCAACTA | 142 | 84 | 21 | 15 | 48,44996086 | -1,106377149 | 0,426714276 | -2,592782129 | 9,52E-03 | 1,57E-02 |
| HUS00032905 | MiRBase | MIMAT0004692 | Homo_sapiens | mature_miRNA | hsa-miR-340-5p | 340 | TTATAAAGCAATGAGACTGATT | 7260 | 6632 | 1317 | 1413 | 3244,523162 | -0,804417529 | 0,312522427 | -2,573951366 | 1,01E-02 | 1,65E-02 |
| HUS00209158 | MiRBase | MIMAT0004504 | Homo_sapiens | mature_miRNA | hsa-miR-31-3p | 31 | TGCTATGCCAACATATTGCCAT | 72 | 70 | 17 | 6 | 30,4482879 | -1,112453582 | 0,445091403 | -2,499382318 | 1,24E-02 | 2,03E-02 |
| HUS00328720 | MiRBase | MIMAT0004551 | Homo_sapiens | mature_miRNA | hsa-miR-30d-3p | 30 | CTTTCAGTCAGATGTTTGCTGC | 457 | 357 | 87 | 76 | 190,0923872 | -0,792751963 | 0,318284868 | -2,490699505 | 1,27E-02 | 2,07E-02 |
| HUS00172157 | MiRBase | MIMAT0000757 | Homo_sapiens | mature_miRNA | hsa-miR-151a-3p | 28 | CTAGACTGAAGCTCCTTGAGG | 33938 | 37360 | 20960 | 21165 | 28725,38694 | 0,747917658 | 0,301994124 | 2,476596725 | 1,33E-02 | 2,15E-02 |
| HUS00177237 | MiRBase | MIMAT0003886 | Homo_sapiens | mature_miRNA | hsa-miR-769-5p | 769 | TGAGACCTCTGGGTTCTGAGCT | 1605 | 1963 | 1038 | 1494 | 1644,369924 | 1,020643073 | 0,417223579 | 2,446273713 | 1,44E-02 | 2,32E-02 |
| HUS00063789 | MiRBase | MIMAT0004549 | Homo_sapiens | mature_miRNA | hsa-miR-148a-5p | 148 | AAAGTTCTGAGACACTCCGACT | 167 | 312 | 19 | 41 | 99,3793909 | -1,366153511 | 0,56221624 | -2,429943166 | 1,51E-02 | 2,42E-02 |
| HUS00024184 | MiRBase | MIMAT0000420 | Homo_sapiens | mature_miRNA | hsa-miR-30b-5p | 30 | TGTAAACATCCTACACTCAGCT | 4759 | 4650 | 1227 | 466 | 2088,354328 | -1,004680357 | 0,424041088 | -2,369299543 | 1,78E-02 | 2,85E-02 |
| HUS00087565 | MiRBase | MIMAT0000750 | Homo_sapiens | mature_miRNA | hsa-miR-340-3p | 340 | TCCGTCTCAGTTACTTTATAGC | 109 | 81 | 60 | 59 | 79,10440772 | 0,829655797 | 0,352930636 | 2,350761626 | 1,87E-02 | 2,97E-02 |
| HUS00167922 | MiRBase | MIMAT0000772 | Homo_sapiens | mature_miRNA | hsa-miR-345-5p | 345 | GCTGACTCCTAGTCCAGGGCTC | 558 | 397 | 429 | 214 | 405,3917745 | 0,879816647 | 0,374279935 | 2,350691459 | 1,87E-02 | 2,97E-02 |
| HUS00237964 | MiRBase | MIMAT0000692 | Homo_sapiens | mature_miRNA | hsa-miR-30e-5p | 30 | TGTAAACATCCTTGACTGGAAG | 5160 | 3231 | 1046 | 426 | 1837,004816 | -1,019455852 | 0,434496886 | -2,346290354 | 1,90E-02 | 3,00E-02 |
| HUS00123005 | MiRBase | MIMAT0000764 | Homo_sapiens | mature_miRNA | hsa-miR-339-5p | 339 | TCCCTGTCCTCCAGGAGCTCACG | 328 | 176 | 221 | 130 | 219,6959013 | 0,945385333 | 0,403511915 | 2,342893227 | 1,91E-02 | 3,02E-02 |
| HUS00145874 | MiRBase | MIMAT0000753 | Homo_sapiens | mature_miRNA | hsa-miR-342-3p | 342 | TCTCACACAGAAATCGCACCCGT | 178 | 514 | 502 | 205 | 392,484212 | 1,358377555 | 0,584738486 | 2,323051393 | 2,02E-02 | 3,16E-02 |
| HUS00192933 | MiRBase | MIMAT0002871 | Homo_sapiens | mature_miRNA | hsa-miR-500a-3p | 500 | ATGCACCTGGGCAAGGATTCTG | 397 | 296 | 256 | 159 | 275,3522978 | 0,736071447 | 0,324333945 | 2,269486311 | 2,32E-02 | 3,61E-02 |
| HUS00120552 | MiRBase | MIMAT0000087 | Homo_sapiens | mature_miRNA | hsa-miR-30a-5p | 30 | TGTAAACATCCTCGACTGGAAG | 7433 | 7061 | 1539 | 1586 | 3502,71118 | -0,678216465 | 0,303468113 | -2,234885432 | 2,54E-02 | 3,93E-02 |
| HUS00285455 | MiRBase | MIMAT0000095 | Homo_sapiens | mature_miRNA | hsa-miR-96-5p | 96 | TTTGGCACTAGCACATTTTTGCT | 12870 | 9494 | 2954 | 1168 | 4985,925787 | -0,958714361 | 0,430684 | -2,226027345 | 2,60E-02 | 4,01E-02 |
| HUS00015877 | MiRBase | MIMAT0000254 | Homo_sapiens | mature_miRNA | hsa-miR-10b-5p | 10 | TACCCTGTAGAACCGAATTTGTG | 7510 | 12281 | 6167 | 8203 | 9252,679949 | 1,027780097 | 0,476441446 | 2,157201282 | 3,10E-02 | 4,71E-02 |
| HUS00358483 | MiRBase | MIMAT0004928 | Homo_sapiens | mature_miRNA | hsa-miR-147b | 147 | GTGTGCGGAAATGCTTCTGCTA | 57 | 55 | 5 | 13 | 24,84959074 | -1,054341515 | 0,491095111 | -2,146919185 | 3,18E-02 | 4,82E-02 |
| HUS00090560 | MiRBase | MIMAT0004693 | Homo_sapiens | mature_miRNA | hsa-miR-330-5p | 330 | TCTCTGGGCCTGTGTCTTAGGC | 375 | 290 | 338 | 129 | 287,5310843 | 0,908970763 | 0,433048342 | 2,099005293 | 3,58E-02 | 5,39E-02 |
| HUS00166636 | MiRBase | MIMAT0000081 | Homo_sapiens | mature_miRNA | hsa-miR-25-3p | 25 | CATTGCACTTGTCTCGGTCTGA | 9663 | 9468 | 7361 | 4129 | 7603,405856 | 0,719788222 | 0,343301536 | 2,096664727 | 3,60E-02 | 5,40E-02 |
| HUS00059375 | MiRBase | MIMAT0000243 | Homo_sapiens | mature_miRNA | hsa-miR-148a-3p | 148 | TCAGTGCACTACAGAACTTTGT | 31945 | 51984 | 3642 | 8250 | 18008,06423 | -1,175893226 | 0,56924207 | -2,065717362 | 3,89E-02 | 5,81E-02 |
| HUS00165368 | MiRBase | MIMAT0000101 | Homo_sapiens | mature_miRNA | hsa-miR-103a-3p | 103 | AGCAGCATTGTACAGGGCTATGA | 76511 | 90650 | 72186 | 34209 | 68589,19578 | 0,778374678 | 0,377877884 | 2,059857724 | 3,94E-02 | 5,87E-02 |
| HUS00216180 | MiRBase | MIMAT0000080 | Homo_sapiens | mature_miRNA | hsa-miR-24-3p | 24 | TGGCTCAGTTCAGCAGGAACAG | 81273 | 82807 | 95164 | 29599 | 74428,87498 | 0,986449695 | 0,480178231 | 2,054340724 | 3,99E-02 | 5,93E-02 |
| HUS00006058 | MiRBase | MIMAT0000416 | Homo_sapiens | mature_miRNA | hsa-miR-1-3p | 1 | TGGAATGTAAAGAAGTATGTAT | 1328 | 1311 | 826 | 581 | 983,4690531 | 0,574776666 | 0,280643208 | 2,048069042 | 4,06E-02 | 5,99E-02 |
| HUS00207346 | MiRBase | MIMAT0010195 | Homo_sapiens | mature_miRNA | hsa-let-7a-2-3p | let7 | CTGTACAGCCTCCTAGCTTTCC | 64 | 73 | 12 | 14 | 31,76064456 | -0,860780987 | 0,426956839 | -2,016084316 | 4,38E-02 | 6,44E-02 |
| HUS00122515 | MiRBase | MIMAT0000759 | Homo_sapiens | mature_miRNA | hsa-miR-148b-3p | 148 | TCAGTGCATCACAGAACTTTGT | 23639 | 27673 | 6865 | 4537 | 12447,40253 | -0,67833317 | 0,342101572 | -1,982841428 | 4,74E-02 | 6,93E-02 |
| HUS00196798 | MiRBase | MIMAT0005876 | Homo_sapiens | mature_miRNA | hsa-miR-1285-3p | 1285 | TCTGGGCAACAAAGTGAGACCT | 87 | 67 | 19 | 12 | 35,76095156 | -0,792708247 | 0,403776878 | -1,963233384 | 4,96E-02 | 7,21E-02 |
| HUS00004298 | MiRBase | MIMAT0004701 | Homo_sapiens | mature_miRNA | hsa-miR-338-5p | 338 | AACAATATCCTGGTGCTGAGTG | 167 | 87 | 75 | 82 | 105,0187074 | 0,824133987 | 0,42374384 | 1,94488724 | 5,18E-02 | 7,50E-02 |
| HUS00194110 | MiRBase | MIMAT0000278 | Homo_sapiens | mature_miRNA | hsa-miR-221-3p | 221 | AGCTACATTGTCTGCTGGGTTTC | 69280 | 51294 | 63580 | 20997 | 51822,42908 | 0,89167835 | 0,460566231 | 1,936048043 | 5,29E-02 | 7,63E-02 |
| HUS00009768 | MiRBase | MIMAT0018206 | Homo_sapiens | mature_miRNA | hsa-miR-3929 | 3929 | GAGGCTGATGTGAGTAGACCACT | 65 | 69 | 33 | 49 | 56,02311243 | 0,806790179 | 0,422535945 | 1,909400108 | 5,62E-02 | 8,09E-02 |
| HUS00001045 | MiRBase | MIMAT0000267 | Homo_sapiens | mature_miRNA | hsa-miR-210-3p | 210 | CTGTGCGTGTGACAGCGGCTGA | 1046 | 986 | 1075 | 352 | 875,4056875 | 0,884554327 | 0,470681146 | 1,879306903 | 6,02E-02 | 8,64E-02 |
| HUS00051340 | MiRBase | MIMAT0000064 | Homo_sapiens | mature_miRNA | hsa-let-7c-5p | let7 | TGAGGTAGTAGGTTGTATGGTT | 17108 | 11151 | 7285 | 9500 | 11488,82321 | 0,781608098 | 0,430648893 | 1,814954389 | 6,95E-02 | 9,83E-02 |
| HUS00002012 | MiRBase | MIMAT0002870 | Homo_sapiens | mature_miRNA | hsa-miR-499a-5p | 499 | TTAAGACTTGCAGTGATGTTT | 885 | 474 | 163 | 127 | 322,4249938 | -0,692958108 | 0,388185698 | -1,785120142 | 7,42E-02 | 1,04E-01 |
| HUS00151922 | MiRBase | MIMAT0003150 | Homo_sapiens | mature_miRNA | hsa-miR-455-5p | 455 | TATGTGCCTTTGGACTACATCG | 705 | 478 | 150 | 125 | 291,5551454 | -0,578659632 | 0,330162164 | -1,752652775 | 7,97E-02 | 1,12E-01 |
| HUS00159377 | MiRBase | MIMAT0001545 | Homo_sapiens | mature_miRNA | hsa-miR-450a-5p | 450 | TTTTGCGATGTGTTCCTAATAT | 3644 | 3394 | 910 | 839 | 1794,534464 | -0,48695819 | 0,28038705 | -1,736735664 | 8,24E-02 | 1,15E-01 |
| HUS00042578 | MiRBase | MIMAT0002883 | Homo_sapiens | mature_miRNA | hsa-miR-514a-3p | 506 | ATTGACACTTCTGTGAGTAGA | 63 | 113 | 22 | 14 | 41,60576494 | -0,786998808 | 0,47431741 | -1,659223951 | 9,71E-02 | 1,34E-01 |
| HUS00110371 | MiRBase | MIMAT0004909 | Homo_sapiens | mature_miRNA | hsa-miR-450b-5p | 450 | TTTTGCAATATGTTCCTGAATA | 1404 | 2011 | 351 | 400 | 838,3144732 | -0,647309573 | 0,398740632 | -1,623385031 | 1,05E-01 | 1,44E-01 |
| HUS00094161 | MiRBase | MIMAT0005878 | Homo_sapiens | mature_miRNA | hsa-miR-1287-5p | 1287 | TGCTGGATCAGTGGTTCGAGTC | 446 | 336 | 246 | 155 | 282,9041919 | 0,517328708 | 0,320132159 | 1,615984818 | 1,06E-01 | 1,46E-01 |
| HUS00060833 | MiRBase | MIMAT0000682 | Homo_sapiens | mature_miRNA | hsa-miR-200a-3p | 8 | TAACACTGTCTGGTAACGATGT | 131622 | 82892 | 23260 | 25828 | 52865,62842 | -0,570041899 | 0,359218984 | -1,586892466 | 1,13E-01 | 1,53E-01 |
| HUS00334209 | MiRBase | MIMAT0000705 | Homo_sapiens | mature_miRNA | hsa-miR-362-5p | 362 | AATCCTTGGAACCTAGGTGTGAGT | 394 | 398 | 268 | 144 | 288,1028817 | 0,514855188 | 0,341029405 | 1,509709076 | 1,31E-01 | 1,77E-01 |
| HUS00244129 | MiRBase | MIMAT0000094 | Homo_sapiens | mature_miRNA | hsa-miR-95-3p | 95 | TTCAACGGGTATTTATTGAGCA | 2279 | 2824 | 1210 | 1412 | 1899,693122 | 0,554029265 | 0,36693873 | 1,509868596 | 1,31E-01 | 1,77E-01 |
| HUS00022334 | MiRBase | MIMAT0002874 | Homo_sapiens | mature_miRNA | hsa-miR-503-5p | 503 | TAGCAGCGGGAACAGTTCTGCAG | 541 | 782 | 328 | 375 | 502,8450032 | 0,591589418 | 0,400750376 | 1,476204276 | 1,40E-01 | 1,87E-01 |
| HUS00074709 | MiRBase | MIMAT0000417 | Homo_sapiens | mature_miRNA | hsa-miR-15b-5p | 15 | TAGCAGCACATCATGGTTTACA | 359 | 427 | 387 | 110 | 315,8214075 | 0,718344057 | 0,512747937 | 1,400969181 | 1,61E-01 | 2,15E-01 |
| HUS00106205 | MiRBase | MIMAT0004702 | Homo_sapiens | mature_miRNA | hsa-miR-339-3p | 339 | TGAGCGCCTCGACGACAGAGCCG | 630 | 342 | 267 | 225 | 351,6918502 | 0,528381366 | 0,381363565 | 1,38550563 | 1,66E-01 | 2,19E-01 |
| HUS00340664 | MiRBase | MIMAT0000252 | Homo_sapiens | mature_miRNA | hsa-miR-7-5p | 7 | TGGAAGACTAGTGATTTTGTTGT | 814 | 1019 | 406 | 536 | 685,5990465 | 0,561050821 | 0,405003897 | 1,385297338 | 1,66E-01 | 2,19E-01 |
| HUS00299348 | MiRBase | MIMAT0000455 | Homo_sapiens | mature_miRNA | hsa-miR-185-5p | 185 | TGGAGAGAAAGGCAGTTCCTGA | 4475 | 2914 | 1083 | 818 | 1892,777435 | -0,44182984 | 0,320815601 | -1,377208085 | 1,68E-01 | 2,21E-01 |
| HUS00108901 | MiRBase | MIMAT0000456 | Homo_sapiens | mature_miRNA | hsa-miR-186-5p | 186 | CAAAGAATTCTCCTTTTGGGCT | 13555 | 6924 | 8966 | 3381 | 8001,804588 | 0,695758148 | 0,508514916 | 1,368215809 | 1,71E-01 | 2,24E-01 |
| HUS00115905 | MiRBase | MIMAT0000438 | Homo_sapiens | mature_miRNA | hsa-miR-152-3p | 148 | TCAGTGCATGACAGAACTTGG | 1437 | 1402 | 1223 | 407 | 1078,020342 | 0,605317102 | 0,465351028 | 1,300775255 | 1,93E-01 | 2,51E-01 |
| HUS00329822 | MiRBase | MIMAT0000441 | Homo_sapiens | mature_miRNA | hsa-miR-9-5p | 9 | TCTTTGGTTATCTAGCTGTATGA | 4240 | 5190 | 3099 | 1642 | 3366,970224 | 0,455768874 | 0,357629178 | 1,274417475 | 2,03E-01 | 2,62E-01 |
| HUS00176710 | MiRBase | MIMAT0000104 | Homo_sapiens | mature_miRNA | hsa-miR-107 | 103 | AGCAGCATTGTACAGGGCTATCA | 4597 | 3486 | 3709 | 1048 | 3094,043786 | 0,631421087 | 0,505855526 | 1,248224157 | 2,12E-01 | 2,72E-01 |
| HUS00021488 | MiRBase | MIMAT0000083 | Homo_sapiens | mature_miRNA | hsa-miR-26b-5p | 26 | TTCAAGTAATTCAGGATAGGT | 27904 | 17312 | 16079 | 7600 | 16367,2735 | 0,520667359 | 0,42062722 | 1,237835628 | 2,16E-01 | 2,76E-01 |
| HUS00101701 | MiRBase | MIMAT0002888 | Homo_sapiens | mature_miRNA | hsa-miR-532-5p | 188 | CATGCCTTGAGTGTAGGACCGT | 19866 | 22774 | 8464 | 13133 | 15889,18063 | 0,554114708 | 0,449109014 | 1,233808919 | 2,17E-01 | 2,78E-01 |
| HUS00221643 | MiRBase | MIMAT0000264 | Homo_sapiens | mature_miRNA | hsa-miR-203a-3p | 203 | GTGAAATGTTTAGGACCACTAG | 9123 | 16774 | 3007 | 3180 | 6577,815253 | -0,539123924 | 0,457255326 | -1,179043508 | 2,38E-01 | 3,03E-01 |
| HUS00299630 | MiRBase | MIMAT0004559 | Homo_sapiens | mature_miRNA | hsa-miR-181c-3p | 181 | AACCATCGACCGTTGAGTGGAC | 355 | 371 | 152 | 187 | 255,6111482 | 0,42622937 | 0,366598249 | 1,162660683 | 2,45E-01 | 3,10E-01 |
| HUS00307885 | MiRBase | MIMAT0002876 | Homo_sapiens | mature_miRNA | hsa-miR-505-3p | 505 | CGTCAACACTTGCTGGTTTCCT | 77 | 101 | 34 | 10 | 44,29543992 | -0,555090556 | 0,486603142 | -1,140745934 | 2,54E-01 | 3,19E-01 |
| HUS00068672 | MiRBase | MIMAT0000445 | Homo_sapiens | mature_miRNA | hsa-miR-126-3p | 126 | TCGTACCGTGAGTAATAATGCG | 3319 | 2831 | 1123 | 553 | 1602,231781 | -0,403205333 | 0,354156924 | -1,138493437 | 2,55E-01 | 3,20E-01 |
| HUS00188312 | MiRBase | MIMAT0004796 | Homo_sapiens | mature_miRNA | hsa-miR-576-3p | 576 | AAGATGTGGAAAAATTGGAATC | 195 | 151 | 45 | 46 | 90,39910526 | -0,395703281 | 0,352712312 | -1,121886783 | 2,62E-01 | 3,26E-01 |
| HUS00149815 | MiRBase | MIMAT0018186 | Homo_sapiens | mature_miRNA | hsa-miR-3912-3p | 3912 | TAACGCATAATATGGACATGT | 112 | 150 | 47 | 21 | 67,10500853 | -0,475300743 | 0,427620273 | -1,111501892 | 2,66E-01 | 3,30E-01 |
| HUS00149512 | MiRBase | MIMAT0004481 | Homo_sapiens | mature_miRNA | hsa-let-7a-3p | let7 | CTATACAATCTACTGTCTTTC | 388 | 77 | 140 | 125 | 180,0471047 | 0,691033004 | 0,628600563 | 1,099319734 | 2,72E-01 | 3,34E-01 |
| HUS00261293 | MiRBase | MIMAT0002819 | Homo_sapiens | mature_miRNA | hsa-miR-193b-3p | 193 | AACTGGCCCTCAAAGTCCCGCT | 65 | 84 | 27 | 47 | 55,15167636 | 0,51700771 | 0,469649178 | 1,100838101 | 2,71E-01 | 3,34E-01 |
| HUS00055793 | MiRBase | MIMAT0000089 | Homo_sapiens | mature_miRNA | hsa-miR-31-5p | 31 | AGGCAAGATGCTGGCATAGCT | 2995 | 2400 | 2105 | 735 | 1944,414512 | 0,495103649 | 0,450184032 | 1,099780565 | 2,71E-01 | 3,34E-01 |
| HUS00203520 | MiRBase | MIMAT0000707 | Homo_sapiens | mature_miRNA | hsa-miR-363-3p | 363 | AATTGCACGGTATCCATCTGTA | 108 | 95 | 32 | 23 | 53,29790659 | -0,377677159 | 0,357199425 | -1,057328575 | 2,90E-01 | 3,54E-01 |
| HUS00114474 | MiRBase | MIMAT0000070 | Homo_sapiens | mature_miRNA | hsa-miR-17-5p | 17 | CAAAGTGCTTACAGTGCAGGTAG | 7079 | 6393 | 6091 | 1524 | 5023,313464 | 0,55877367 | 0,541252569 | 1,032371396 | 3,02E-01 | 3,67E-01 |
| HUS00011339 | MiRBase | MIMAT0000765 | Homo_sapiens | mature_miRNA | hsa-miR-335-5p | 335 | TCAAGAGCAATAACGAAAAATGT | 1247 | 719 | 936 | 207 | 740,4055673 | 0,598277034 | 0,58901557 | 1,015723631 | 3,10E-01 | 3,75E-01 |
| HUS00316822 | MiRBase | MIMAT0018925 | Homo_sapiens | mature_miRNA | hsa-miR-1268b | 1268 | CGGGCGTGGTGGTGGGGGTG | 67 | 71 | 31 | 32 | 47,67350573 | 0,372442643 | 0,371367112 | 1,002896138 | 3,16E-01 | 3,81E-01 |
| HUS00203479 | MiRBase | MIMAT0000732 | Homo_sapiens | mature_miRNA | hsa-miR-378a-3p | 378 | ACTGGACTTGGAGTCAGAAGGC | 22890 | 24322 | 9230 | 12314 | 16453,83134 | 0,401539187 | 0,400768291 | 1,001923547 | 3,16E-01 | 3,81E-01 |
| HUS00221936 | MiRBase | MIMAT0005922 | Homo_sapiens | mature_miRNA | hsa-miR-1268a | 1268 | CGGGCGTGGTGGTGGGGG | 66 | 70 | 31 | 31 | 46,89269982 | 0,368670661 | 0,369756133 | 0,997064357 | 3,19E-01 | 3,83E-01 |
| HUS00127823 | MiRBase | MIMAT0000069 | Homo_sapiens | mature_miRNA | hsa-miR-16-5p | 15 | TAGCAGCACGTAAATATTGGCG | 3137 | 2932 | 1213 | 414 | 1558,73411 | -0,450975446 | 0,454439599 | -0,992377088 | 3,21E-01 | 3,85E-01 |
| HUS00363289 | MiRBase | MIMAT0000063 | Homo_sapiens | mature_miRNA | hsa-let-7b-5p | let7 | TGAGGTAGTAGGTTGTGTGGTT | 57716 | 73599 | 27438 | 30009 | 44461,16099 | 0,321790157 | 0,34523558 | 0,932088624 | 3,51E-01 | 4,20E-01 |
| HUS00196288 | MiRBase | MIMAT0000066 | Homo_sapiens | mature_miRNA | hsa-let-7e-5p | let7 | TGAGGTAGGAGGTTGTATAGTT | 8062 | 7467 | 3956 | 2690 | 5093,509836 | 0,260604034 | 0,28149426 | 0,925788092 | 3,55E-01 | 4,22E-01 |
| HUS00338938 | MiRBase | MIMAT0000414 | Homo_sapiens | mature_miRNA | hsa-let-7g-5p | let7 | TGAGGTAGTAGTTTGTACAGTT | 342591 | 330935 | 108307 | 84406 | 181750,8747 | -0,30044869 | 0,331141228 | -0,907312846 | 3,64E-01 | 4,31E-01 |
| HUS00343657 | MiRBase | MIMAT0004589 | Homo_sapiens | mature_miRNA | hsa-miR-30b-3p | 30 | CTGGGAGGTGGATGTTTACTTC | 61 | 66 | 23 | 12 | 33,39692199 | -0,367828066 | 0,405581518 | -0,906915254 | 3,64E-01 | 4,31E-01 |
| HUS00190386 | MiRBase | MIMAT0000457 | Homo_sapiens | mature_miRNA | hsa-miR-188-5p | 188 | CATCCCTTGCATGGTGGAGGG | 65 | 53 | 27 | 4 | 29,57933685 | -0,4727092 | 0,554922756 | -0,851846847 | 3,94E-01 | 4,64E-01 |
| HUS00114989 | MiRBase | MIMAT0000077 | Homo_sapiens | mature_miRNA | hsa-miR-22-3p | 22 | AAGCTGCCAGTTGAAGAACTGT | 68170 | 54557 | 36993 | 17891 | 40716,0032 | 0,296505786 | 0,348195903 | 0,851548752 | 3,94E-01 | 4,64E-01 |
| HUS00209723 | MiRBase | MIMAT0005919 | Homo_sapiens | mature_miRNA | hsa-miR-548o-3p | 548 | CCAAAACTGCAGTTACTTTTGC | 110 | 140 | 58 | 51 | 83,84647125 | 0,295588578 | 0,3627313 | 0,814896807 | 4,15E-01 | 4,85E-01 |
| HUS00321713 | MiRBase | MIMAT0000727 | Homo_sapiens | mature_miRNA | hsa-miR-374a-5p | 374 | TTATAATACAACCTGATAAGTG | 1343 | 1112 | 545 | 116 | 625,0633025 | -0,459063568 | 0,575572321 | -0,79757756 | 4,25E-01 | 4,95E-01 |
| HUS00231444 | MiRBase | MIMAT0004697 | Homo_sapiens | mature_miRNA | hsa-miR-151a-5p | 28 | TCGAGGAGCTCACAGTCTAGT | 66367 | 10032 | 53567 | 7413 | 34667,34639 | 0,838980151 | 1,05480643 | 0,795387786 | 4,26E-01 | 4,96E-01 |
| HUS00224636 | MiRBase | MIMAT0030417 | Homo_sapiens | mature_miRNA | hsa-miR-6516-5p | 6516 | TTTGCAGTAACAGGTGTGAGCA | 92 | 65 | 33 | 34 | 52,0147436 | 0,288036742 | 0,378531485 | 0,760932058 | 4,47E-01 | 5,17E-01 |
| HUS00045284 | MiRBase | MIMAT0000460 | Homo_sapiens | mature_miRNA | hsa-miR-194-5p | 194 | TGTAACAGCAACTCCATGTGGA | 851 | 583 | 513 | 171 | 486,915391 | 0,358865899 | 0,47776017 | 0,751142356 | 4,53E-01 | 5,22E-01 |
| HUS00092501 | MiRBase | MIMAT0010214 | Homo_sapiens | mature_miRNA | hsa-miR-151b | 28 | TCGAGGAGCTCACAGTCT | 2296 | 251 | 2033 | 212 | 1230,269673 | 0,853495479 | 1,145932112 | 0,744804574 | 4,56E-01 | 5,25E-01 |
| HUS00273560 | MiRBase | MIMAT0016847 | Homo_sapiens | mature_miRNA | hsa-miR-378c | 378 | ACTGGACTTGGAGTCAGAAGAGTGG | 5675 | 4689 | 1636 | 1494 | 2873,945568 | -0,206137859 | 0,280255361 | -0,735535826 | 4,62E-01 | 5,28E-01 |
| HUS00234511 | MiRBase | MIMAT0018926 | Homo_sapiens | mature_miRNA | hsa-miR-378d | 378 | ACTGGACTTGGAGTCAGAAA | 1664 | 1193 | 360 | 442 | 771,3614718 | -0,277573857 | 0,377012164 | -0,736246422 | 4,62E-01 | 5,28E-01 |
| HUS00363967 | MiRBase | MIMAT0000082 | Homo_sapiens | mature_miRNA | hsa-miR-26a-5p | 26 | TTCAAGTAATCCAGGATAGGCT | 229978 | 128769 | 93641 | 61457 | 117591,0828 | 0,283994143 | 0,390765699 | 0,726763235 | 4,67E-01 | 5,33E-01 |
| HUS00175622 | MiRBase | MIMAT0004703 | Homo_sapiens | mature_miRNA | hsa-miR-335-3p | 335 | TTTTTCATTATTGCTCCTGACC | 536 | 770 | 205 | 346 | 440,1992945 | 0,295990571 | 0,480086885 | 0,616535422 | 5,38E-01 | 5,98E-01 |
| HUS00348518 | MiRBase | MIMAT0004505 | Homo_sapiens | mature_miRNA | hsa-miR-32-3p | 32 | CAATTTAGTGTGTGTGATATTT | 77 | 82 | 30 | 19 | 44,17214722 | -0,20569773 | 0,375397224 | -0,547946859 | 5,84E-01 | 6,46E-01 |
| HUS00002935 | MiRBase | MIMAT0000435 | Homo_sapiens | mature_miRNA | hsa-miR-143-3p | 143 | TGAGATGAAGCACTGTAGCTC | 79 | 62 | 37 | 21 | 44,9230918 | 0,20228234 | 0,376990698 | 0,536571171 | 5,92E-01 | 6,53E-01 |
| HUS00312965 | MiRBase | MIMAT0005882 | Homo_sapiens | mature_miRNA | hsa-miR-548k | 548 | AAAAGTACTTGCGGATTTTGCT | 252 | 167 | 71 | 94 | 133,9071822 | 0,201815733 | 0,409150103 | 0,493255976 | 6,22E-01 | 6,84E-01 |
| HUS00010900 | MiRBase | MIMAT0022696 | Homo_sapiens | mature_miRNA | hsa-miR-301a-5p | 130 | GCTCTGACTTTATTGCACTACT | 181 | 204 | 91 | 63 | 122,1028954 | 0,161708136 | 0,331429894 | 0,487910533 | 6,26E-01 | 6,86E-01 |
| HUS00009287 | MiRBase | MIMAT0004550 | Homo_sapiens | mature_miRNA | hsa-miR-30c-2-3p | 30 | CTGGGAGAAGGCTGTTTACTCT | 112 | 111 | 31 | 38 | 63,22333693 | -0,162928248 | 0,379082082 | -0,429796754 | 6,67E-01 | 7,28E-01 |
| HUS00075510 | MiRBase | MIMAT0004568 | Homo_sapiens | mature_miRNA | hsa-miR-221-5p | 221 | ACCTGGCATACAATGTAGATTT | 1559 | 1259 | 681 | 417 | 874,8846887 | 0,122514224 | 0,310239581 | 0,394901977 | 6,93E-01 | 7,55E-01 |
| HUS00248747 | MiRBase | MIMAT0000084 | Homo_sapiens | mature_miRNA | hsa-miR-27a-3p | 27 | TTCACAGTGGCTAAGTTCCGC | 95118 | 90196 | 47040 | 25781 | 57686,98259 | 0,119144851 | 0,313513912 | 0,380030508 | 7,04E-01 | 7,63E-01 |
| HUS00240268 | MiRBase | MIMAT0000419 | Homo_sapiens | mature_miRNA | hsa-miR-27b-3p | 27 | TTCACAGTGGCTAAGTTCTGC | 34566 | 36476 | 13512 | 13402 | 22143,51367 | 0,116145484 | 0,304921802 | 0,380902523 | 7,03E-01 | 7,63E-01 |
| HUS00261584 | MiRBase | MIMAT0000431 | Homo_sapiens | mature_miRNA | hsa-miR-140-5p | 140 | CAGTGGTTTTACCCTATGGTAG | 735 | 484 | 296 | 99 | 338,9227524 | -0,174734862 | 0,477880623 | -0,365645421 | 7,15E-01 | 7,68E-01 |
| HUS00130828 | MiRBase | MIMAT0003885 | Homo_sapiens | mature_miRNA | hsa-miR-454-3p | 454 | TAGTGCAATATTGCTTATAGGGT | 319 | 421 | 188 | 55 | 208,115137 | -0,183560306 | 0,512138562 | -0,358419225 | 7,20E-01 | 7,71E-01 |
| HUS00235996 | MiRBase | MIMAT0004955 | Homo_sapiens | mature_miRNA | hsa-miR-374b-5p | 374 | ATATAATACAACCTGCTAAGTG | 1774 | 2429 | 1288 | 471 | 1341,525741 | 0,161891153 | 0,466246234 | 0,347222437 | 7,28E-01 | 7,77E-01 |
| HUS00295291 | MiRBase | MIMAT0000258 | Homo_sapiens | mature_miRNA | hsa-miR-181c-5p | 181 | AACATTCAACCTGTCGGTGAGT | 827 | 406 | 260 | 137 | 344,7048112 | -0,142773949 | 0,437822694 | -0,326099927 | 7,44E-01 | 7,92E-01 |
| HUS00000020 | MiRBase | MIMAT0002809 | Homo_sapiens | mature_miRNA | hsa-miR-146b-5p | 146 | TGAGAACTGAATTCCATAGGCT | 1309 | 794 | 338 | 330 | 596,2455688 | -0,118263298 | 0,365845851 | -0,323259914 | 7,46E-01 | 7,93E-01 |
| HUS00359261 | MiRBase | MIMAT0018927 | Homo_sapiens | mature_miRNA | hsa-miR-378e | 378 | ACTGGACTTGGAGTCAGGA | 148 | 66 | 43 | 25 | 59,55995616 | -0,146888428 | 0,466689273 | -0,31474567 | 7,53E-01 | 7,96E-01 |
| HUS00011999 | MiRBase | MIMAT0004795 | Homo_sapiens | mature_miRNA | hsa-miR-574-5p | 574 | TGAGTGTGTGTGTGTGAGTGTGT | 326 | 516 | 95 | 162 | 240,695273 | -0,156190405 | 0,495628081 | -0,315136311 | 7,53E-01 | 7,96E-01 |
| HUS00297603 | MiRBase | MIMAT0000096 | Homo_sapiens | mature_miRNA | hsa-miR-98-5p | let7 | TGAGGTAGTAAGTTGTATTGTT | 14696 | 14802 | 6260 | 3633 | 8501,586068 | -0,102399675 | 0,340888197 | -0,300390792 | 7,64E-01 | 8,05E-01 |
| HUS00305224 | MiRBase | MIMAT0000222 | Homo_sapiens | mature_miRNA | hsa-miR-192-5p | 192 | CTGACCTATGAATTGACAGCC | 1850 | 1272 | 472 | 680 | 966,925663 | 0,122115375 | 0,420780095 | 0,290211863 | 7,72E-01 | 8,12E-01 |
| HUS00115329 | MiRBase | MIMAT0001620 | Homo_sapiens | mature_miRNA | hsa-miR-200a-5p | 8 | CATCTTACCGGACAGTGCTGGA | 3246 | 4585 | 964 | 1491 | 2258,272755 | -0,12148976 | 0,451875283 | -0,268856839 | 7,88E-01 | 8,27E-01 |
| HUS00014488 | MiRBase | MIMAT0001080 | Homo_sapiens | mature_miRNA | hsa-miR-196b-5p | 196 | TAGGTAGTTTCCTGTTGTTGGG | 2812 | 2567 | 1371 | 466 | 1539,711601 | -0,113484535 | 0,455732606 | -0,249015615 | 8,03E-01 | 8,41E-01 |
| HUS00268487 | MiRBase | MIMAT0000093 | Homo_sapiens | mature_miRNA | hsa-miR-93-5p | 17 | CAAAGTGCTGTTCGTGCAGGTAG | 8698 | 12736 | 5428 | 1926 | 6189,361419 | -0,118390054 | 0,495499623 | -0,238930665 | 8,11E-01 | 8,45E-01 |
| HUS00090028 | MiRBase | MIMAT0022727 | Homo_sapiens | mature_miRNA | hsa-miR-1307-5p | 1307 | TCGACCGGACCTCGACCGGCT | 748 | 428 | 274 | 168 | 357,0689392 | 0,081694352 | 0,381072859 | 0,214379876 | 8,30E-01 | 8,62E-01 |
| HUS00121119 | MiRBase | MIMAT0004586 | Homo_sapiens | mature_miRNA | hsa-miR-15b-3p | 15 | CGAATCATTATTTGCTGCTCTA | 661 | 605 | 326 | 157 | 386,4610557 | 0,069803494 | 0,363878514 | 0,191831864 | 8,48E-01 | 8,78E-01 |
| HUS00275841 | MiRBase | MIMAT0000318 | Homo_sapiens | mature_miRNA | hsa-miR-200b-3p | 8 | TAATACTGCCTGGTAATGATGA | 89596 | 60493 | 36808 | 19451 | 45379,57238 | 0,059601957 | 0,349083438 | 0,170738427 | 8,64E-01 | 8,93E-01 |
| HUS00189290 | MiRBase | MIMAT0000065 | Homo_sapiens | mature_miRNA | hsa-let-7d-5p | let7 | AGAGGTAGTAGGTTGCATAGTT | 18732 | 15418 | 6852 | 5530 | 10309,57555 | 0,043316595 | 0,306617299 | 0,141272508 | 8,88E-01 | 9,15E-01 |
| HUS00328928 | MiRBase | MIMAT0000266 | Homo_sapiens | mature_miRNA | hsa-miR-205-5p | 17 | TCCTTCATTCCACCGGAGTCTG | 2108 | 2097 | 922 | 550 | 1238,322761 | -0,038549919 | 0,308213723 | -0,125075284 | 9,00E-01 | 9,25E-01 |
| HUS00322329 | MiRBase | MIMAT0004501 | Homo_sapiens | mature_miRNA | hsa-miR-27a-5p | 27 | AGGGCTTAGCTGCTTGTGAGCA | 1484 | 1977 | 469 | 681 | 1024,118024 | -0,044421464 | 0,434684896 | -0,102192334 | 9,19E-01 | 9,35E-01 |
| HUS00025602 | MiRBase | MIMAT0005794 | Homo_sapiens | mature_miRNA | hsa-miR-1296-5p | 1296 | TTAGGGCCCTGGCTCCATCTCC | 429 | 391 | 141 | 149 | 246,8362984 | 0,025306353 | 0,325277023 | 0,077799387 | 9,38E-01 | 9,49E-01 |
| HUS00152146 | MiRBase | MIMAT0017997 | Homo_sapiens | mature_miRNA | hsa-miR-3617-5p | 3617 | AAAGACATAGTTGCAAGATGGG | 93 | 76 | 43 | 19 | 50,38662356 | 0,025496278 | 0,399792436 | 0,063773789 | 9,49E-01 | 9,57E-01 |
| HUS00300548 | MiRBase | MIMAT0000085 | Homo_sapiens | mature_miRNA | hsa-miR-28-5p | 28 | AAGGAGCTCACAGTCTATTGAG | 3463 | 2208 | 1459 | 623 | 1685,167975 | 0,015081153 | 0,421451166 | 0,035783869 | 9,71E-01 | 9,77E-01 |
| HUS00304927 | MiRBase | MIMAT0003880 | Homo_sapiens | mature_miRNA | hsa-miR-671-5p | 671 | AGGAAGCCCTGGAGGGGCTGGAG | 126 | 57 | 41 | 23 | 53,30576872 | -0,016147075 | 0,465864462 | -0,034660457 | 9,72E-01 | 9,77E-01 |
| HUS00323553 | MiRBase | MIMAT0000075 | Homo_sapiens | mature_miRNA | hsa-miR-20a-5p | 17 | TAAAGTGCTTATAGTGCAGGTAG | 18300 | 19267 | 9703 | 4184 | 11267,53395 | 0,009315795 | 0,426717803 | 0,021831279 | 9,83E-01 | 9,85E-01 |
